# Excess of Rare Variants in Genes that are Key Epigenetic Regulators of Spermatogenesis in the Patients with Non-Obstructive Azoospermia

**DOI:** 10.1038/srep08785

**Published:** 2015-03-05

**Authors:** Zesong Li, Yi Huang, Honggang Li, Jingchu Hu, Xiao Liu, Tao Jiang, Guangqing Sun, Aifa Tang, Xiaojuan Sun, Weiping Qian, Yong Zeng, Jun Xie, Wei Zhao, Yu Xu, Tingting He, Chengliang Dong, Qunlong Liu, Lisha Mou, Jingxiao Lu, Zheguang Lin, Song Wu, Shengjie Gao, Guangwu Guo, Qiang Feng, Yingrui Li, Xiuqing Zhang, Jun Wang, Huanming Yang, Jian Wang, Chengliang Xiong, Zhiming Cai, Yaoting Gui

**Affiliations:** 1 Guangdong and Shenzhen Key Laboratory of Male Reproductive Medicine and Genetics, Institute of Urology, Peking University Shenzhen Hospital, Shenzhen PKU-HKUST Medical Center, Shenzhen 518036, China; 2 Shenzhen Key Laboratory of Genitourinary Cancer, Shenzhen Second People's Hospital, the First Affiliated Hospital of Shenzhen University, Shenzhen 518035, China; 3 National-Regional Engineering Laboratory for Clinical Application of Cancer Genomics, Shenzhen Second People's Hospital, the First Affiliated Hospital of Shenzhen University, Shenzhen 518035, China; 4 Family Planning Research Institute/The Center of Reproductive Medicine, Tongji Medical College, Huazhong University of Science and Technology, Wuhan 430030, China; 5 BGI-Shenzhen, Shenzhen 518083, China; 6 The Center of Reproductive Medicine, Peking University Shenzhen Hospital, Shenzhen 518036, China; 7 Shenzhen Key Laboratory of Reproductive Immunology for Peri-implantation, Shenzhen Zhongshan Urology Hospital, Shenzhen 518045, China

## Abstract

Non-obstructive azoospermia (NOA), a severe form of male infertility, is often suspected to be linked to currently undefined genetic abnormalities. To explore the genetic basis of this condition, we successfully sequenced ~650 infertility-related genes in 757 NOA patients and 709 fertile males. We evaluated the contributions of rare variants to the etiology of NOA by identifying individual genes showing nominal associations and testing the genetic burden of a given biological process as a whole. We found a significant excess of rare, non-silent variants in genes that are key epigenetic regulators of spermatogenesis, such as *BRWD1*, *DNMT1*, *DNMT3B*, *RNF17*, *UBR2*, *USP1* and *USP26*, in NOA patients (*P* = 5.5 × 10^−7^), corresponding to a carrier frequency of 22.5% of patients and 13.7% of controls (*P* = 1.4 × 10^−5^). An accumulation of low-frequency variants was also identified in additional epigenetic genes (*BRDT* and *MTHFR*). Our study suggested the potential associations of genetic defects in genes that are epigenetic regulators with spermatogenic failure in human.

Azoospermia affects approximately 1% of all men and over 15% of infertile men^1^. Non-obstructive azoospermia (NOA) is the most common form of azoospermia, and it is usually assumed to be caused by genetic defects^2^. Although genetic aberrations such as karyotype abnormalities and Y chromosome microdeletions have been identified in a small proportion of patients with NOA, the genetic causes for most patients remain largely unknown^3^,^4^. Mouse studies have effectively identified the genes that are involved in the reproductive processes, and hundreds of mouse mutants with spermatogenic defects have been produced in the past decades^4^. Nevertheless, there has been little progress in translating of these results from animal models into understanding the genetic causes of infertility in humans. Recently, SNP array-based association studies in clinical patients identified several common tag SNPs with relatively modest effects on NOA susceptibility^5^,^6^. However, reports on the identification of rare or low-frequency functional mutations in specific genes with relatively larger effects are scarce. Thus, extensive analysis of both the common and uncommon genetic variants for all genes implicated in the reproductive processes in large cohorts of infertile patients, especially in those with the extreme phenotype of azoospermia, would be of great value to uncover the genetics of human spermatogenic failure.

To this end, 766 NOA patients without known karyotype abnormalities or Y chromosome microdeletions and 717 proven fertile males from the same ethnic population were selected for analysis of genetic defects in other candidate genomic regions. We isolated DNA from the participants and sequenced the exonic regions of 654 genes, of which 299 have been reported to be associated with infertility in humans and 355 are related to spermatogenic disorders in knockout mice (Supplementary Material, Table S1)^4^. A comprehensive catalogue of genetic variants was generated in these candidate genes and the potential contribution of both the common and rare mutations to the predisposition of infertility in male humans was also evaluated.

## Results

### Identification and validation of the genetic variants

After several quality control steps (see Materials and method), 757 NOA patients and 709 fertile males remained for further analysis (Supplementary Material, Figure 1A and Table S2). A total of ~47.8 K genetic variants, including 13.3 K non-silent variants and 8.2 K silent coding variants, were identified in the combined population. Across all the genes, over 90% of the genetic variants were variants with minor allele frequencies (MAFs) less than 5% and over 85% were rare variants with MAFs less than 1%. We then randomly selected 75 novel variants (not reported as genetic polymorphisms in the public database dbSNP137) for validation with Sanger sequencing or Sequenom genotyping in 152 samples harboring these variants (Supplementary Material, Table S3). 73 of the 75 tested sites were proved to be true positives, corresponding to a false positive rate per site of 2.7%. Notably, novel variants in 147/152 of the tested samples were successfully validated, corresponding to a false positive rate for the SNV calls per individual of 3.3%.

### Single-marker association tests for common and low-frequency variants

To test whether there were any individual common (MAFs ≥ 5%) or low-frequency variants (1% ≤ MAFs < 5%) that would show associations with NOA, we performed the logistic regression analysis by including age as a covariate and applied a total of 10,000 permutations to determine the statistical significance. All the genetic variants showing significant differences in their call rates or depths of coverage between the patient and control groups were removed from association analysis (*P* < 10^−5^) to reduce the number of false positive associations. We observed that no single common or low-frequency variant remained significant after correcting for multiple tests (Supplementary Material, Figure S2). There was also no obvious trend of enrichment for common or low-frequency variants with relatively small *P* values in the patient group (Supplementary Material, Figure S3).

### Gene-based analysis of rare and low-frequency variants

It has been increasingly realized that the rare, higher-impact variants may play a significant role in the etiology of a variety of human diseases^7^,^8^,^9^,^10^. This may be especially true for the infertility related disorders because genetic variants that are strongly associated with infertility will scarcely be transmitted to offspring and will be less likely to spread in the general population^11^. Since the gene or gene-set based approaches usually provide improved power over the single variant tests^12^, we first performed the gene-based association tests for the rare variants (MAFs < 1%) alone and then with the rare variants and low-frequency variants (1% ≤ MAFs < 5%) together. The non-silent variants and variants in the UTRs in each gene were combined into two separate groups and the gene-based tests were performed for them independently. The adaptive permutation model provided by the PlinkSeq software was applied to obtain the empirical *P* values.

**Figure 1:**
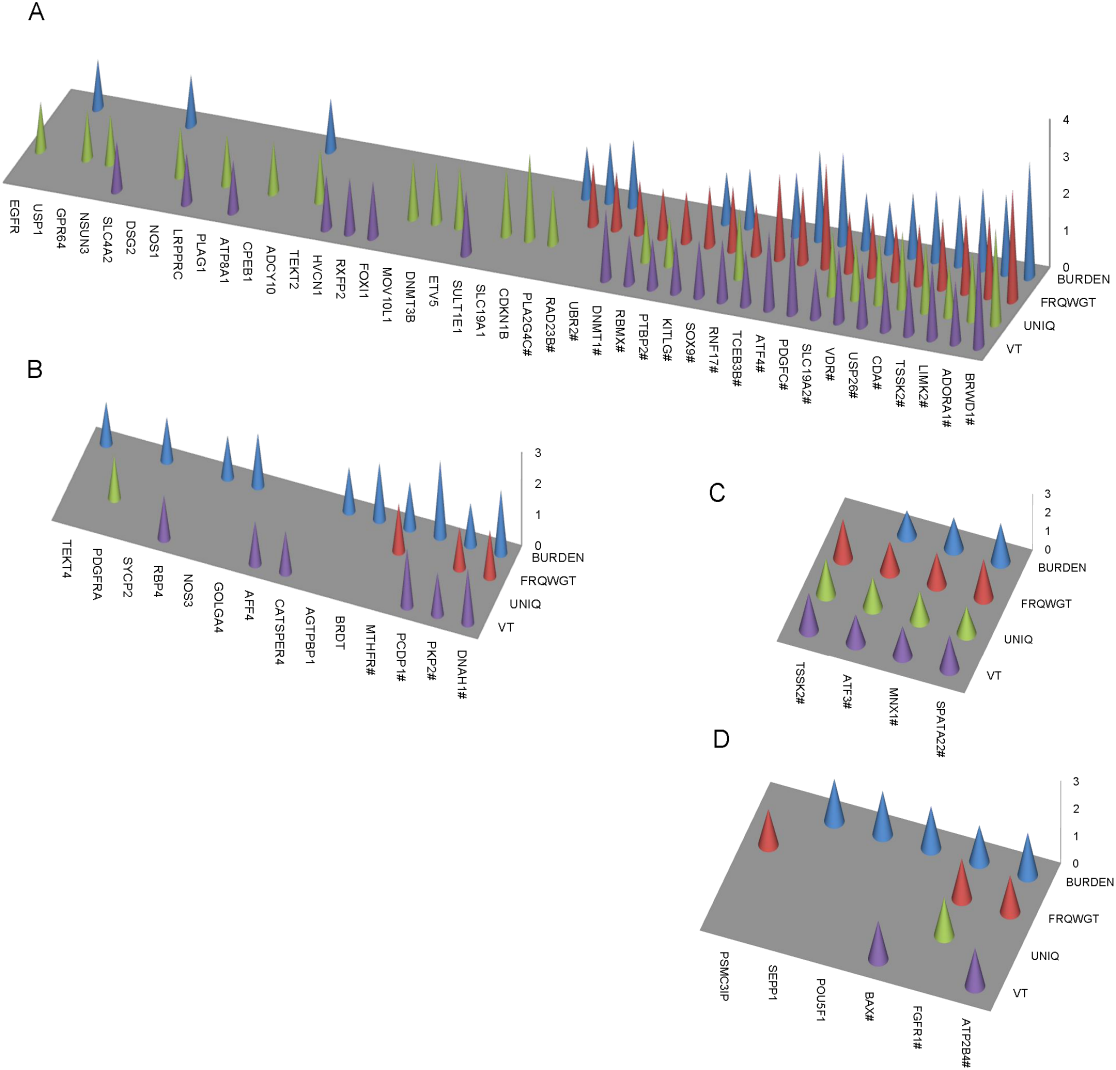
The nominally associated genes identified by four different gene-based tests. (A) Nominally associated gens identified by evaluation of rare non-silent variants in each gene. (B) Nominally associated genes identified by evaluation of both rare and low-frequency non-silent variants in each gene. (C) Nominally associated genes identified by evaluation of rare variants in the UTRs of each gene. (D) Nominally associated genes identified by evaluation of both rare and low-frequency variants in the UTRs of each gene. The height of each bar is proportional to the –log_10_ P value from the respective gene based test and P values ≥0.05 are not shown in the plots. # indicates the gene was determined to be nominally associated with NOA by at least two different tests.

A total of 42 genes were identified to be nominally associated with NOA by at least one of the four association tests, and nearly half (20/42) of the genes were determined to show nominal associations by at least two tests (permutated *P* < 0.05, Figure 1A). Also, none of the nominally associated genes individually could pass the threshold of multiple testing corrections (as 654 genes were sequenced). We then focused on the promising candidate genes that were ranked top by each gene-based analysis method in the list of candidate genes (Table 1 and Supplementary Material, Table S4). Almost all of the top ten genes identified by the BURDEN test showed significantly higher carrier frequency in the NOA patients than in the controls (Odds ratio (OR) = 1.65−4.25; *P* = 0.07−0.005). Five of the ten genes had been proved to affect fertility in mice (Table 1). Homozygous disruption of the transcription factor BRWD1, which showed the most significant excess of rare non-silent variants in NOA patients, results in a reduction in the counts of post meiotic spermatocytes and epididymal sperm in male mice^13^. Impairment of the *LIMK2* gene leads to small testes size and partial degeneration of spermatogenic cells in *LIMK2*-/- mice^14^.

We then analyzed whether there were any genes significantly enriched with case-unique variants and identified 24 genes showing an excess of rare, non-silent variants that were exclusive to NOA patients by the UNIQ test (Supplementary Material, Figure S4). For 75% (18/24) of these genes, at least 40% of the distinct variant loci could only be observed in NOA patients. We then expanded the control set to all those variants deposited in the public database dbSNP137, which records genetic variants from thousands of individuals of unknown clinical statuses. For 75% (18/24) of the genes showing enrichment for case-unique variants, over 60% of their case-unique variants was also absent in dbSNP137 (Supplementary Material, Figure S4). For example, four distinct missense variants were identified in *PTBP2*, all of which were exclusive to NOA patients and could not be found in dbSNP137. PTBP2 functions as a RNA-binding protein and is involved in the regulation of exon splicing and the stabilization of mRNA in mammalian testes^15^. Our genetic evidence showed that rare, non-silent variants in *PTBP2* were likely to be associated with NOA.

### Excess of rare and low-frequency variants in genes that are epigenetic regulator of spermatogenesis

**Figure 2:**
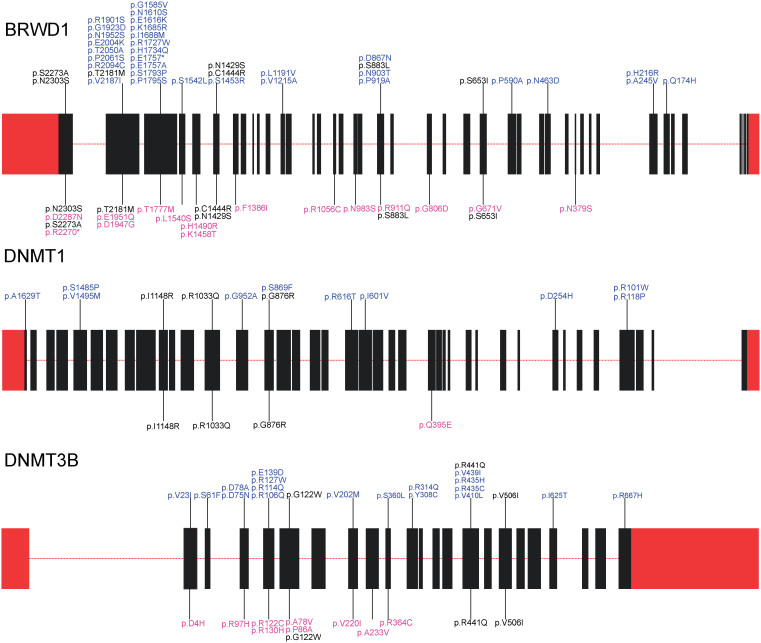
Rare non-silent variants identified in genes that are key epigenetic regulators of spermatogenesis. Variants shown above the indicated gene maps were detected in NOA patients, and variants shown below the indicated gene maps were detected in controls. Rare variants that were identified in both the patient and control groups are colored black, and rare variants that were exclusive to the NOA patients and normal controls are colored blue and pink, respectively. Boxes labeled in red represent the UTRs and boxes labeled in black represent the exons.

Notably, of these top genes and/or the genes showing an excess of case-unique variants, many are involved in regulating the dramatic changes in epigenetic profiles during spermatogenesis (Figure 2 and Supplementary Material, Figure S5). BRWD1, a chromatin remodeling protein with two bromodomains and multiple WD repeats, typically interacts with chromatin regions containing the hyperacetylated histones that are the characteristic marks of chromatin during early spermatogenesis^13^. UBR2, a RING finger E3 ubiquitin ligase, associates with meiotic chromatin regions and silences gene expression by ubiquitinating histone H2A^16^. RNF17, another RING finger protein, has been proved to be a key regulator of spermiogenesis and *RNF17-/-* male mice are infertile due to a complete arrest of spermatogenesis in the round spermatid stage^17^. USP26 is an ubiquitin-specific protease that cleaves the ubiquitin moiety from the ubiquitinylated proteins^18^. DNMT1 is an important DNA methylation enzyme capable of maintaining the established DNA methylation patterns, while DNMT3B is considered to be responsible for the establishment of *de novo* methylation^19^,^20^. NSUN3 is inferred to be a putative methyltransferase by sequence comparison analysis, and further studies are needed to confirm its *in vivo* functions.

When the gene-based analyses were further performed with non-silent variants with MAFs less than 5% (including both the low-frequency and rare variants), the association signals for the epigenetic genes mentioned above could be consistently detected. In addition, two other epigenetic genes also showed an excess of rare and low-frequency non-silent variants in the patients (Figure 1B). *BRDT* encodes another bromodomain containing protein which binds histiones with acetylated lysines and is implicated in chromatin remodeling. Previous studies demonstrated that male mice with defective *BRDT* produced fewer sperm and were sterile^21^. *MTHFR* encodes an essential enzyme for maintaining the bioavailability of methionine, which can be converted to the methyl donor, S-adenosylmethionine, for a number of biological processes such as the methylation of DNA and histones^22^.

### Pathway analysis of the nominally associated genes

The above findings suggested that some biological processes or pathways were likely to be enriched with genes that individually only nominally associated with NOA. To assess whether there was an enrichment of the nominally associated genes in any groups of genes or pathways, literature and database searches were used to compile several groups of genes or pathways from our list of candidate genes (Table 2). The large group of epigenetic genes was generally defined as those genes that are involved in biological processes relevant to histone modifications, chromatin remodeling and DNA methylation. The other groups of genes or pathways potentially relevant to the pathophysiology of NOA were defined by searching the related terms in the KEGG and PANTHER pathway databases. Hypergeometric tests were applied to determine the statistical significance of enrichment of the nominally associated genes in each pathway or group of genes. Our analysis indicated that the group of genes regulating the dynamic epigenetic changes during spermatogenesis was significantly enriched with multiple nominally associated genes (*P* = 0.002).

### The genetic burden of rare and low-frequency variants in the epigenetic genes

To further test the overall genetic burden of rare and low-frequency variants of these epigenetic genes in the NOA patients, all of the non-silent variants in the epigenetic genes that showed nominal associations with NOA were aggregated and the significance of accumulation of rare and low-frequency variants in the epigenetic genes as a whole was tested (Table 3). In total, 1.8 times more distinct rare variant loci in the NOA patients were detected than in the controls. We detected 197 rare variants in the NOA patients versus 105 in the normal controls (OR = 1.9; *P* = 5.5 × 10^−7^), corresponding to a carrier frequency of 22.5% in patients with NOA compared to 13.7% in the controls (*P* = 1.4 × 10^−5^). Of those variants exclusive to either the NOA patients or controls, 126 rare variants were case-unique variants while 49 were control-unique variants (OR = 2.5; *P* = 2.0 × 10^−8^). In our analysis of the rare and low-frequency variants together, the same trend of a significant excess of non-silent variants in the epigenetic genes were also noticed in the NOA patients (Table 3).

## Discussion

Previous association studies on NOA had been mainly focused on common genetic variants. In the present study, both common and uncommon (rare or low-frequency) genetic variants in ~650 genes related to the reproduction processes were analyzed for their association with NOA in a relatively large cohort of patients and controls. It's often interesting to investigate whether there are any genetic regions where both common and rare variants are associated with the disease phenotypes. In our study, no gene showed obvious trend of enrichment for the nominally associated common SNPs and rare (or low-frequency) non-silent variants (Supplementary Material, Figure S2). However, because only the coding sequences and flanking regions of the candidate genes were sequenced, it remains to be determined whether there is an enrichment of the common disease-associated intronic or intergenic SNPs relatively far from those genes showing an excess of rare non-silent variants.

Although a large number of the candidate genes investigated in the current study have been shown to cause male infertility in animal models^4^, our data demonstrated that most of the non-silent variants in these genes in the sporadic NOA patients were heterozygous missense mutations which could only be found in a few individuals, and few of the rare variants were truncating mutations resulting in loss of gene functions. Moreover, none of the candidate genes individually showed association signals that were significant enough to survive the multiple testing corrections. These findings suggested that the genetic causes for the majority of the sporadic NOA patients were quite complex. The gene-set or pathway-based association tests which can aggregate genetic evidence from multiple functionally related genes appear to be more appropriate and powerful for detecting the genetic associations. On the other hand, each pathway or gene-set consists of tens to hundreds of genes, and only some of them are associated with the disease. Therefore, the gene-based analyses may serve as the cornerstones of the gene-set or pathway based analyses. In this way, we evaluated the overall genetic burdens of several groups of genes and pathways. Our results showed that rare or low-frequency variants in genes that are key epigenetic regulators of spermatogenesis were significantly associated with NOA (Table 3), which provided new clues about the genetic causes of the epigenetic aberrations that occurred frequently in male patients with infertility or sub-fertility.

In fact, male germ cells show epigenetic profiles that are highly distinct from those of somatic cells and oocytes. During spermatogenesis, the epigenetic profiles of sperm genomes undergo extensive DNA demethylation, followed by DNA methylation and chromatin remodeling^23^,^24^. In maturing sperm cells, there is an additional process of chromatin remodeling, called the histone-to-protamine transition, which replaces most histones with protamines to compact chromatin^23^. Therefore, it is quite plausible that genetic defects in genes that are key epigenetic regulators (as caused by the rare or low-frequency variants) could lead to abnormal spermatogenesis^25^. There is enormous evidence in previous studies demonstrating that sperm samples from patients with oligozoospermia or oligoasthenoteratozoospermia often contain altered epigenetic modifications^26^,^27^. Our findings raised the possibility that genetic defects in genes that are the key epigenetic regulators of spermatogenesis were likely to contribute to these aberrant epigenetic profiles.

## Methods

### Sample preparation

Peripheral blood samples from all of the NOA patients and normal controls were collected from the Peking University Shenzhen Hospital and the Center of Reproductive Medicine, Tongji Medical College, Huazhong University of Science and Technology. The inclusion criteria for the NOA patients included the following: (i) no sperm detected in the pellets of semen samples on three different occasions; (ii) no inflammation or injury of the reproductive system or pelvic cavity; and (iii) no karyotypic abnormality or Y chromosome microdeletion. Y chromosome microdeletions were detected as described previously^28^. Testicular biopsy and histological analysis were conducted for the azoospermic men whenever possible. All of the control men had fathered at least one child without assisted reproductive techniques such as IVF, ICSI or IMSI. This study was approved by the ethical committees of Peking University Shenzhen Hospital and Tongji Medical College, and all participants signed a consent form permitting the collection and use of their blood samples in the study. The methods used in this study were carried out in accordance with the approved guidelines.

### Targeted sequencing

Genomic DNA was extracted and randomly fragmented by sonication to an average size of 250 bp. The resulting fragments were end repaired and A-tailed, then ligated to a pair of adaptors. The adaptor-ligated DNA products were amplified using index tagged primers in order to tag DNA from different individuals. The amplified products were purified with QIAquick PCR purification kits (QIAGEN) and hybridized to the custom-designed capture array as per NimbleGen's Sequence Capture protocol for enrichment. Each enriched library was loaded on the HiSeq 2000 platform and pair-end reads with the lengths of 90 bp were generated. The raw image files were processed by the Illumina Genome Analyzer Pipeline (v1.7) for base-calling with default parameters.

### Identification of genetic variants

Genetic variants that differ from the reference genome sequence were extracted as follows: (i) GLFv3 files that contain the genotype likelihoods of ten possible genotypes at each nucleotide site were generated for all individuals using SAM tools (v.0.1.16); (ii) the maximum likelihood estimate of population allele frequency was calculated from the genotype likelihoods using the expectation-maximization (EM) algorithm, and a SNP was called if two times the log likelihood ratio of the SNP model against the null model for a given genomic site was greater than 24 (likelihood ratio test); (iii) for each variant site, Bayesian-based genotype calling was performed for each individual by using the ML estimate of population allele frequency as a prior; and (iv) LD-based genotype calling was performed with BEAGLE using the candidate genetic variants and genotype likelihoods as input^29^.

Several filtering steps were then applied to reduce the false positives in variant calling. Candidate variant loci that met any one of the following criteria were filtered out: (i) base quality scores of the minor alleles significantly smaller than those of the major alleles (*P* < 10^−5^) when we performed rank sum tests across all individuals; (ii) loci showing significant strand biases for either the major or minor alleles across all individuals (*P* < 10^−5^); and (iii) loci with extremely high or low depths or of low mapping qualities. Moreover, those candidate variants with coverage less than 8× were also removed for each individual.

### Validation of genetic variants

Novel variants were randomly selected for validation with Sanger sequencing or Sequenom genotyping.

### Statistical analysis

To identify the common or low-frequency variants that are likely associated with NOA, the logistic regression analysis followed by permutation test were used to determine the statistical significance. Several complementary gene-based association tests included in the PlinkSeq software package were used to identify the candidate genes that are likely enriched with multiple functional mutations in the NOA patients. The BURDEN test compares the excess of minor alleles between patients and normal controls, while the FRQWGT test takes MAFs in controls as a weight function when comparing the excess of minor alleles. The VT test gives more weight to very rare variants, while the UNIQ test gives more weight to case-unique variants. The adaptive permutation model provided by the PlinkSeq software was applied to obtain the empirical *P* values. The hypergeometric test was applied to determine the statistical significance of enrichment of the nominally associated genes in each pathway or group of genes.

## Author Contributions

Y.G., Ji. W., Z.C., Z.L., C.X., H.Y. and Ju.W. managed the project. H.L., Z.L., A.T., X.S., W.Q., Y.Z., Q.L., L.M., J.X., J.L., Z.Lin and S.W. collected and prepared the samples. X.L., T.H., Q.F. W.Z. and X.Z. performed the sequencing. Y.G., Y.H., J.H., T.J., G.S., C.D., S.G., G.G., Y.X. and Y.L. performed the bioinformatic analysis. Z.L. and J.X. performed the PCR validation. Y.H., T.J., X.L. and Y.G. wrote the paper. Ji.W., Z.C., C.X., Ju.W. Z.L. and Y.H. revised the paper.

## Supplementary Material

Supplementary Information Supplementary Data

## Figures and Tables

**Table 1:** The top genes that showed an excess of rare, non-silent variants in the NOA patients

Gene	BURDEN	FRQWGT	UNIQ	VT	Carrier frequency (%)		OR	Fisher *P*
					Cases	Controls		
**BRWD1**^a^	7.4 × 10^−4^	9.9 × 10^−4^	2.7 × 10^−3^	1.5 × 10^−3^	7.27	4.09	1.84	0.01
PDGFC	3.6 × 10^−3^	1.6 × 10^−3^	--	3.8 × 10^−3^	2.51	0.71	3.62	0.007
ATF4	4.1 × 10^−3^	9.1 × 10^−3^	--	7.9 × 10^−3^	2.64	0.85	3.18	0.01
LIMK2^a^	6.7 × 10^−3^	8.1 × 10^−3^	4.1 × 10^−2^	9.5 × 10^−3^	3.43	1.13	3.11	0.005
ADORA1^a^	7.5 × 10^−3^	7.0 × 10^−3^	1.5 × 10^−2^	2.2 × 10^−2^	1.72	0.42	4.11	0.02
CDA	1.3 × 10^−2^	2.6 × 10^−2^	9.4 × 10^−3^	2.6 × 10^−2^	1.85	0.56	3.32	0.03
TSSK2^a^	1.4 × 10^−2^	1.4 × 10^−2^	1.5 × 10^−2^	2.0 × 10^−2^	2.38	0.85	2.85	0.02
**UBR2**^a^	1.9 × 10^−2^	3.0 × 10^−2^	--	--	5.68	3.39	1.97	0.04
**USP26**	2.0 × 10^−2^	3.2 × 10^−2^	1.5 × 10^−2^	2.0 × 10^−2^	1.19	0.28	4.25	0.07
TCEB3B	2.1 × 10^−2^	5.4 × 10^−3^	--	3.4 × 10^−2^	7.00	4.37	1.65	0.03
RAD23B^a^	2.6 × 10^−2^	2.3 × 10^−2^	--	--	1.19	0.28	4.25	0.07
SOX9	2.8 × 10^−2^	1.9 × 10^−2^	--	3.1 × 10^−2^	1.32	0.42	3.15	0.09
SLC19A2^a^	3.3 × 10^−2^	2.6 × 10^−2^	1.9 × 10^−2^	4.7 × 10^−2^	1.32	0.42	3.15	0.09
VDR^a^	4.5 × 10^−2^	2.1 × 10^−2^	2.9 × 10^−2^	1.1 × 10^−2^	1.45	0.56	2.60	0.12
SLC19A1^a^	--	--	1.6 × 10^−2^	--	1.85	0.99	1.89	0.19
CDKN1B^a^	--	--	4.9 × 10^−3^	--	1.32	0.56	2.36	0.18
ETV5^a^	--	--	2.3 × 10^−2^	--	0.66	0.14	4.70	0.22
**RNF17**^a^	--	4.5 × 10^−2^	1.4 × 10^−2^	2.2 × 10^−2^	3.70	2.54	1.47	0.23
**DNMT3B**	--	--	2.5 × 10^−2^	--	3.96	2.82	1.42	0.25
SULT1E1^a^	--	--	--	2.0 × 10^−2^	1.19	0.56	2.12	0.27
**DNMT1**	--	3.9 × 10^−2^	--	1.3 × 10^−2^	1.72	0.99	1.75	0.27

^a^genes proven to cause infertility in mouse mutants when deleted. Genes shown in bold are epigenetic regulators of spermatogenesis.

**Table 2:** Nominally associated genes detected in different groups of genes or pathways

Pathways or groups of genes*	# of genes sequenced	# of genes showing excessive of non-silent variants	*P*	FDR
Epigenetic genes	50	10	2.0 × 10^−3^	2.4 × 10^−2^
PI3 kinase pathway	11	3	1.0 × 10^−2^	6.0 × 10^−2^
GnRH signaling pathway	16	2	0.17	0.63
MAPK signaling pathway	26	3	0.26	0.63
Steroid hormone biosynthesis and metabolism	12	1	0.30	0.63
DNA repair or recombination	14	1	0.37	0.63
Cell cycle	14	1	0.37	0.63
Meiosis	19	1	0.53	0.73
Apoptosis	21	1	0.58	0.73
TGF signaling pathway	22	1	0.61	0.73
P53 signaling pathway	20	0	0.86	0.87
Wnt signaling pathway	21	0	0.87	0.87

^*^only those pathways or groups of genes that had more than 10 component genes sequenced were analyzed and shown.

**Table 3:** Accumulation of rare and low-frequency variants in the epigenetic genes in the NOA patients

		All variants		Exclusive variants		Distinctive loci	
		Cases	Controls	Cases	Controls	Cases	Controls
	Total alleles or samples	1514	1418	1514	1418	757	709
Rare variants	BRWD1	58	29	38	16	39	22
	DNMT1	13	7	10	1	13	4
	DNMT3B	30	20	20	10	21	12
	NSUN3	5	1	5	1	4	1
	RNF17	28	18	21	8	21	11
	UBR2	43	24	15	9	19	14
	USP1	11	4	9	3	9	4
	USP26	9	2	8	1	9	2
	Total variants or loci	197	105	126	49	135	70
		***P* = 5.5 × 10^−7^**		***P* = 2.0 × 10^−8^**		**R = 1.8**	
Rare and low-frequency variants	BRWD1	85	50	38	16	40	23
	BRDT	67	43	8	7	12	10
	DNMT1	13	7	10	1	13	4
	DNMT3B	30	20	20	10	21	12
	MTHFR	32	19	10	6	15	9
	NSUN3	5	1	5	1	4	1
	RNF17	98	88	21	8	23	13
	UBR2	43	24	15	9	19	14
	USP1	11	4	9	3	9	4
	USP26	9	2	8	1	9	2
	Total variants or loci	393	258	144	62	165	92
		***P* = 4.8 × 10^−7^**		***P* = 4.4 × 10^−8^**		**R = 1.7**	

Exclusive variants refer to non-silent variants detected exclusively in patients with NOA or exclusively in normal controls. Distinctive loci refer to the number of genomic sites affected by non-silent variants. R, the normalized ratio of the number of distinctive variant loci detected in NOA patients to the number of variant loci detected in controls. The two-sided *P* values were calculated by Fisher's exact test as described previously^30^.
