# Supplementary material for: Excess of Rare Variants in Genes that are Key Epigenetic Regulators of Spermatogenesis in the Patients with Non-Obstructive Azoospermia
Source: Sci Rep. 2015 Mar 5;5:8785. doi: 10.1038/srep08785 (PMC4350091; doi:10.1038/srep08785)
Supplement: Supplementary Information — Supplementary Data [file srep08785-s1.doc]

**Supplementary Data**

**Excess of rare variants in genes that are key epigenetic regulators of spermatogenesis in the patients with non-obstructive azoospermia**

Zesong Li, Yi Huang, Honggang Li, Jingchu Hu, Xiao Liu, Tao Jiang, Guangqing Sun, Aifa Tang, Xiaojuan Sun, Weiping Qian, Yong Zeng, Jun Xie, Wei Zhao, Yu Xu, Tingting He, Chengliang Dong, Qunlong Liu, Lisha Mou, Jingxiao Lu, Zheguang Lin, Song Wu, Shengjie Gao, Guangwu Guo, Qiang Feng, Yingrui Li, Xiuqing Zhang, Jun Wang, Huanming Yang, Jian Wang, Chengliang Xiong, Zhiming Cai &Yaoting Gui

**Data production and quality control**

After removing the sequencing adaptors and the low quality reads, the remaining sequencing data were aligned the to the human reference genome (NCBI build 37.1, hg19) using BWA[^7^](#_ENREF_7). Several individuals with poor sequencing depth or coverage (8×coverage <90% or mean depth <30) were removed from subsequent analysis (Supplementary Material Figure 1A and Table S2). Genetic variants in each candidate gene from each individual were called out and filtered as described in the materials and methods section below. The inbreeding coefficient and relatedness between samples were analyzed based on the genotype calls of the genetic variants and several samples were removed as outliers (Supplementary Material, Table S2). After these quality control steps, 757 NOA patients and 709 fertile males remained for further analysis, and the mean fractions of targeted bases that were covered by at least 8× were 99.0% and 99.2% in the case and control groups, respectively (Supplementary Material Figure 1B).

Figure S1. **Fold coverage of the targeted regions in all samples.** (A) The fractions of targeted bases covered by at least 1× or 8× in each individual. (B) The box plots show the fractions of targeted bases covered by at least 8× in the case and control groups, respectively.


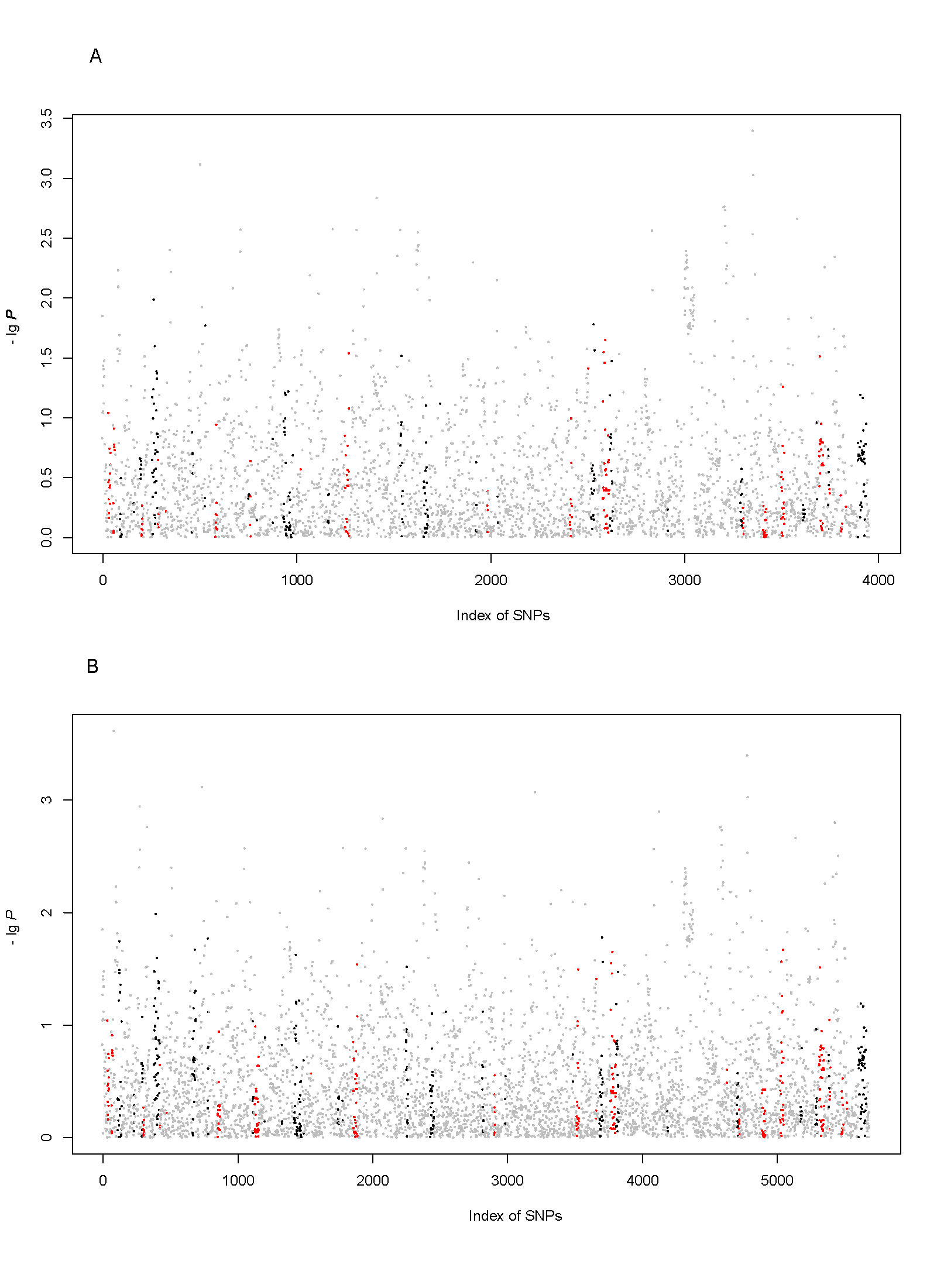


Figure S2 Single marker based association analysis of the common and low-frequency variants in the candidate genes. The scatter plot shows the –log_10_ *P* values from the logistic regression analyses. Genetic variants located in or around the nominally associated genes as determined by at least one gene-based test are labeled in black and genetic variants located in or around the nominally associated genes as determined by at least two gene-based tests are labeled in red. (A) The scatter plot shows the –log_10_ *P* values for the common variants. (B) The scatter plot shows the –log_10_ *P* values for the common and low-frequency variants.


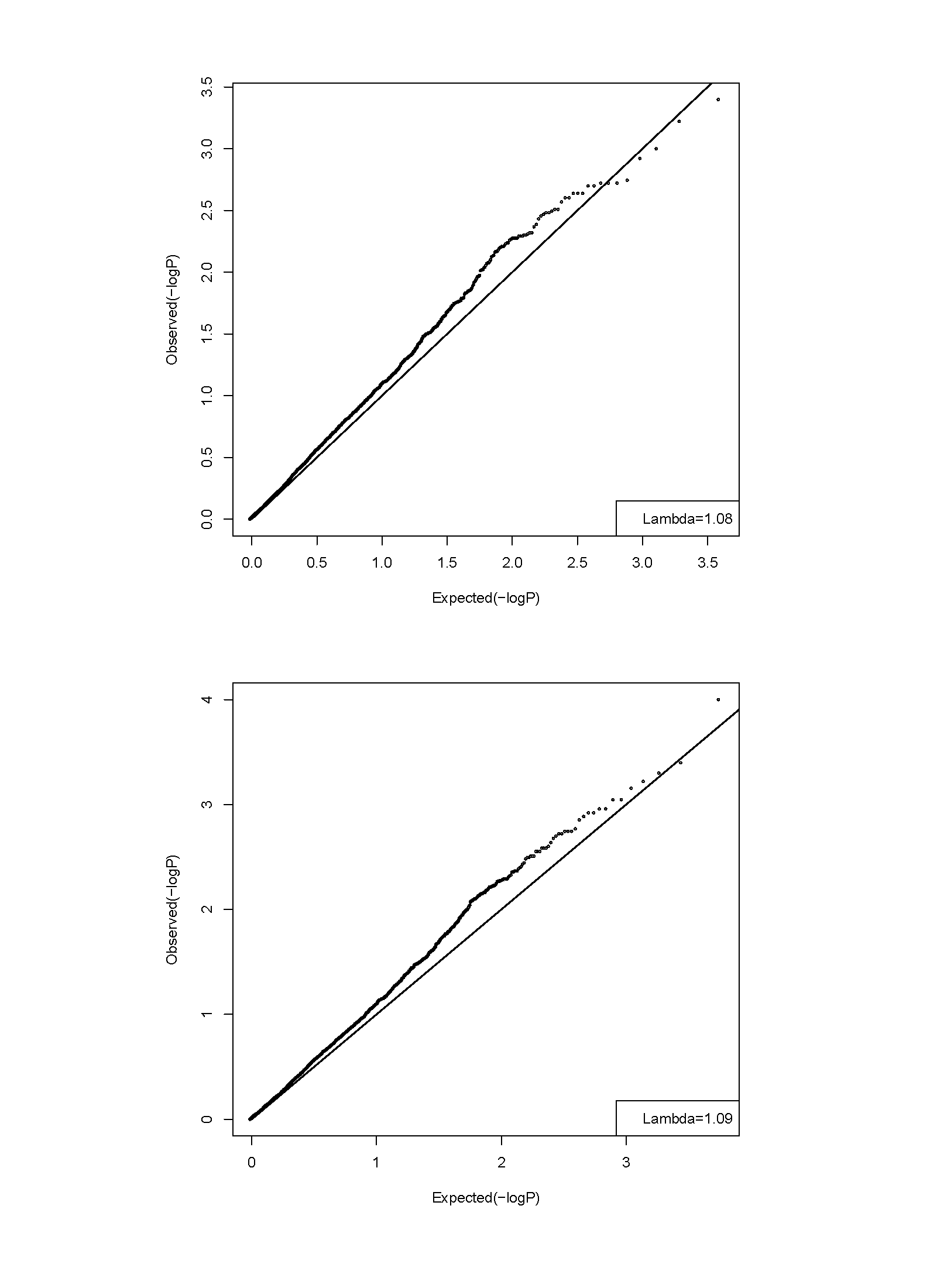


Figure S3 Quantile-Quantile plots. The –log_10_ *P* values were the permutated *P* values from the logistic regression analyses. The upper panel shows the results of the common variants and the lower panel shows the results of the common and low-frequency variants.

**
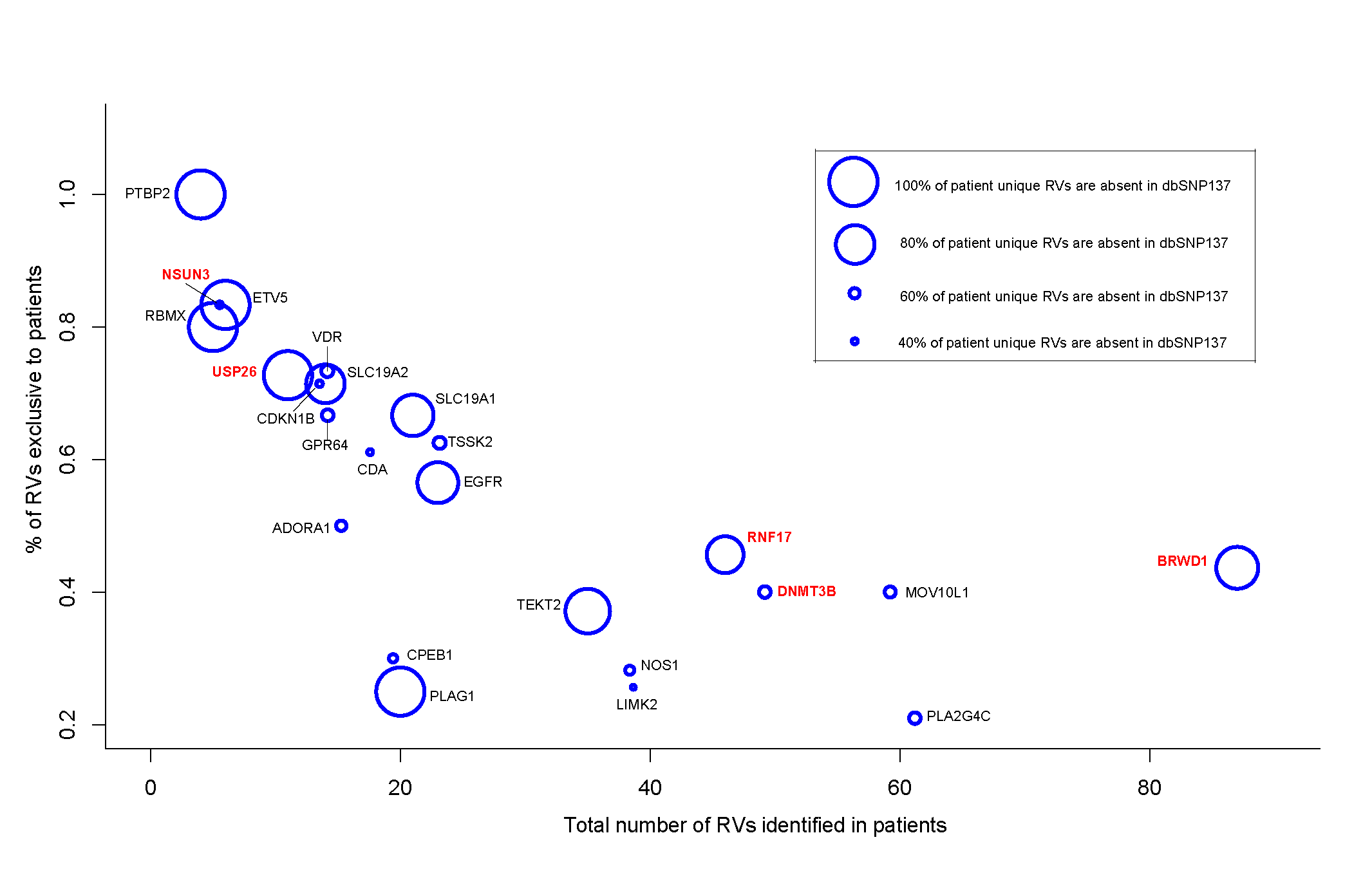
**

Figure S4 Genes showing significant excess of case-unique rare variants as determined by UNIQ test. Each circle in this chart represents a significant gene and the area of each circle is proportional to the percentage of case-unique RVs that are absent in dbSNP137. Genes regulating epigenetic changes during spermatogenesis are shown in red color.


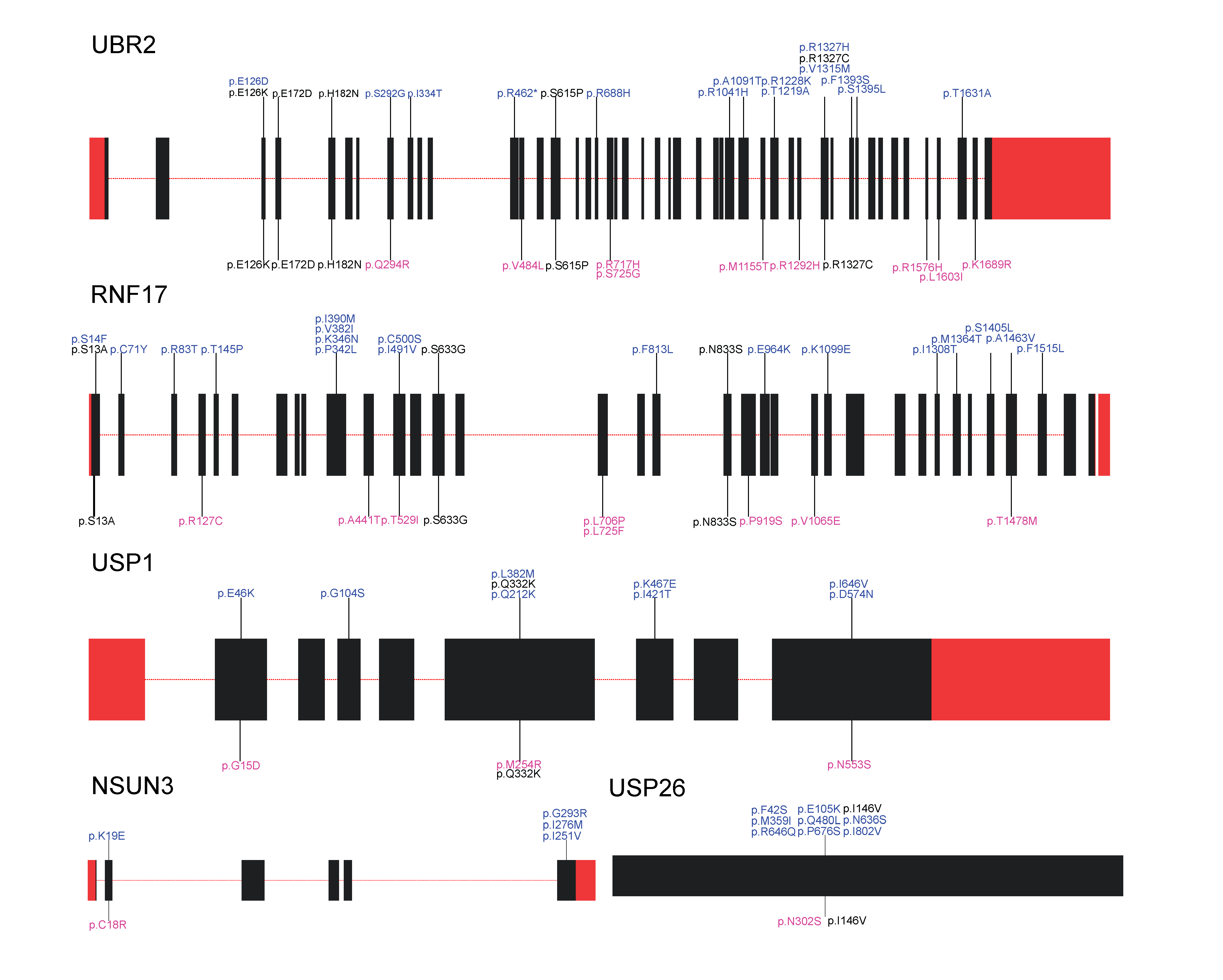


Figure S5 Rare non-silent variants identified in other key epigenetic regulators of spermatogenesis. Variants shown above the indicated gene maps were detected in NOA patients and variants shown below the indicated gene maps were detected in controls. Rare variants that were identified in both the patient and control groups are colored as black and rare variants that were exclusive to the NOA patients and normal controls are colored as blue and pink, respectively. Boxes labeled in red represent the UTRs and boxes labeled in black represent the exons.

Table S1 The full list of 654 infertility-related genes sequenced in our study

| Gene ID | Gene symbol | Gene size (Kb) | With infertile knockout mutants* | References |
| --- | --- | --- | --- | --- |
| 19 | ABCA1 | 6.8 | - | [1](#_ENREF_1) |
| 5243 | ABCB1 | 3.8 | - | [2](#_ENREF_2) |
| 1636 | ACE | 4.1 | Yes | [1](#_ENREF_1) |
| 92 | ACVR2A | 1.5 | Yes | [1](#_ENREF_1) |
| 132612 | ADAD1 | 1.7 | Yes | [1](#_ENREF_1) |
| 2515 | ADAM2 | 2.2 | Yes | [1](#_ENREF_1) |
| 9510 | ADAMTS1 | 2.9 | - | [1](#_ENREF_1) |
| 9509 | ADAMTS2 | 3.7 | Yes | [1](#_ENREF_1) |
| 55811 | ADCY10 | 4.8 | Yes | [1](#_ENREF_1) |
| 109 | ADCY3 | 3.4 | Yes | [1](#_ENREF_1) |
| 117 | ADCYAP1R1 | 1.4 | - | [1](#_ENREF_1) |
| 134 | ADORA1 | 1.0 | Yes | [1](#_ENREF_1) |
| 147 | ADRA1B | 1.6 | Yes | [1](#_ENREF_1) |
| 27125 | AFF4 | 3.5 | Yes | [1](#_ENREF_1) |
| 3267 | AGFG1 | 1.8 | Yes | [1](#_ENREF_1) |
| 23287 | AGTPBP1 | 3.6 | Yes | [1](#_ENREF_1) |
| 196 | AHR | 2.5 | Yes | [1](#_ENREF_1) |
| 57491 | AHRR | 2.2 | Yes | [1](#_ENREF_1) |
| 326 | AIRE | 1.9 | Yes | [1](#_ENREF_1) |
| 10566 | AKAP3 | 2.6 | - | [1](#_ENREF_1) |
| 8852 | AKAP4 | 2.6 | Yes | [1](#_ENREF_1) |
| 79087 | ALG12 | 1.5 | - | [1](#_ENREF_1) |
| 266 | AMELY | 0.6 | - | [3](#_ENREF_3) |
| 268 | AMH | 1.7 | Yes | [1](#_ENREF_1) |
| 269 | AMHR2 | 1.7 | - | [1](#_ENREF_1) |
| 317 | APAF1 | 3.7 | Yes | [1](#_ENREF_1) |
| 335 | APOA1 | 0.8 | Yes | [1](#_ENREF_1) |
| 338 | APOB | 13.7 | Yes | [1](#_ENREF_1) |
| 367 | AR | 2.8 | Yes | [1](#_ENREF_1) |
| 10124 | ARL4A | 0.6 | Yes | [1](#_ENREF_1) |
| 10123 | ARL4C | 0.6 | Yes | [1](#_ENREF_1) |
| 379 | ARL4D | 0.6 | Yes | [1](#_ENREF_1) |
| 84100 | ARL6 | 0.6 | Yes | [1](#_ENREF_1) |
| 406 | ARNTL | 1.9 | Yes | [1](#_ENREF_1) |
| 419 | ART3 | 1.2 | - | [1](#_ENREF_1) |
| 170302 | ARX | 1.7 | Yes | [1](#_ENREF_1) |
| 467 | ATF3 | 0.6 | - | [1](#_ENREF_1) |
| 468 | ATF4 | 1.1 | Yes | [1](#_ENREF_1) |
| 472 | ATM | 9.2 | Yes | [1](#_ENREF_1) |
| 493 | ATP2B4 | 3.8 | Yes | [1](#_ENREF_1) |
| 10396 | ATP8A1 | 3.6 | - | [4](#_ENREF_4) |
| 148229 | ATP8B3 | 4.0 | - | [4](#_ENREF_4) |
| 4287 | ATXN3 | 1.1 | - | [5](#_ENREF_5) |
| 6795 | AURKC | 0.9 | Yes | [1](#_ENREF_1) |
| 2583 | B4GALNT1 | 1.6 | Yes | [1](#_ENREF_1) |
| 2683 | B4GALT1 | 1.2 | Yes | [1](#_ENREF_1) |
| 578 | BAK1 | 0.6 | - | [5](#_ENREF_5) |
| 7917 | BAT3 | 3.4 | Yes | [1](#_ENREF_1) |
| 581 | BAX | 0.8 | Yes | [1](#_ENREF_1) |
| 582 | BBS1 | 1.8 | Yes | [1](#_ENREF_1) |
| 583 | BBS2 | 2.2 | Yes | [1](#_ENREF_1) |
| 585 | BBS4 | 1.6 | Yes | [1](#_ENREF_1) |
| 596 | BCL2 | 0.8 | - | [1](#_ENREF_1) |
| 598 | BCL2L1 | 0.7 | Yes | [1](#_ENREF_1) |
| 599 | BCL2L2 | 0.6 | Yes | [1](#_ENREF_1) |
| 604 | BCL6 | 2.1 | Yes | [1](#_ENREF_1) |
| 23743 | BHMT2 | 1.1 | - | [1](#_ENREF_1) |
| 79444 | BIRC7 | 0.9 | - | [1](#_ENREF_1) |
| 353500 | BMP8A | 1.2 | Yes | [1](#_ENREF_1) |
| 656 | BMP8B | 1.2 | Yes | [1](#_ENREF_1) |
| 66037 | BOLL | 0.9 | - | [1](#_ENREF_1) |
| 672 | BRCA1 | 5.7 | Yes | [1](#_ENREF_1) |
| 675 | BRCA2 | 10.3 | Yes | [1](#_ENREF_1) |
| 6046 | BRD2 | 2.4 | - | [1](#_ENREF_1) |
| 676 | BRDT | 2.8 | Yes | [1](#_ENREF_1) |
| 54014 | BRWD1 | 7.2 | Yes | [1](#_ENREF_1) |
| 682 | BSG | 1.2 | Yes | [1](#_ENREF_1) |
| 8945 | BTRC | 1.8 | Yes | [1](#_ENREF_1) |
| 701 | BUB1B | 3.2 | Yes | [1](#_ENREF_1) |
| 283310 | C12orf64 | 7.0 | - | [5](#_ENREF_5) |
| 126859 | C1orf125 | 3.0 | - | [5](#_ENREF_5) |
| 84066 | C1orf49 | 0.8 | - | [6](#_ENREF_6) |
| 113278 | C20orf54 | 1.4 | - | [5](#_ENREF_5) |
| 339669 | C22orf33 | 0.8 | - | [5](#_ENREF_5) |
| 92749 | C2orf39 | 2.2 | - | [5](#_ENREF_5) |
| 157869 | C8orf84 | 0.8 | - | [5](#_ENREF_5) |
| 10369 | CACNG2 | 1.0 | - | [5](#_ENREF_5) |
| 23705 | CADM1 | 1.3 | Yes | [1](#_ENREF_1) |
| 814 | CAMK4 | 1.4 | Yes | [1](#_ENREF_1) |
| 93661 | CAPZA3 | 0.9 | Yes | [7](#_ENREF_7) |
| 835 | CASP2 | 1.4 | - | [1](#_ENREF_1) |
| 117144 | CATSPER1 | 2.3 | Yes | [1](#_ENREF_1) |
| 117155 | CATSPER2 | 1.6 | Yes | [1](#_ENREF_1) |
| 347732 | CATSPER3 | 1.2 | Yes | [1](#_ENREF_1) |
| 378807 | CATSPER4 | 1.4 | Yes | [1](#_ENREF_1) |
| 875 | CBS | 1.7 | - | [1](#_ENREF_1) |
| 84318 | CCDC77 | 1.5 | - | [5](#_ENREF_5) |
| 6347 | CCL2 | 0.3 | Yes | [1](#_ENREF_1) |
| 8900 | CCNA1 | 1.4 | Yes | [1](#_ENREF_1) |
| 57820 | CCNB1IP1 | 0.8 | Yes | [1](#_ENREF_1) |
| 894 | CCND2 | 0.9 | Yes | [1](#_ENREF_1) |
| 100133941 | CD24 | 0.2 | - | [5](#_ENREF_5) |
| 966 | CD59 | 0.4 | Yes | [1](#_ENREF_1) |
| 975 | CD81 | 0.7 | - | [1](#_ENREF_1) |
| 928 | CD9 | 0.7 | - | [1](#_ENREF_1) |
| 978 | CDA | 0.4 | - | [5](#_ENREF_5) |
| 994 | CDC25B | 1.7 | - | [1](#_ENREF_1) |
| 1017 | CDK2 | 0.9 | Yes | [1](#_ENREF_1) |
| 1019 | CDK4 | 0.9 | - | [1](#_ENREF_1) |
| 1027 | CDKN1B | 0.6 | Yes | [1](#_ENREF_1) |
| 1028 | CDKN1C | 1.0 | Yes | [1](#_ENREF_1) |
| 1031 | CDKN2C | 0.5 | Yes | [1](#_ENREF_1) |
| 1032 | CDKN2D | 0.5 | Yes | [1](#_ENREF_1) |
| 1041 | CDSN | 1.6 | - | [5](#_ENREF_5) |
| 10658 | CELF1 | 1.5 | Yes | [1](#_ENREF_1) |
| 1059 | CENPB | 1.8 | Yes | [1](#_ENREF_1) |
| 1072 | CFL1 | 0.5 | - | [1](#_ENREF_1) |
| 1080 | CFTR | 4.4 | Yes | [1](#_ENREF_1) |
| 1081 | CGA | 0.4 | Yes | [1](#_ENREF_1) |
| 10519 | CIB1 | 0.6 | Yes | [1](#_ENREF_1) |
| 10370 | CITED2 | 0.8 | - | [1](#_ENREF_1) |
| 1159 | CKMT1B | 1.3 | - | [8](#_ENREF_8) |
| 1164 | CKS2 | 0.2 | Yes | [1](#_ENREF_1) |
| 1181 | CLCN2 | 2.7 | Yes | [4](#_ENREF_4) |
| 5010 | CLDN11 | 0.6 | Yes | [1](#_ENREF_1) |
| 10686 | CLDN16 | 0.9 | Yes | [1](#_ENREF_1) |
| 1047 | CLGN | 1.8 | Yes | [1](#_ENREF_1) |
| 29883 | CNOT7 | 0.9 | Yes | [1](#_ENREF_1) |
| 5067 | CNTN3 | 3.1 | - | [5](#_ENREF_5) |
| 79937 | CNTNAP3 | 3.9 | - | [1](#_ENREF_1) |
| 23242 | COBL | 3.8 | - | [5](#_ENREF_5) |
| 1363 | CPE | 1.4 | Yes | [1](#_ENREF_1) |
| 64506 | CPEB1 | 1.7 | Yes | [1](#_ENREF_1) |
| 10815 | CPLX1 | 0.4 | Yes | [1](#_ENREF_1) |
| 1390 | CREM | 1.3 | Yes | [1](#_ENREF_1) |
| 167 | CRISP1 | 0.8 | Yes | [1](#_ENREF_1) |
| 7180 | CRISP2 | 0.7 | - | [9](#_ENREF_9) |
| 23373 | CRTC1 | 2.0 | Yes | [1](#_ENREF_1) |
| 1415 | CRYBB2 | 0.6 | Yes | [1](#_ENREF_1) |
| 8531 | CSDA | 1.1 | Yes | [1](#_ENREF_1) |
| 1435 | CSF1 | 1.7 | Yes | [1](#_ENREF_1) |
| 1437 | CSF2 | 0.4 | Yes | [1](#_ENREF_1) |
| 1459 | CSNK2A2 | 1.1 | Yes | [1](#_ENREF_1) |
| 23283 | CSTF2T | 1.9 | Yes | [1](#_ENREF_1) |
| 1499 | CTNNB1 | 2.3 | Yes | [1](#_ENREF_1) |
| 1523 | CUX1 | 2.0 | Yes | [1](#_ENREF_1) |
| 1583 | CYP11A1 | 1.6 | Yes | [1](#_ENREF_1) |
| 1584 | CYP11B1 | 1.5 | - | [1](#_ENREF_1) |
| 1585 | CYP11B2 | 1.5 | - | [1](#_ENREF_1) |
| 1586 | CYP17A1 | 1.5 | Yes | [1](#_ENREF_1) |
| 1588 | CYP19A1 | 1.5 | Yes | [1](#_ENREF_1) |
| 1543 | CYP1A1 | 1.5 | - | [10](#_ENREF_10) |
| 1589 | CYP21A2 | 3.0 | - | [1](#_ENREF_1) |
| 1594 | CYP27B1 | 1.5 | Yes | [1](#_ENREF_1) |
| 246126 | CYorf15A | 0.4 | - | [11](#_ENREF_11) |
| 84663 | CYorf15B | 0.5 | - | [11](#_ENREF_11) |
| 26528 | DAZAP1 | 1.3 | Yes | [1](#_ENREF_1) |
| 1618 | DAZL | 1.0 | Yes | [1](#_ENREF_1) |
| 54165 | DCUN1D1 | 0.8 | - | [5](#_ENREF_5) |
| 4921 | DDR2 | 2.6 | Yes | [1](#_ENREF_1) |
| 29118 | DDX25 | 1.5 | Yes | [1](#_ENREF_1) |
| 8653 | DDX3Y | 2.0 | - | [1](#_ENREF_1) |
| 54514 | DDX4 | 2.2 | - | [1](#_ENREF_1) |
| 1718 | DHCR24 | 1.6 | Yes | [1](#_ENREF_1) |
| 50846 | DHH | 1.2 | Yes | [1](#_ENREF_1) |
| 56616 | DIABLO | 0.7 | - | [1](#_ENREF_1) |
| 23405 | DICER1 | 5.8 | - | [1](#_ENREF_1) |
| 27120 | DKKL1 | 0.7 | Yes | [12](#_ENREF_12) |
| 9787 | DLGAP5 | 2.6 | - | [1](#_ENREF_1) |
| 1746 | DLX2 | 1.0 | - | [5](#_ENREF_5) |
| 11144 | DMC1 | 1.0 | Yes | [1](#_ENREF_1) |
| 1761 | DMRT1 | 1.1 | Yes | [1](#_ENREF_1) |
| 63946 | DMRTC2 | 1.1 | Yes | [1](#_ENREF_1) |
| 25981 | DNAH1 | 12.8 | - | [1](#_ENREF_1) |
| 3301 | DNAJA1 | 1.2 | Yes | [1](#_ENREF_1) |
| 83544 | DNAL1 | 0.6 | - | [1](#_ENREF_1) |
| 373863 | DND1 | 1.1 | Yes | [1](#_ENREF_1) |
| 1786 | DNMT1 | 4.9 | - | [1](#_ENREF_1) |
| 1789 | DNMT3B | 2.6 | - | [1](#_ENREF_1) |
| 29947 | DNMT3L | 1.2 | Yes | [1](#_ENREF_1) |
| 81704 | DOCK8 | 6.3 | - | [5](#_ENREF_5) |
| 151871 | DPPA2 | 0.9 | - | [1](#_ENREF_1) |
| 1829 | DSG2 | 3.4 | - | [13](#_ENREF_13) |
| 1915 | EEF1A1 | 1.4 | - | [1](#_ENREF_1) |
| 84766 | EFCAB4B | 2.4 | - | [5](#_ENREF_5) |
| 1948 | EFNB2 | 1.0 | - | [1](#_ENREF_1) |
| 1956 | EGFR | 3.9 | - | [1](#_ENREF_1) |
| 1958 | EGR1 | 1.6 | Yes | [1](#_ENREF_1) |
| 1961 | EGR4 | 1.8 | Yes | [1](#_ENREF_1) |
| 10938 | EHD1 | 1.6 | - | [14](#_ENREF_14) |
| 9086 | EIF1AY | 0.4 | - | [15](#_ENREF_15) |
| 8672 | EIF4G3 | 4.8 | Yes | [16](#_ENREF_16) |
| 56648 | EIF5A2 | 0.5 | - | [1](#_ENREF_1) |
| 2018 | EMX2 | 0.8 | Yes | [1](#_ENREF_1) |
| 2034 | EPAS1 | 2.6 | Yes | [7](#_ENREF_7) |
| 2067 | ERCC1 | 1.0 | Yes | [1](#_ENREF_1) |
| 2068 | ERCC2 | 2.3 | - | [1](#_ENREF_1) |
| 2069 | EREG | 0.5 | - | [1](#_ENREF_1) |
| 2099 | ESR1 | 1.8 | Yes | [1](#_ENREF_1) |
| 2100 | ESR2 | 1.7 | Yes | [1](#_ENREF_1) |
| 80712 | ESX1 | 1.2 | - | [17](#_ENREF_17) |
| 2118 | ETV4 | 1.5 | Yes | [1](#_ENREF_1) |
| 2119 | ETV5 | 1.5 | Yes | [1](#_ENREF_1) |
| 2125 | EVPL | 6.1 | - | [5](#_ENREF_5) |
| 9156 | EXO1 | 2.5 | Yes | [1](#_ENREF_1) |
| 50848 | F11R | 0.9 | Yes | [1](#_ENREF_1) |
| 9415 | FADS2 | 1.3 | Yes | [5](#_ENREF_5) |
| 2175 | FANCA | 4.4 | Yes | [1](#_ENREF_1) |
| 2176 | FANCC | 1.7 | Yes | [1](#_ENREF_1) |
| 2189 | FANCG | 1.9 | Yes | [1](#_ENREF_1) |
| 55120 | FANCL | 1.1 | Yes | [1](#_ENREF_1) |
| 355 | FAS | 1.0 | - | [18](#_ENREF_18) |
| 356 | FASLG | 0.8 | - | [1](#_ENREF_1) |
| 2254 | FGF9 | 0.6 | Yes | [1](#_ENREF_1) |
| 2260 | FGFR1 | 2.6 | - | [1](#_ENREF_1) |
| 2263 | FGFR2 | 2.7 | - | [1](#_ENREF_1) |
| 2261 | FGFR3 | 2.6 | - | [19](#_ENREF_19) |
| 9457 | FHL5 | 0.9 | - | [1](#_ENREF_1) |
| 344018 | FIGLA | 0.7 | - | [1](#_ENREF_1) |
| 2288 | FKBP4 | 1.4 | Yes | [1](#_ENREF_1) |
| 8468 | FKBP6 | 1.0 | Yes | [1](#_ENREF_1) |
| 63943 | FKBPL | 1.1 | - | [20](#_ENREF_20) |
| 114793 | FMNL2 | 3.3 | - | [5](#_ENREF_5) |
| 22862 | FNDC3A | 3.6 | Yes | [1](#_ENREF_1) |
| 2353 | FOS | 1.1 | Yes | [1](#_ENREF_1) |
| 3171 | FOXA3 | 1.1 | Yes | [1](#_ENREF_1) |
| 27023 | FOXB1 | 1.0 | - | [1](#_ENREF_1) |
| 2299 | FOXI1 | 1.1 | - | [21](#_ENREF_21) |
| 668 | FOXL2 | 1.1 | - | [1](#_ENREF_1) |
| 2488 | FSHB | 0.4 | Yes | [1](#_ENREF_1) |
| 2492 | FSHR | 2.1 | Yes | [1](#_ENREF_1) |
| 10468 | FST | 1.0 | - | [1](#_ENREF_1) |
| 2521 | FUS | 1.6 | Yes | [1](#_ENREF_1) |
| 8087 | FXR1 | 1.9 | - | [22](#_ENREF_22) |
| 9514 | GAL3ST1 | 1.3 | Yes | [1](#_ENREF_1) |
| 2587 | GALR1 | 1.1 | - | [5](#_ENREF_5) |
| 2593 | GAMT | 1.0 | Yes | [1](#_ENREF_1) |
| 26330 | GAPDHS | 1.2 | Yes | [1](#_ENREF_1) |
| 57704 | GBA2 | 2.8 | Yes | [1](#_ENREF_1) |
| 151449 | GDF7 | 1.4 | Yes | [1](#_ENREF_1) |
| 2664 | GDI1 | 1.3 | Yes | [1](#_ENREF_1) |
| 2668 | GDNF | 0.7 | Yes | [1](#_ENREF_1) |
| 2678 | GGT1 | 1.7 | Yes | [1](#_ENREF_1) |
| 2690 | GHR | 1.9 | Yes | [1](#_ENREF_1) |
| 2697 | GJA1 | 1.1 | Yes | [1](#_ENREF_1) |
| 64395 | GMCL1 | 1.5 | Yes | [1](#_ENREF_1) |
| 2768 | GNA12 | 1.1 | - | [1](#_ENREF_1) |
| 10672 | GNA13 | 1.1 | - | [23](#_ENREF_23) |
| 2778 | GNAS | 4.0 | - | [1](#_ENREF_1) |
| 132789 | GNPDA2 | 0.8 | - | [24](#_ENREF_24) |
| 2796 | GNRH1 | 0.3 | Yes | [1](#_ENREF_1) |
| 2798 | GNRHR | 1.0 | Yes | [1](#_ENREF_1) |
| 2803 | GOLGA4 | 6.8 | - | [5](#_ENREF_5) |
| 57120 | GOPC | 1.4 | Yes | [1](#_ENREF_1) |
| 2842 | GPR19 | 1.2 | - | [5](#_ENREF_5) |
| 10149 | GPR64 | 3.1 | Yes | [1](#_ENREF_1) |
| 2879 | GPX4 | 0.9 | Yes | [25](#_ENREF_25) |
| 2944 | GSTM1 | 0.7 | - | [1](#_ENREF_1) |
| 2952 | GSTT1 | 0.7 | - | [1](#_ENREF_1) |
| 11036 | GTF2A1L | 1.4 | - | [1](#_ENREF_1) |
| 341567 | H1FNT | 0.8 | Yes | [1](#_ENREF_1) |
| 158983 | H2BFWT | 0.5 | - | [26](#_ENREF_26) |
| 3020 | H3F3A | 0.4 | Yes | [1](#_ENREF_1) |
| 3039 | HBA1 | 0.4 | - | [1](#_ENREF_1) |
| 3043 | HBB | 0.4 | - | [1](#_ENREF_1) |
| 3074 | HEXB | 1.7 | Yes | [1](#_ENREF_1) |
| 3077 | HFE | 1.0 | - | [27](#_ENREF_27) |
| 3092 | HIP1 | 3.1 | Yes | [1](#_ENREF_1) |
| 3159 | HMGA1 | 0.3 | Yes | [1](#_ENREF_1) |
| 8091 | HMGA2 | 0.4 | - | [1](#_ENREF_1) |
| 3148 | HMGB2 | 0.6 | Yes | [1](#_ENREF_1) |
| 6927 | HNF1A | 1.9 | Yes | [1](#_ENREF_1) |
| 6928 | HNF1B | 1.7 | - | [1](#_ENREF_1) |
| 51361 | HOOK1 | 2.2 | Yes | [1](#_ENREF_1) |
| 3206 | HOXA10 | 1.2 | Yes | [1](#_ENREF_1) |
| 3207 | HOXA11 | 0.9 | Yes | [1](#_ENREF_1) |
| 3209 | HOXA13 | 1.2 | - | [1](#_ENREF_1) |
| 3239 | HOXD13 | 1.0 | - | [1](#_ENREF_1) |
| 3293 | HSD17B3 | 0.9 | - | [28](#_ENREF_28) |
| 3284 | HSD3B2 | 1.1 | - | [1](#_ENREF_1) |
| 3298 | HSF2 | 1.6 | Yes | [1](#_ENREF_1) |
| 3306 | HSPA2 | 1.9 | Yes | [1](#_ENREF_1) |
| 22824 | HSPA4L | 2.5 | Yes | [1](#_ENREF_1) |
| 84329 | HVCN1 | 0.8 | - | [5](#_ENREF_5) |
| 3382 | ICA1 | 1.5 | - | [29](#_ENREF_29) |
| 130026 | ICA1L | 1.5 | - | [5](#_ENREF_5) |
| 3479 | IGF1 | 0.7 | Yes | [1](#_ENREF_1) |
| 3553 | IL1B | 0.8 | - | [1](#_ENREF_1) |
| 9173 | IL1RL1 | 1.7 | - | [1](#_ENREF_1) |
| 3557 | IL1RN | 0.6 | Yes | [5](#_ENREF_5) |
| 83943 | IMMP2L | 0.5 | Yes | [1](#_ENREF_1) |
| 3623 | INHA | 1.1 | Yes | [1](#_ENREF_1) |
| 3633 | INPP5B | 2.7 | Yes | [1](#_ENREF_1) |
| 3640 | INSL3 | 0.4 | Yes | [1](#_ENREF_1) |
| 3643 | INSR | 4.1 | - | [5](#_ENREF_5) |
| 9807 | IP6K1 | 1.3 | Yes | [1](#_ENREF_1) |
| 79711 | IPO4 | 3.2 | - | [5](#_ENREF_5) |
| 8660 | IRS2 | 4.0 | - | [1](#_ENREF_1) |
| 83700 | JAM3 | 1.1 | Yes | [1](#_ENREF_1) |
| 3720 | JARID2 | 3.7 | - | [1](#_ENREF_1) |
| 3727 | JUND | 1.0 | Yes | [1](#_ENREF_1) |
| 157855 | KCNU1 | 3.5 | Yes | [30](#_ENREF_30) |
| 55818 | KDM3A | 4.0 | Yes | [31](#_ENREF_31) |
| 8284 | KDM5D | 4.7 | - | [11](#_ENREF_11) |
| 10657 | KHDRBS1 | 1.3 | Yes | [32](#_ENREF_32) |
| 57576 | KIF17 | 3.1 | - | [5](#_ENREF_5) |
| 9493 | KIF23 | 2.9 | - | [33](#_ENREF_33) |
| 3814 | KISS1 | 0.4 | Yes | [1](#_ENREF_1) |
| 84634 | KISS1R | 1.2 | Yes | [1](#_ENREF_1) |
| 3815 | KIT | 2.9 | Yes | [1](#_ENREF_1) |
| 4254 | KITLG | 0.8 | - | [1](#_ENREF_1) |
| 687 | KLF9 | 0.7 | - | [1](#_ENREF_1) |
| 317719 | KLHL10 | 1.8 | Yes | [1](#_ENREF_1) |
| 9622 | KLK4 | 0.8 | - | [5](#_ENREF_5) |
| 3845 | KRAS | 0.7 | - | [1](#_ENREF_1) |
| 342574 | KRT27 | 1.4 | - | [5](#_ENREF_5) |
| 3908 | LAMA2 | 9.4 | - | [5](#_ENREF_5) |
| 3948 | LDHC | 1.0 | Yes | [1](#_ENREF_1) |
| 3952 | LEP | 0.5 | Yes | [1](#_ENREF_1) |
| 3953 | LEPR | 3.7 | Yes | [1](#_ENREF_1) |
| 3955 | LFNG | 1.4 | Yes | [1](#_ENREF_1) |
| 55366 | LGR4 | 2.9 | Yes | [1](#_ENREF_1) |
| 3972 | LHB | 0.4 | Yes | [1](#_ENREF_1) |
| 3973 | LHCGR | 2.1 | Yes | [1](#_ENREF_1) |
| 8022 | LHX3 | 1.3 | - | [1](#_ENREF_1) |
| 3985 | LIMK2 | 2.3 | Yes | [1](#_ENREF_1) |
| 3991 | LIPE | 3.2 | Yes | [1](#_ENREF_1) |
| 4000 | LMNA | 2.0 | Yes | [1](#_ENREF_1) |
| 22853 | LMTK2 | 4.5 | Yes | [1](#_ENREF_1) |
| 23566 | LPAR3 | 1.1 | - | [1](#_ENREF_1) |
| 57497 | LRFN2 | 2.4 | - | [5](#_ENREF_5) |
| 7804 | LRP8 | 2.9 | Yes | [1](#_ENREF_1) |
| 10128 | LRPPRC | 4.2 | - | [5](#_ENREF_5) |
| 23658 | LSM5 | 0.3 | - | [5](#_ENREF_5) |
| 4049 | LTA | 0.6 | - | [34](#_ENREF_34) |
| 4094 | MAF | 1.2 | - | [5](#_ENREF_5) |
| 10046 | MAMLD1 | 3.4 | - | [1](#_ENREF_1) |
| 4122 | MAN2A2 | 3.5 | Yes | [1](#_ENREF_1) |
| 9053 | MAP7 | 2.3 | Yes | [5](#_ENREF_5) |
| 2011 | MARK2 | 2.3 | Yes | [1](#_ENREF_1) |
| 5648 | MASP1 | 3.0 | - | [5](#_ENREF_5) |
| 9656 | MDC1 | 6.3 | Yes | [35](#_ENREF_35) |
| 150365 | MEI1 | 3.8 | Yes | [1](#_ENREF_1) |
| 644890 | MEIG1 | 0.3 | Yes | [5](#_ENREF_5) |
| 4232 | MEST | 1.0 | - | [1](#_ENREF_1) |
| 4240 | MFGE8 | 1.2 | Yes | [1](#_ENREF_1) |
| 4281 | MID1 | 2.4 | - | [1](#_ENREF_1) |
| 4292 | MLH1 | 2.3 | Yes | [1](#_ENREF_1) |
| 27030 | MLH3 | 4.4 | Yes | [1](#_ENREF_1) |
| 79258 | MMEL1 | 2.3 | Yes | [1](#_ENREF_1) |
| 3110 | MNX1 | 1.3 | Yes | [1](#_ENREF_1) |
| 27136 | MORC1 | 3.0 | Yes | [1](#_ENREF_1) |
| 54456 | MOV10L1 | 3.7 | Yes | [36](#_ENREF_36) |
| 117194 | MRGPRX2 | 1.0 | - | [5](#_ENREF_5) |
| 4438 | MSH4 | 2.8 | Yes | [1](#_ENREF_1) |
| 4439 | MSH5 | 2.6 | Yes | [1](#_ENREF_1) |
| 4524 | MTHFR | 2.0 | Yes | [1](#_ENREF_1) |
| 8898 | MTMR2 | 1.9 | Yes | [1](#_ENREF_1) |
| 4548 | MTR | 3.8 | Yes | [1](#_ENREF_1) |
| 4552 | MTRR | 2.2 | - | [1](#_ENREF_1) |
| 84939 | MUM1 | 2.1 | - | [37](#_ENREF_37) |
| 4603 | MYBL1 | 2.3 | Yes | [1](#_ENREF_1) |
| 79923 | NANOG | 0.9 | - | [1](#_ENREF_1) |
| 339345 | NANOS2 | 0.4 | Yes | [1](#_ENREF_1) |
| 342977 | NANOS3 | 0.6 | Yes | [1](#_ENREF_1) |
| 9 | NAT1 | 1.1 | - | [1](#_ENREF_1) |
| 89795 | NAV3 | 7.1 | - | [5](#_ENREF_5) |
| 4684 | NCAM1 | 2.8 | - | [1](#_ENREF_1) |
| 8648 | NCOA1 | 4.4 | - | [1](#_ENREF_1) |
| 23054 | NCOA6 | 6.2 | Yes | [1](#_ENREF_1) |
| 374291 | NDUFS7 | 0.6 | - | [5](#_ENREF_5) |
| 4728 | NDUFS8 | 0.6 | - | [5](#_ENREF_5) |
| 4780 | NFE2L2 | 1.8 | - | [38](#_ENREF_38) |
| 4808 | NHLH2 | 0.4 | Yes | [1](#_ENREF_1) |
| 22829 | NLGN4Y | 2.6 | - | [11](#_ENREF_11) |
| 338323 | NLRP14 | 3.3 | - | [1](#_ENREF_1) |
| 4842 | NOS1 | 4.3 | Yes | [1](#_ENREF_1) |
| 4846 | NOS3 | 3.9 | - | [1](#_ENREF_1) |
| 4864 | NPC1 | 3.8 | Yes | [1](#_ENREF_1) |
| 4867 | NPHP1 | 2.2 | Yes | [1](#_ENREF_1) |
| 4882 | NPR2 | 3.1 | - | [1](#_ENREF_1) |
| 190 | NR0B1 | 1.4 | YES | [1](#_ENREF_1) |
| 8431 | NR0B2 | 0.8 | - | [39](#_ENREF_39) |
| 4929 | NR4A2 | 1.8 | - | [5](#_ENREF_5) |
| 2516 | NR5A1 | 1.4 | Yes | [1](#_ENREF_1) |
| 4892 | NRAP | 5.2 | - | [5](#_ENREF_5) |
| 63899 | NSUN3 | 1.0 | - | [40](#_ENREF_40) |
| 79730 | NSUN7 | 2.2 | Yes | [1](#_ENREF_1) |
| 26471 | NUPR1 | 0.3 | Yes | [41](#_ENREF_41) |
| 11249 | NXPH2 | 0.8 | - | [42](#_ENREF_42) |
| 51686 | OAZ3 | 0.7 | - | [1](#_ENREF_1) |
| 4957 | ODF2 | 2.6 | Yes | [43](#_ENREF_43) |
| 343171 | OR2W3 | 0.9 | - | [44](#_ENREF_44) |
| 5013 | OTX1 | 1.1 | Yes | [1](#_ENREF_1) |
| 5017 | OVOL1 | 0.8 | Yes | [1](#_ENREF_1) |
| 5020 | OXT | 0.4 | - | [1](#_ENREF_1) |
| 5021 | OXTR | 1.2 | - | [1](#_ENREF_1) |
| 5023 | P2RX1 | 1.2 | Yes | [1](#_ENREF_1) |
| 135138 | PACRG | 0.9 | Yes | [1](#_ENREF_1) |
| 5048 | PAFAH1B1 | 1.2 | Yes | [1](#_ENREF_1) |
| 5049 | PAFAH1B2 | 0.9 | Yes | [1](#_ENREF_1) |
| 51247 | PAIP2 | 0.4 | Yes | [45](#_ENREF_45) |
| 56903 | PAPOLB | 1.9 | - | [46](#_ENREF_46) |
| 10038 | PARP2 | 1.8 | Yes | [1](#_ENREF_1) |
| 23598 | PATZ1 | 2.3 | - | [1](#_ENREF_1) |
| 5077 | PAX3 | 1.6 | - | [1](#_ENREF_1) |
| 7849 | PAX8 | 1.4 | Yes | [1](#_ENREF_1) |
| 57575 | PCDH10 | 3.2 | - | [5](#_ENREF_5) |
| 83259 | PCDH11Y | 4.1 | - | [11](#_ENREF_11) |
| 200373 | PCDP1 | 1.7 | Yes | [1](#_ENREF_1) |
| 54760 | PCSK4 | 2.3 | Yes | [1](#_ENREF_1) |
| 5130 | PCYT1A | 1.1 | - | [1](#_ENREF_1) |
| 9468 | PCYT1B | 1.2 | Yes | [1](#_ENREF_1) |
| 50940 | PDE11A | 3.0 | - | [47](#_ENREF_47) |
| 5139 | PDE3A | 3.4 | - | [1](#_ENREF_1) |
| 5154 | PDGFA | 0.6 | - | [48](#_ENREF_48) |
| 56034 | PDGFC | 1.0 | - | [5](#_ENREF_5) |
| 80310 | PDGFD | 1.1 | - | [48](#_ENREF_48) |
| 5156 | PDGFRA | 3.3 | - | [1](#_ENREF_1) |
| 2923 | PDIA3 | 1.5 | - | [1](#_ENREF_1) |
| 5037 | PEBP1 | 0.6 | Yes | [1](#_ENREF_1) |
| 10400 | PEMT | 0.7 | - | [1](#_ENREF_1) |
| 80055 | PGAP1 | 2.8 | Yes | [1](#_ENREF_1) |
| 9489 | PGS1 | 1.7 | Yes | [1](#_ENREF_1) |
| 7262 | PHLDA2 | 0.5 | - | [1](#_ENREF_1) |
| 9463 | PICK1 | 1.2 | Yes | [29](#_ENREF_29) |
| 5277 | PIGA | 1.5 | Yes | [1](#_ENREF_1) |
| 9271 | PIWIL1 | 2.6 | Yes | [1](#_ENREF_1) |
| 55124 | PIWIL2 | 2.9 | Yes | [1](#_ENREF_1) |
| 143689 | PIWIL4 | 2.6 | Yes | [1](#_ENREF_1) |
| 5585 | PKN1 | 2.9 | - | [5](#_ENREF_5) |
| 5318 | PKP2 | 2.6 | - | [5](#_ENREF_5) |
| 100137049 | PLA2G4B | 2.3 | - | [49](#_ENREF_49) |
| 8605 | PLA2G4C | 1.7 | Yes | [1](#_ENREF_1) |
| 5324 | PLAG1 | 1.5 | - | [50](#_ENREF_50) |
| 23236 | PLCB1 | 3.8 | Yes | [1](#_ENREF_1) |
| 84812 | PLCD4 | 2.3 | Yes | [1](#_ENREF_1) |
| 89869 | PLCZ1 | 1.8 | - | [51](#_ENREF_51) |
| 5351 | PLOD1 | 2.2 | - | [1](#_ENREF_1) |
| 5360 | PLTP | 1.5 | Yes | [1](#_ENREF_1) |
| 5395 | PMS2 | 2.6 | Yes | [1](#_ENREF_1) |
| 5447 | POR | 2.0 | - | [1](#_ENREF_1) |
| 5449 | POU1F1 | 1.0 | Yes | [1](#_ENREF_1) |
| 5460 | POU5F1 | 1.1 | - | [1](#_ENREF_1) |
| 134187 | POU5F2 | 1.0 | - | [52](#_ENREF_52) |
| 9677 | PPIP5K1 | 4.4 | - | [53](#_ENREF_53) |
| 8493 | PPM1D | 1.8 | Yes | [1](#_ENREF_1) |
| 5501 | PPP1CC | 1.0 | Yes | [1](#_ENREF_1) |
| 639 | PRDM1 | 2.5 | Yes | [1](#_ENREF_1) |
| 56979 | PRDM9 | 2.7 | - | [54](#_ENREF_54) |
| 5566 | PRKACA | 1.1 | Yes | [1](#_ENREF_1) |
| 5573 | PRKAR1A | 1.1 | Yes | [1](#_ENREF_1) |
| 5618 | PRLR | 1.9 | Yes | [1](#_ENREF_1) |
| 5619 | PRM1 | 0.2 | Yes | [1](#_ENREF_1) |
| 5620 | PRM2 | 0.3 | Yes | [1](#_ENREF_1) |
| 58531 | PRM3 | 0.3 | - | [55](#_ENREF_55) |
| 23627 | PRND | 0.5 | Yes | [1](#_ENREF_1) |
| 60675 | PROK2 | 0.4 | - | [56](#_ENREF_56) |
| 128674 | PROKR2 | 1.2 | - | [1](#_ENREF_1) |
| 5626 | PROP1 | 0.7 | Yes | [1](#_ENREF_1) |
| 10942 | PRSS21 | 0.9 | Yes | [57](#_ENREF_57) |
| 29893 | PSMC3IP | 0.7 | Yes | [1](#_ENREF_1) |
| 58155 | PTBP2 | 1.6 | - | [1](#_ENREF_1) |
| 5770 | PTPN1 | 1.3 | - | [1](#_ENREF_1) |
| 5781 | PTPN11 | 1.8 | - | [1](#_ENREF_1) |
| 11122 | PTPRT | 4.4 | - | [5](#_ENREF_5) |
| 5819 | PVRL2 | 2.0 | Yes | [1](#_ENREF_1) |
| 90780 | PYGO2 | 1.2 | Yes | [1](#_ENREF_1) |
| 5887 | RAD23B | 1.2 | Yes | [1](#_ENREF_1) |
| 5889 | RAD51C | 1.1 | Yes | [1](#_ENREF_1) |
| 5894 | RAF1 | 1.9 | - | [1](#_ENREF_1) |
| 5914 | RARA | 1.6 | Yes | [1](#_ENREF_1) |
| 5916 | RARG | 1.5 | Yes | [1](#_ENREF_1) |
| 54922 | RASIP1 | 2.9 | Yes | [1](#_ENREF_1) |
| 27316 | RBMX | 1.2 | - | [1](#_ENREF_1) |
| 27288 | RBMXL2 | 1.2 | Yes | [1](#_ENREF_1) |
| 5940 | RBMY1A1 | 4.5 | - | [1](#_ENREF_1) |
| 378948 | RBMY1B | 6.0 | - | [1](#_ENREF_1) |
| 378949 | RBMY1D | 6.0 | - | [1](#_ENREF_1) |
| 5950 | RBP4 | 0.6 | Yes | [1](#_ENREF_1) |
| 9985 | REC8 | 1.6 | Yes | [1](#_ENREF_1) |
| 5981 | RFC1 | 3.4 | - | [1](#_ENREF_1) |
| 26166 | RGS22 | 3.8 | - | [23](#_ENREF_23) |
| 85376 | RIMBP3 | 4.9 | Yes | 5 |
| 56163 | RNF17 | 4.9 | Yes | [1](#_ENREF_1) |
| 6098 | ROS1 | 7.0 | Yes | [1](#_ENREF_1) |
| 6103 | RPGR | 4.0 | Yes | [5](#_ENREF_5) |
| 6192 | RPS4Y1 | 0.8 | - | [11](#_ENREF_11) |
| 140032 | RPS4Y2 | 0.8 | - | [11](#_ENREF_11) |
| 284654 | RSPO1 | 0.8 | - | [1](#_ENREF_1) |
| 59350 | RXFP1 | 2.3 | Yes | [1](#_ENREF_1) |
| 122042 | RXFP2 | 2.3 | Yes | [1](#_ENREF_1) |
| 6257 | RXRB | 1.6 | Yes | [1](#_ENREF_1) |
| 6294 | SAFB | 2.7 | Yes | [58](#_ENREF_58) |
| 9667 | SAFB2 | 2.9 | - | [59](#_ENREF_59) |
| 6299 | SALL1 | 4.0 | - | [1](#_ENREF_1) |
| 57167 | SALL4 | 3.2 | - | [42](#_ENREF_42) |
| 6305 | SBF1 | 5.7 | Yes | [1](#_ENREF_1) |
| 6389 | SDHA | 2.0 | - | [1](#_ENREF_1) |
| 6414 | SEPP1 | 1.2 | Yes | [1](#_ENREF_1) |
| 124404 | SEPT12 | 1.1 | - | [60](#_ENREF_60) |
| 5414 | SEPT4 | 1.5 | Yes | [1](#_ENREF_1) |
| 5104 | SERPINA5 | 1.2 | Yes | [1](#_ENREF_1) |
| 5270 | SERPINE2 | 1.2 | Yes | [1](#_ENREF_1) |
| 151246 | SGOL2 | 3.8 | Yes | [1](#_ENREF_1) |
| 25970 | SH2B1 | 2.4 | Yes | [1](#_ENREF_1) |
| 153769 | SH3RF2 | 2.2 | - | [5](#_ENREF_5) |
| 6462 | SHBG | 1.2 | - | [1](#_ENREF_1) |
| 6477 | SIAH1 | 0.9 | - | [1](#_ENREF_1) |
| 6492 | SIM1 | 2.3 | - | [1](#_ENREF_1) |
| 23411 | SIRT1 | 2.2 | Yes | [1](#_ENREF_1) |
| 147912 | SIX5 | 2.2 | Yes | [1](#_ENREF_1) |
| 6558 | SLC12A2 | 3.6 | Yes | [1](#_ENREF_1) |
| 6573 | SLC19A1 | 1.8 | Yes | [61](#_ENREF_61) |
| 10560 | SLC19A2 | 1.5 | Yes | [1](#_ENREF_1) |
| 6505 | SLC1A1 | 1.6 | - | [62](#_ENREF_62) |
| 291 | SLC25A4 | 0.9 | Yes | [1](#_ENREF_1) |
| 6522 | SLC4A2 | 3.7 | Yes | [1](#_ENREF_1) |
| 285335 | SLC9A10 | 3.5 | Yes | [1](#_ENREF_1) |
| 114798 | SLITRK1 | 2.1 | Yes | [5](#_ENREF_5) |
| 4086 | SMAD1 | 1.4 | Yes | [1](#_ENREF_1) |
| 4090 | SMAD5 | 1.4 | Yes | [1](#_ENREF_1) |
| 27127 | SMC1B | 3.7 | Yes | [1](#_ENREF_1) |
| 4184 | SMCP | 0.4 | Yes | [1](#_ENREF_1) |
| 6609 | SMPD1 | 1.9 | Yes | [1](#_ENREF_1) |
| 114826 | SMYD4 | 2.4 | - | [5](#_ENREF_5) |
| 9892 | SNAP91 | 2.7 | - | [13](#_ENREF_13) |
| 6638 | SNRPN | 0.7 | - | [63](#_ENREF_63) |
| 6647 | SOD1 | 0.5 | - | [1](#_ENREF_1) |
| 402381 | SOHLH1 | 1.2 | Yes | [1](#_ENREF_1) |
| 54937 | SOHLH2 | 1.3 | Yes | [1](#_ENREF_1) |
| 6654 | SOS1 | 4.0 | - | [1](#_ENREF_1) |
| 64321 | SOX17 | 1.2 | - | [1](#_ENREF_1) |
| 6657 | SOX2 | 1.0 | - | [1](#_ENREF_1) |
| 6658 | SOX3 | 1.3 | - | [1](#_ENREF_1) |
| 30812 | SOX8 | 1.3 | Yes | [1](#_ENREF_1) |
| 6662 | SOX9 | 1.5 | - | [1](#_ENREF_1) |
| 6667 | SP1 | 2.4 | - | [1](#_ENREF_1) |
| 6671 | SP4 | 2.4 | Yes | [1](#_ENREF_1) |
| 10407 | SPAG11B | 1.0 | - | [64](#_ENREF_64) |
| 79582 | SPAG16 | 1.9 | Yes | [1](#_ENREF_1) |
| 9576 | SPAG6 | 1.5 | Yes | [1](#_ENREF_1) |
| 9043 | SPAG9 | 4.0 | Yes | [1](#_ENREF_1) |
| 6677 | SPAM1 | 1.6 | Yes | [1](#_ENREF_1) |
| 128153 | SPATA17 | 1.1 | - | [65](#_ENREF_65) |
| 84690 | SPATA22 | 1.1 | - | [66](#_ENREF_66) |
| 374768 | SPEM1 | 0.9 | Yes | [1](#_ENREF_1) |
| 57119 | SPINLW1 | 0.4 | - | [67](#_ENREF_67) |
| 23626 | SPO11 | 1.2 | Yes | [1](#_ENREF_1) |
| 81848 | SPRY4 | 1.0 | - | [5](#_ENREF_5) |
| 6714 | SRC | 1.6 | - | [1](#_ENREF_1) |
| 6715 | SRD5A1 | 0.8 | - | [1](#_ENREF_1) |
| 6736 | SRY | 0.6 | - | [1](#_ENREF_1) |
| 6745 | SSR1 | 0.9 | - | [5](#_ENREF_5) |
| 6770 | STAR | 0.9 | Yes | [1](#_ENREF_1) |
| 6774 | STAT3 | 2.3 | Yes | [1](#_ENREF_1) |
| 55342 | STRBP | 2.0 | Yes | [1](#_ENREF_1) |
| 161497 | STRC | 5.3 | - | [1](#_ENREF_1) |
| 2054 | STX2 | 0.9 | Yes | [1](#_ENREF_1) |
| 6815 | STYX | 0.7 | Yes | [1](#_ENREF_1) |
| 6783 | SULT1E1 | 0.9 | Yes | [1](#_ENREF_1) |
| 6847 | SYCP1 | 2.9 | Yes | [1](#_ENREF_1) |
| 10388 | SYCP2 | 4.6 | Yes | [1](#_ENREF_1) |
| 50511 | SYCP3 | 0.7 | Yes | [1](#_ENREF_1) |
| 6862 | T（T Brachyury Homolog) | 1.3 | - | [5](#_ENREF_5) |
| 6875 | TAF4B | 2.6 | Yes | [1](#_ENREF_1) |
| 54457 | TAF7L | 1.4 | Yes | [1](#_ENREF_1) |
| 6888 | TALDO1 | 1.0 | Yes | [1](#_ENREF_1) |
| 5726 | TAS2R38 | 1.0 | - | [5](#_ENREF_5) |
| 90665 | TBL1Y | 1.6 | - | [11](#_ENREF_11) |
| 9519 | TBPL1 | 0.6 | Yes | [1](#_ENREF_1) |
| 6926 | TBX3 | 2.2 | - | [1](#_ENREF_1) |
| 51224 | TCEB3B | 2.3 | - | [68](#_ENREF_68) |
| 6943 | TCF21 | 0.5 | Yes | [1](#_ENREF_1) |
| 6934 | TCF7L2 | 2.1 | - | [69](#_ENREF_69) |
| 6948 | TCN2 | 1.3 | - | [1](#_ENREF_1) |
| 6991 | TCTE3 | 0.6 | Yes | [70](#_ENREF_70) |
| 56165 | TDRD1 | 3.6 | Yes | [1](#_ENREF_1) |
| 83659 | TEKT1 | 1.3 | - | [1](#_ENREF_1) |
| 27285 | TEKT2 | 1.3 | Yes | [1](#_ENREF_1) |
| 64518 | TEKT3 | 1.5 | - | [1](#_ENREF_1) |
| 150483 | TEKT4 | 1.3 | Yes | [1](#_ENREF_1) |
| 26136 | TES | 1.3 | - | [71](#_ENREF_71) |
| 83639 | TEX101 | 0.8 | - | [64](#_ENREF_64) |
| 56159 | TEX11 | 2.9 | Yes | [1](#_ENREF_1) |
| 56155 | TEX14 | 4.5 | Yes | [1](#_ENREF_1) |
| 56154 | TEX15 | 8.4 | Yes | [1](#_ENREF_1) |
| 7040 | TGFB1 | 1.2 | Yes | [1](#_ENREF_1) |
| 90316 | TGIF2LX | 0.7 | - | [72](#_ENREF_72) |
| 90655 | TGIF2LY | 0.6 | - | [11](#_ENREF_11) |
| 7057 | THBS1 | 3.5 | - | [1](#_ENREF_1) |
| 51298 | THEG | 1.1 | Yes | [1](#_ENREF_1) |
| 9984 | THOC1 | 2.0 | Yes | [73](#_ENREF_73) |
| 7073 | TIAL1 | 1.2 | Yes | [1](#_ENREF_1) |
| 27283 | TINAG | 1.4 | - | [5](#_ENREF_5) |
| 9414 | TJP2 | 3.7 | - | [74](#_ENREF_74) |
| 7086 | TKT | 1.9 | - | [1](#_ENREF_1) |
| 9087 | TMSB4Y | 0.1 | - | [11](#_ENREF_11) |
| 7124 | TNF | 0.7 | - | [1](#_ENREF_1) |
| 7130 | TNFAIP6 | 0.8 | - | [1](#_ENREF_1) |
| 7132 | TNFRSF1A | 1.4 | - | [1](#_ENREF_1) |
| 8718 | TNFRSF25 | 1.3 | - | [5](#_ENREF_5) |
| 7141 | TNP1 | 0.2 | Yes | [1](#_ENREF_1) |
| 7142 | TNP2 | 0.4 | Yes | [1](#_ENREF_1) |
| 8940 | TOP3B | 2.6 | Yes | [1](#_ENREF_1) |
| 7157 | TP53 | 1.3 | Yes | [75](#_ENREF_75) |
| 7161 | TP73 | 2.0 | - | [76](#_ENREF_76) |
| 8459 | TPST2 | 1.1 | - | [77](#_ENREF_77) |
| 4591 | TRIM37 | 2.9 | - | [1](#_ENREF_1) |
| 9319 | TRIP13 | 1.3 | - | [1](#_ENREF_1) |
| 60385 | TSKS | 1.8 | - | [1](#_ENREF_1) |
| 7247 | TSN | 0.7 | Yes | [1](#_ENREF_1) |
| 7258 | TSPY1 | 0.9 | - | [1](#_ENREF_1) |
| 64591 | TSPY2 | 0.9 | - | [11](#_ENREF_11) |
| 728137 | TSPY3 | 1.9 | - | [11](#_ENREF_11) |
| 728395 | TSPY4 | 2.8 | - | [11](#_ENREF_11) |
| 7259 | TSPYL1 | 1.3 | - | [78](#_ENREF_78) |
| 23617 | TSSK2 | 1.1 | Yes | [79](#_ENREF_79) |
| 283629 | TSSK4 | 1.0 | - | [1](#_ENREF_1) |
| 83983 | TSSK6 | 0.8 | Yes | [1](#_ENREF_1) |
| 25809 | TTLL1 | 1.3 | Yes | [80](#_ENREF_80) |
| 51314 | TXNDC3 | 1.8 | - | [81](#_ENREF_81) |
| 7314 | UBB | 0.7 | Yes | [1](#_ENREF_1) |
| 7319 | UBE2A | 0.5 | - | [1](#_ENREF_1) |
| 7337 | UBE3A | 2.6 | Yes | [1](#_ENREF_1) |
| 7342 | UBP1 | 1.6 | - | [5](#_ENREF_5) |
| 23304 | UBR2 | 5.4 | Yes | [1](#_ENREF_1) |
| 7351 | UCP2 | 0.9 | - | [1](#_ENREF_1) |
| 7398 | USP1 | 2.4 | Yes | [82](#_ENREF_82) |
| 83844 | USP26 | 2.7 | - | [1](#_ENREF_1) |
| 8287 | USP9Y | 7.7 | - | [1](#_ENREF_1) |
| 8433 | UTF1 | 1.0 | - | [13](#_ENREF_13) |
| 9724 | UTP14C | 2.3 | - | [1](#_ENREF_1) |
| 7404 | UTY | 4.3 | - | [1](#_ENREF_1) |
| 8674 | VAMP4 | 0.4 | - | [83](#_ENREF_83) |
| 81839 | VANGL1 | 1.6 | - | [1](#_ENREF_1) |
| 7419 | VDAC3 | 0.9 | Yes | [1](#_ENREF_1) |
| 7421 | VDR | 1.3 | Yes | [1](#_ENREF_1) |
| 7434 | VIPR2 | 1.3 | Yes | [1](#_ENREF_1) |
| 7443 | VRK1 | 1.2 | - | [84](#_ENREF_84) |
| 7466 | WFS1 | 2.7 | Yes | [85](#_ENREF_85) |
| 644150 | WIPF3 | 1.5 | Yes | [1](#_ENREF_1) |
| 79971 | WLS | 1.7 | - | [5](#_ENREF_5) |
| 54361 | WNT4 | 1.1 | - | [1](#_ENREF_1) |
| 7476 | WNT7A | 1.1 | YES | [1](#_ENREF_1) |
| 7508 | XPC | 2.8 | - | [1](#_ENREF_1) |
| 7515 | XRCC1 | 1.9 | - | [1](#_ENREF_1) |
| 51087 | YBX2 | 1.1 | - | [1](#_ENREF_1) |
| 326340 | ZAR1 | 1.3 | - | [1](#_ENREF_1) |
| 7704 | ZBTB16 | 2.0 | Yes | [1](#_ENREF_1) |
| 678 | ZFP36L2 | 1.5 | - | [1](#_ENREF_1) |
| 7543 | ZFX | 2.5 | Yes | [1](#_ENREF_1) |
| 7544 | ZFY | 2.4 | Yes | [11](#_ENREF_11) |
| 100125288 | ZGLP1 | 0.8 | - | [86](#_ENREF_86) |
| 84225 | ZMYND15 | 2.2 | - | [87](#_ENREF_87) |
| 7761 | ZNF214 | 1.8 | - | [1](#_ENREF_1) |
| 7762 | ZNF215 | 1.6 | - | [1](#_ENREF_1) |
| 171017 | ZNF384 | 1.7 | - | [5](#_ENREF_5) |
| 22917 | ZP1 | 1.9 | - | [1](#_ENREF_1) |
| 7783 | ZP2 | 2.2 | - | [1](#_ENREF_1) |
| 7784 | ZP3 | 1.3 | - | [1](#_ENREF_1) |
| 11055 | ZPBP | 1.1 | Yes | [1](#_ENREF_1) |
| 124626 | ZPBP2 | 1.0 | Yes | [1](#_ENREF_1) |
| 146050 | ZSCAN29 | 2.6 | - | [5](#_ENREF_5) |

* The information is from published papers as of August, 2010

**Table S2 Quality control steps applied to remove outlier samples**

| Filter | Criteria | # of samples removed |
| --- | --- | --- |
| Mean sequencing depth | Fold coverage < 30× | 1 |
| Coverage | 1× coverage < 95% | 4 |
| Ratios of A/T, C/G | Outliers | 0 |
| GC content | Outliers | 0 |
| 18-barcode error | Number of discordance > 2 | 0 |
| Gender error | Predicted gender is inconsistent with gender record | 0 |
| Inbreeding coefficient | Coefficient >0.1 or < -0.1 | 7 |
| Relatedness | 1) Remove all samples showing relationships of monozygotic twin, parent-offspring, full-sib and 2^nd^ degree;  2) Remove samples which were related with at least 10 samples within 3^rd^ degree | 11 |

**Table S3 The frequency distribution of the novel variants that were selected for technical validation in our study.**

| Frequency  Category | < 0.1% | 0.1 ~ 0.5% | 0.5 ~ 1% | 1 ~ 5% | Total |
| --- | --- | --- | --- | --- | --- |
| 5'-UTR | 1 | - | - | - | 1 |
| 3'-UTR | 2 | 1 | - | - | 3 |
| Synonymous | 11 | 4 | 1 | 1 | 17 |
| Missense | 17 | 5 | 2 | - | 24 |
| Nonsense | 4 | 2 | - | - | 6 |
| Intronic | 12 | 11 | - | 1 | 24 |
| Total | 47 | 23 | 3 | 2 | 75 |

**Table S4. Distribution of genetic variants across the NOA associated genes that were ranked top by different gene based analyses.**

| **Chr** | **Position** | **Ref** | **Alt** | **Gene** | **Chain** | **Region** | **Function** | **MAF in controls** | **MAF in cases** |
| --- | --- | --- | --- | --- | --- | --- | --- | --- | --- |
| chr21 | 40558861 | G | T | BRWD1 | - | utr3 | silent | 0.001428571 | 0 |
| chr21 | 40558863 | A | G | BRWD1 | - | utr3 | silent | 0 | 0.001321004 |
| chr21 | 40558976 | T | C | BRWD1 | - | cds | synonymous | 0 | 0.002642008 |
| chr21 | 40559007 | T | C | BRWD1 | - | cds | missense | 0.005641749 | 0.005284016 |
| chr21 | 40559018 | T | C | BRWD1 | - | cds | synonymous | 0.002820874 | 0.003963012 |
| chr21 | 40559020 | G | A | BRWD1 | - | cds | synonymous | 0 | 0.002642008 |
| chr21 | 40559056 | C | T | BRWD1 | - | cds | missense | 0.002820874 | 0 |
| chr21 | 40559098 | A | C | BRWD1 | - | cds | missense | 0.001410437 | 0.005284016 |
| chr21 | 40559107 | G | A | BRWD1 | - | cds | nonsense | 0.001410437 | 0 |
| chr21 | 40559300 | T | A | BRWD1 | - | cds | synonymous | 0.001410437 | 0 |
| chr21 | 40559414 | C | A | BRWD1 | - | utr3 | silent | 0 | 0.001324503 |
| chr21 | 40559517 | A | G | BRWD1 | - | utr3 | silent | 0 | 0.002109705 |
| chr21 | 40559526 | T | C | BRWD1 | - | utr3 | silent | 0.003663004 | 0.009389671 |
| chr21 | 40568071 | A | G | BRWD1 | - | utr3 | silent | 0 | 0.001322751 |
| chr21 | 40568242 | A | G | BRWD1 | - | cds | synonymous | 0 | 0.001321004 |
| chr21 | 40568289 | T | C | BRWD1 | - | cds | missense | 0 | 0.001321004 |
| chr21 | 40568436 | C | T | BRWD1 | - | cds | missense | 0 | 0.001321004 |
| chr21 | 40568452 | C | A | BRWD1 | - | cds | synonymous | 0.001410437 | 0 |
| chr21 | 40568453 | G | A | BRWD1 | - | cds | missense | 0.001410437 | 0.003963012 |
| chr21 | 40568602 | C | T | BRWD1 | - | cds | synonymous | 0 | 0.001321004 |
| chr21 | 40568715 | G | A | BRWD1 | - | cds | missense | 0 | 0.003963012 |
| chr21 | 40568814 | G | A | BRWD1 | - | cds | missense | 0 | 0.001321004 |
| chr21 | 40568847 | T | C | BRWD1 | - | cds | missense | 0 | 0.001321004 |
| chr21 | 40568985 | C | T | BRWD1 | - | cds | missense | 0 | 0.002642008 |
| chr21 | 40569140 | T | C | BRWD1 | - | cds | missense | 0 | 0.001321004 |
| chr21 | 40569144 | C | G | BRWD1 | - | cds | missense | 0.001410437 | 0 |
| chr21 | 40569155 | T | C | BRWD1 | - | cds | missense | 0.001410437 | 0 |
| chr21 | 40569227 | C | T | BRWD1 | - | cds | missense | 0 | 0.002642008 |
| chr21 | 40569235 | G | A | BRWD1 | - | cds | synonymous | 0.001410437 | 0.002642008 |
| chr21 | 40569292 | C | G | BRWD1 | - | cds | missense | 0 | 0.001321004 |
| chr21 | 40569377 | G | C | BRWD1 | - | intron | silent | 0.012693935 | 0.007926024 |
| chr21 | 40569427 | G | A | BRWD1 | - | intron | silent | 0 | 0.001345895 |
| chr21 | 40569508 | T | C | BRWD1 | - | intron | silent | 0.434782609 | 0.485057471 |
| chr21 | 40569511 | G | A | BRWD1 | - | intron | silent | 0.064935065 | 0.036764706 |
| chr21 | 40570769 | T | A | BRWD1 | - | cds | missense | 0.029619182 | 0.035667107 |
| chr21 | 40570840 | A | G | BRWD1 | - | cds | synonymous | 0.001410437 | 0.001321004 |
| chr21 | 40570959 | G | A | BRWD1 | - | cds | missense | 0 | 0.001321004 |
| chr21 | 40570963 | A | G | BRWD1 | - | cds | synonymous | 0.008462623 | 0.009247028 |
| chr21 | 40570965 | A | G | BRWD1 | - | cds | missense | 0 | 0.001321004 |
| chr21 | 40571012 | G | A | BRWD1 | - | cds | missense | 0.001410437 | 0 |
| chr21 | 40571050 | A | G | BRWD1 | - | cds | synonymous | 0 | 0.001321004 |
| chr21 | 40571071 | C | T | BRWD1 | - | cds | synonymous | 0 | 0.002642008 |
| chr21 | 40571072 | T | G | BRWD1 | - | cds | missense | 0 | 0.002642008 |
| chr21 | 40571073 | C | A | BRWD1 | - | cds | nonsense | 0 | 0.002642008 |
| chr21 | 40571074 | A | G | BRWD1 | - | cds | synonymous | 0 | 0.002642008 |
| chr21 | 40571140 | A | C | BRWD1 | - | cds | missense | 0 | 0.001321004 |
| chr21 | 40571163 | G | A | BRWD1 | - | cds | missense | 0 | 0.001321004 |
| chr21 | 40571246 | A | G | BRWD1 | - | cds | missense | 0.114245416 | 0.14663144 |
| chr21 | 40571272 | T | C | BRWD1 | - | cds | synonymous | 0.005641749 | 0.003963012 |
| chr21 | 40571278 | A | C | BRWD1 | - | cds | missense | 0 | 0.001321004 |
| chr21 | 40571288 | T | C | BRWD1 | - | cds | missense | 0 | 0.001321004 |
| chr21 | 40571496 | C | T | BRWD1 | - | cds | missense | 0 | 0.001321004 |
| chr21 | 40571513 | T | C | BRWD1 | - | cds | missense | 0 | 0.001321004 |
| chr21 | 40571563 | A | G | BRWD1 | - | cds | synonymous | 0.031029619 | 0.022457067 |
| chr21 | 40571588 | C | A | BRWD1 | - | cds | missense | 0 | 0.001321004 |
| chr21 | 40571602 | T | G | BRWD1 | - | intron | silent | 0 | 0.001321004 |
| chr21 | 40571620 | G | C | BRWD1 | - | intron | silent | 0 | 0.001322751 |
| chr21 | 40571954 | T | C | BRWD1 | - | intron | silent | 0 | 0.001481481 |
| chr21 | 40572023 | T | A | BRWD1 | - | intron | silent | 0.001410437 | 0.002642008 |
| chr21 | 40572030 | C | T | BRWD1 | - | intron | silent | 0 | 0.001321004 |
| chr21 | 40572072 | G | A | BRWD1 | - | intron | silent | 0.056417489 | 0.038309115 |
| chr21 | 40572273 | G | A | BRWD1 | - | cds | missense | 0 | 0.001321004 |
| chr21 | 40572279 | A | G | BRWD1 | - | cds | missense | 0.001410437 | 0 |
| chr21 | 40572380 | A | G | BRWD1 | - | intron | silent | 0 | 0.001321004 |
| chr21 | 40572486 | G | A | BRWD1 | - | intron | silent | 0.001675042 | 0 |
| chr21 | 40574080 | C | T | BRWD1 | - | intron | silent | 0.117647059 | 0.147651007 |
| chr21 | 40574090 | C | T | BRWD1 | - | intron | silent | 0.1171875 | 0.148395722 |
| chr21 | 40574191 | C | T | BRWD1 | - | intron | silent | 0.056417489 | 0.040951123 |
| chr21 | 40574288 | T | C | BRWD1 | - | cds | synonymous | 0.001410437 | 0.001321004 |
| chr21 | 40574305 | A | G | BRWD1 | - | cds | missense | 0.136812412 | 0.161162483 |
| chr21 | 40574367 | T | C | BRWD1 | - | cds | missense | 0.001410437 | 0 |
| chr21 | 40574463 | T | G | BRWD1 | - | cds | missense | 0.001410437 | 0 |
| chr21 | 40574518 | A | T | BRWD1 | - | intron | silent | 0.001410437 | 0 |
| chr21 | 40574552 | G | T | BRWD1 | - | intron | silent | 0.305516266 | 0.294973545 |
| chr21 | 40574647 | T | C | BRWD1 | - | intron | silent | 0 | 0.001552795 |
| chr21 | 40574669 | T | C | BRWD1 | - | intron | silent | 0.1197411 | 0.146252285 |
| chr21 | 40577883 | G | A | BRWD1 | - | intron | silent | 0.334841629 | 0.342281879 |
| chr21 | 40578014 | G | A | BRWD1 | - | intron | silent | 0.14809591 | 0.14663144 |
| chr21 | 40578041 | T | G | BRWD1 | - | cds | missense | 0 | 0.001321004 |
| chr21 | 40578068 | A | G | BRWD1 | - | cds | missense | 0.002820874 | 0.00660502 |
| chr21 | 40578112 | T | C | BRWD1 | - | cds | missense | 0.001410437 | 0.001321004 |
| chr21 | 40578233 | A | T | BRWD1 | - | intron | silent | 0.001410437 | 0.007926024 |
| chr21 | 40578374 | A | T | BRWD1 | - | intron | silent | 0 | 0.0013947 |
| chr21 | 40581751 | G | A | BRWD1 | - | intron | silent | 0.053097345 | 0.038770053 |
| chr21 | 40581808 | G | A | BRWD1 | - | intron | silent | 0 | 0.002642008 |
| chr21 | 40581835 | A | G | BRWD1 | - | intron | silent | 0.001410437 | 0.001321004 |
| chr21 | 40581916 | A | G | BRWD1 | - | intron | silent | 0 | 0.001321004 |
| chr21 | 40581962 | A | T | BRWD1 | - | cds | missense | 0.001410437 | 0 |
| chr21 | 40582173 | G | A | BRWD1 | - | intron | silent | 0.001412429 | 0.001324503 |
| chr21 | 40582195 | A | T | BRWD1 | - | intron | silent | 0.001414427 | 0 |
| chr21 | 40582543 | A | G | BRWD1 | - | intron | silent | 0.00141844 | 0 |
| chr21 | 40582823 | C | T | BRWD1 | - | cds | synonymous | 0.031029619 | 0.019815059 |
| chr21 | 40582966 | T | G | BRWD1 | - | intron | silent | 0.001412429 | 0 |
| chr21 | 40583017 | G | A | BRWD1 | - | intron | silent | 0.115497076 | 0.148448043 |
| chr21 | 40583032 | A | G | BRWD1 | - | intron | silent | 0.007836991 | 0.011126565 |
| chr21 | 40584412 | A | T | BRWD1 | - | intron | silent | 0.306151645 | 0.296148738 |
| chr21 | 40584429 | C | T | BRWD1 | - | intron | silent | 0 | 0.00265252 |
| chr21 | 40584476 | T | G | BRWD1 | - | intron | silent | 0.001410437 | 0 |
| chr21 | 40584496 | C | T | BRWD1 | - | intron | silent | 0 | 0.002649007 |
| chr21 | 40584502 | T | C | BRWD1 | - | intron | silent | 0 | 0.001324503 |
| chr21 | 40584598 | C | T | BRWD1 | - | cds | synonymous | 0.114245416 | 0.14663144 |
| chr21 | 40584705 | A | G | BRWD1 | - | intron | silent | 0.001416431 | 0 |
| chr21 | 40585260 | G | C | BRWD1 | - | intron | silent | 0.001420455 | 0 |
| chr21 | 40585519 | G | C | BRWD1 | - | intron | silent | 0.001412429 | 0 |
| chr21 | 40585633 | G | A | BRWD1 | - | intron | silent | 0.349500713 | 0.314171123 |
| chr21 | 40585672 | G | T | BRWD1 | - | intron | silent | 0.001545595 | 0 |
| chr21 | 40587010 | G | A | BRWD1 | - | intron | silent | 0.015918958 | 0.026560425 |
| chr21 | 40587049 | A | G | BRWD1 | - | intron | silent | 0.11440678 | 0.14663144 |
| chr21 | 40587176 | A | G | BRWD1 | - | cds | synonymous | 0.001410437 | 0 |
| chr21 | 40587276 | C | A | BRWD1 | - | cds | synonymous | 0 | 0.001321004 |
| chr21 | 40587333 | T | C | BRWD1 | - | intron | silent | 0.005641749 | 0.002642008 |
| chr21 | 40587410 | A | G | BRWD1 | - | intron | silent | 0 | 0.001322751 |
| chr21 | 40587469 | C | T | BRWD1 | - | intron | silent | 0.001488095 | 0 |
| chr21 | 40587484 | G | C | BRWD1 | - | intron | silent | 0 | 0.001445087 |
| chr21 | 40590058 | G | C | BRWD1 | - | intron | silent | 0 | 0.001321004 |
| chr21 | 40590093 | A | G | BRWD1 | - | cds | missense | 0 | 0.001321004 |
| chr21 | 40590137 | T | C | BRWD1 | - | cds | synonymous | 0 | 0.001321004 |
| chr21 | 40590166 | A | C | BRWD1 | - | cds | missense | 0 | 0.001321004 |
| chr21 | 40590271 | T | A | BRWD1 | - | intron | silent | 0.001410437 | 0.001321004 |
| chr21 | 40590326 | A | G | BRWD1 | - | intron | silent | 0.001410437 | 0 |
| chr21 | 40590432 | A | G | BRWD1 | - | cds | synonymous | 0 | 0.001321004 |
| chr21 | 40590645 | C | T | BRWD1 | - | intron | silent | 0 | 0.001322751 |
| chr21 | 40590647 | G | T | BRWD1 | - | intron | silent | 0 | 0.001322751 |
| chr21 | 40596191 | G | A | BRWD1 | - | intron | silent | 0 | 0.002659574 |
| chr21 | 40596198 | T | A | BRWD1 | - | intron | silent | 0.004285714 | 0 |
| chr21 | 40596837 | T | C | BRWD1 | - | intron | silent | 0.001432665 | 0 |
| chr21 | 40596839 | G | C | BRWD1 | - | intron | silent | 0 | 0.002645503 |
| chr21 | 40596865 | A | C | BRWD1 | - | intron | silent | 0 | 0.001321004 |
| chr21 | 40596898 | G | A | BRWD1 | - | intron | silent | 0.016925247 | 0.015852048 |
| chr21 | 40596962 | C | G | BRWD1 | - | intron | silent | 0 | 0.001321004 |
| chr21 | 40597041 | A | G | BRWD1 | - | cds | synonymous | 0.008462623 | 0.009247028 |
| chr21 | 40597136 | T | A | BRWD1 | - | intron | silent | 0 | 0.001321004 |
| chr21 | 40597197 | C | T | BRWD1 | - | intron | silent | 0.001410437 | 0 |
| chr21 | 40597234 | A | G | BRWD1 | - | intron | silent | 0.042313117 | 0.035667107 |
| chr21 | 40597236 | A | T | BRWD1 | - | intron | silent | 0.310296192 | 0.278731836 |
| chr21 | 40600277 | A | T | BRWD1 | - | intron | silent | 0.001410437 | 0 |
| chr21 | 40600313 | T | C | BRWD1 | - | intron | silent | 0.115655853 | 0.149668874 |
| chr21 | 40600468 | G | A | BRWD1 | - | cds | missense | 0.001410437 | 0 |
| chr21 | 40600600 | G | A | BRWD1 | - | intron | silent | 0.001412429 | 0 |
| chr21 | 40600682 | A | G | BRWD1 | - | intron | silent | 0 | 0.001402525 |
| chr21 | 40600693 | A | G | BRWD1 | - | intron | silent | 0.003478261 | 0 |
| chr21 | 40600699 | T | C | BRWD1 | - | intron | silent | 0.003636364 | 0.006134969 |
| chr21 | 40601147 | T | G | BRWD1 | - | intron | silent | 0 | 0.001321004 |
| chr21 | 40601318 | C | A | BRWD1 | - | cds | synonymous | 0 | 0.003963012 |
| chr21 | 40601348 | A | G | BRWD1 | - | cds | synonymous | 0.001410437 | 0.001321004 |
| chr21 | 40603930 | C | T | BRWD1 | - | intron | silent | 0.115044248 | 0.14516129 |
| chr21 | 40604014 | G | A | BRWD1 | - | intron | silent | 0.001414427 | 0.001322751 |
| chr21 | 40604058 | G | A | BRWD1 | - | intron | silent | 0.002820874 | 0 |
| chr21 | 40604155 | T | C | BRWD1 | - | cds | missense | 0.001410437 | 0 |
| chr21 | 40604429 | C | G | BRWD1 | - | intron | silent | 0.445698166 | 0.476882431 |
| chr21 | 40604435 | G | A | BRWD1 | - | intron | silent | 0.001410437 | 0 |
| chr21 | 40604483 | G | C | BRWD1 | - | intron | silent | 0.001410437 | 0.001321004 |
| chr21 | 40604607 | G | A | BRWD1 | - | intron | silent | 0 | 0.002877698 |
| chr21 | 40604610 | T | C | BRWD1 | - | intron | silent | 0 | 0.001466276 |
| chr21 | 40608418 | C | A | BRWD1 | - | intron | silent | 0 | 0.001322751 |
| chr21 | 40608477 | G | C | BRWD1 | - | intron | silent | 0 | 0.001321004 |
| chr21 | 40608532 | G | C | BRWD1 | - | cds | missense | 0 | 0.001321004 |
| chr21 | 40608555 | C | T | BRWD1 | - | cds | missense | 0.001410437 | 0 |
| chr21 | 40608579 | T | G | BRWD1 | - | cds | missense | 0 | 0.001321004 |
| chr21 | 40608639 | G | A | BRWD1 | - | cds | missense | 0.004231312 | 0.002642008 |
| chr21 | 40608688 | C | T | BRWD1 | - | cds | missense | 0 | 0.001321004 |
| chr21 | 40610329 | G | A | BRWD1 | - | intron | silent | 0.005641749 | 0.009247028 |
| chr21 | 40610370 | A | T | BRWD1 | - | intron | silent | 0 | 0.001321004 |
| chr21 | 40610371 | A | T | BRWD1 | - | intron | silent | 0 | 0.001321004 |
| chr21 | 40610527 | T | A | BRWD1 | - | intron | silent | 0.002820874 | 0.001321004 |
| chr21 | 40619428 | C | A | BRWD1 | - | intron | silent | 0 | 0.00140056 |
| chr21 | 40619529 | G | A | BRWD1 | - | intron | silent | 0.001410437 | 0 |
| chr21 | 40619589 | T | C | BRWD1 | - | intron | silent | 0.007052186 | 0.005284016 |
| chr21 | 40619602 | T | C | BRWD1 | - | intron | silent | 0.001410437 | 0.001321004 |
| chr21 | 40619657 | A | G | BRWD1 | - | cds | synonymous | 0 | 0.001321004 |
| chr21 | 40619691 | C | T | BRWD1 | - | cds | missense | 0.001410437 | 0 |
| chr21 | 40619717 | T | G | BRWD1 | - | cds | synonymous | 0.001410437 | 0.002642008 |
| chr21 | 40619887 | G | A | BRWD1 | - | intron | silent | 0.056417489 | 0.041059603 |
| chr21 | 40619927 | T | C | BRWD1 | - | intron | silent | 0.00286123 | 0.002684564 |
| chr21 | 40622546 | A | G | BRWD1 | - | intron | silent | 0 | 0.001336898 |
| chr21 | 40622673 | A | G | BRWD1 | - | intron | silent | 0 | 0.001322751 |
| chr21 | 40622717 | G | T | BRWD1 | - | intron | silent | 0 | 0.001324503 |
| chr21 | 40627449 | C | T | BRWD1 | - | intron | silent | 0 | 0.001322751 |
| chr21 | 40627482 | C | T | BRWD1 | - | intron | silent | 0 | 0.001321004 |
| chr21 | 40627491 | T | C | BRWD1 | - | intron | silent | 0.001410437 | 0 |
| chr21 | 40627847 | C | T | BRWD1 | - | intron | silent | 0 | 0.001321004 |
| chr21 | 40627872 | T | A | BRWD1 | - | intron | silent | 0.001424501 | 0 |
| chr21 | 40630293 | G | C | BRWD1 | - | intron | silent | 0 | 0.001322751 |
| chr21 | 40630322 | T | C | BRWD1 | - | intron | silent | 0.005641749 | 0.00660502 |
| chr21 | 40630420 | G | A | BRWD1 | - | cds | synonymous | 0 | 0.001321004 |
| chr21 | 40630472 | C | A | BRWD1 | - | cds | missense | 0.001410437 | 0 |
| chr21 | 40630526 | C | A | BRWD1 | - | cds | missense | 0.001410437 | 0.001321004 |
| chr21 | 40630657 | C | T | BRWD1 | - | intron | silent | 0.084626234 | 0.097754293 |
| chr21 | 40630658 | G | A | BRWD1 | - | intron | silent | 0 | 0.002642008 |
| chr21 | 40630736 | C | G | BRWD1 | - | intron | silent | 0.310708899 | 0.279132791 |
| chr21 | 40636248 | A | G | BRWD1 | - | intron | silent | 0.316666667 | 0.271041369 |
| chr21 | 40636351 | C | T | BRWD1 | - | intron | silent | 0.301634473 | 0.276821192 |
| chr21 | 40636503 | G | C | BRWD1 | - | cds | missense | 0 | 0.001321004 |
| chr21 | 40636889 | C | T | BRWD1 | - | cds | synonymous | 0.019746121 | 0.015852048 |
| chr21 | 40636981 | T | C | BRWD1 | - | intron | silent | 0.001410437 | 0 |
| chr21 | 40637036 | A | G | BRWD1 | - | intron | silent | 0 | 0.001331558 |
| chr21 | 40641773 | T | C | BRWD1 | - | intron | silent | 0 | 0.001321004 |
| chr21 | 40641977 | A | G | BRWD1 | - | intron | silent | 0.009873061 | 0.009259259 |
| chr21 | 40642189 | C | T | BRWD1 | - | intron | silent | 0.115655853 | 0.14663144 |
| chr21 | 40642190 | T | C | BRWD1 | - | intron | silent | 0 | 0.001321004 |
| chr21 | 40642217 | T | C | BRWD1 | - | cds | missense | 0 | 0.001321004 |
| chr21 | 40642287 | G | A | BRWD1 | - | cds | synonymous | 0.001410437 | 0.001321004 |
| chr21 | 40642386 | A | G | BRWD1 | - | intron | silent | 0.056417489 | 0.040951123 |
| chr21 | 40642407 | T | C | BRWD1 | - | intron | silent | 0.002820874 | 0 |
| chr21 | 40642421 | T | C | BRWD1 | - | intron | silent | 0.007052186 | 0.001321004 |
| chr21 | 40642524 | G | A | BRWD1 | - | intron | silent | 0 | 0.001459854 |
| chr21 | 40646112 | C | G | BRWD1 | - | intron | silent | 0.004231312 | 0 |
| chr21 | 40646134 | T | C | BRWD1 | - | intron | silent | 0 | 0.00132626 |
| chr21 | 40646144 | G | A | BRWD1 | - | intron | silent | 0.018335684 | 0.014531044 |
| chr21 | 40646168 | T | C | BRWD1 | - | intron | silent | 0 | 0.001322751 |
| chr21 | 40646210 | C | G | BRWD1 | - | intron | silent | 0.042313117 | 0.050198151 |
| chr21 | 40646219 | G | C | BRWD1 | - | intron | silent | 0.001410437 | 0 |
| chr21 | 40646223 | C | G | BRWD1 | - | intron | silent | 0.002820874 | 0.002642008 |
| chr21 | 40646230 | C | A | BRWD1 | - | intron | silent | 0.325811001 | 0.305151915 |
| chr21 | 40646241 | G | A | BRWD1 | - | intron | silent | 0.02538787 | 0.023778071 |
| chr21 | 40646245 | G | A | BRWD1 | - | intron | silent | 0 | 0.001321004 |
| chr21 | 40646268 | T | C | BRWD1 | - | intron | silent | 0 | 0.001321004 |
| chr21 | 40646325 | A | G | BRWD1 | - | cds | synonymous | 0.002820874 | 0.001321004 |
| chr21 | 40646371 | T | C | BRWD1 | - | cds | synonymous | 0 | 0.001321004 |
| chr21 | 40646410 | C | A | BRWD1 | - | intron | silent | 0 | 0.001321004 |
| chr21 | 40646429 | A | T | BRWD1 | - | intron | silent | 0 | 0.001321004 |
| chr21 | 40646593 | G | A | BRWD1 | - | intron | silent | 0.001485884 | 0.004160888 |
| chr21 | 40647946 | G | A | BRWD1 | - | intron | silent | 0.001639344 | 0 |
| chr21 | 40648108 | T | C | BRWD1 | - | cds | missense | 0.001410437 | 0 |
| chr21 | 40648149 | G | T | BRWD1 | - | intron | silent | 0 | 0.001324503 |
| chr21 | 40648186 | C | G | BRWD1 | - | intron | silent | 0.001410437 | 0 |
| chr21 | 40648241 | C | T | BRWD1 | - | intron | silent | 0.341359773 | 0.341333333 |
| chr21 | 40648265 | G | A | BRWD1 | - | intron | silent | 0.13940256 | 0.161073826 |
| chr21 | 40648266 | C | A | BRWD1 | - | intron | silent | 0 | 0.001342282 |
| chr21 | 40649307 | C | A | BRWD1 | - | intron | silent | 0 | 0.001321004 |
| chr21 | 40649310 | G | T | BRWD1 | - | intron | silent | 0.001410437 | 0 |
| chr21 | 40649313 | T | C | BRWD1 | - | intron | silent | 0.019746121 | 0.017173052 |
| chr21 | 40649352 | A | C | BRWD1 | - | intron | silent | 0.001410437 | 0.001324503 |
| chr21 | 40649474 | C | A | BRWD1 | - | intron | silent | 0 | 0.001592357 |
| chr21 | 40650532 | A | G | BRWD1 | - | intron | silent | 0.001412429 | 0 |
| chr21 | 40650550 | T | C | BRWD1 | - | intron | silent | 0.001410437 | 0 |
| chr21 | 40650567 | G | C | BRWD1 | - | intron | silent | 0.308885755 | 0.278731836 |
| chr21 | 40650593 | T | A | BRWD1 | - | intron | silent | 0.001410437 | 0 |
| chr21 | 40650621 | G | A | BRWD1 | - | intron | silent | 0.115655853 | 0.14663144 |
| chr21 | 40650643 | A | T | BRWD1 | - | intron | silent | 0.005641749 | 0.002642008 |
| chr21 | 40650718 | A | G | BRWD1 | - | cds | synonymous | 0.001410437 | 0.001321004 |
| chr21 | 40650774 | T | A | BRWD1 | - | intron | silent | 0.001410437 | 0 |
| chr21 | 40651886 | G | T | BRWD1 | - | intron | silent | 0.001492537 | 0.001342282 |
| chr21 | 40651984 | A | C | BRWD1 | - | intron | silent | 0.001410437 | 0 |
| chr21 | 40652040 | A | C | BRWD1 | - | intron | silent | 0.138222849 | 0.161162483 |
| chr21 | 40652066 | C | A | BRWD1 | - | intron | silent | 0 | 0.001321004 |
| chr21 | 40652142 | G | A | BRWD1 | - | cds | synonymous | 0.056417489 | 0.040951123 |
| chr21 | 40652166 | C | A | BRWD1 | - | cds | synonymous | 0 | 0.001321004 |
| chr21 | 40652234 | T | C | BRWD1 | - | intron | silent | 0 | 0.001321004 |
| chr21 | 40665700 | T | C | BRWD1 | - | intron | silent | 0 | 0.001321004 |
| chr21 | 40665834 | G | A | BRWD1 | - | cds | missense | 0 | 0.001321004 |
| chr21 | 40665921 | T | C | BRWD1 | - | cds | missense | 0 | 0.001321004 |
| chr21 | 40665973 | A | T | BRWD1 | - | intron | silent | 0.001410437 | 0 |
| chr21 | 40666066 | T | C | BRWD1 | - | intron | silent | 0.067335244 | 0.054304636 |
| chr21 | 40666071 | T | C | BRWD1 | - | intron | silent | 0 | 0.00132626 |
| chr21 | 40667610 | T | C | BRWD1 | - | intron | silent | 0.001410437 | 0 |
| chr21 | 40667659 | T | G | BRWD1 | - | intron | silent | 0 | 0.001321004 |
| chr21 | 40667756 | C | A | BRWD1 | - | cds | missense | 0 | 0.001321004 |
| chr21 | 40668112 | T | C | BRWD1 | - | intron | silent | 0.001410437 | 0 |
| chr21 | 40668210 | A | G | BRWD1 | - | cds | synonymous | 0.001410437 | 0 |
| chr21 | 40668326 | A | C | BRWD1 | - | intron | silent | 0 | 0.001321004 |
| chr21 | 40670167 | T | A | BRWD1 | - | utr3 | silent | 0.315517241 | 0.281292059 |
| chr21 | 40670227 | G | A | BRWD1 | - | utr3 | silent | 0.001432665 | 0 |
| chr21 | 40670460 | G | C | BRWD1 | - | cds | missense | 0.304654443 | 0.301188904 |
| chr21 | 40670548 | T | A | BRWD1 | - | intron | silent | 0.001410437 | 0 |
| chr21 | 40670573 | G | T | BRWD1 | - | intron | silent | 0.001412429 | 0.002642008 |
| chr21 | 40684725 | G | A | BRWD1 | - | intron | silent | 0.012295082 | 0 |
| chr21 | 40684806 | G | T | BRWD1 | - | cds | missense | 0 | 0.00621118 |
| chr21 | 40684828 | T | C | BRWD1 | - | intron | silent | 0.005319149 | 0.006369427 |
| chr21 | 40685125 | T | C | BRWD1 | - | intron | silent | 0.011235955 | 0 |
| chr21 | 40685239 | A | G | BRWD1 | - | intron | silent | 0.171764706 | 0.245762712 |
| chr21 | 40685421 | C | G | BRWD1 | - | utr5 | silent | 0.028985507 | 0 |
| chr4 | 157684048 | C | T | PDGFC | - | utr3 | silent | 0 | 0.001322751 |
| chr4 | 157684170 | C | T | PDGFC | - | utr3 | silent | 0.001410437 | 0 |
| chr4 | 157684171 | G | A | PDGFC | - | utr3 | silent | 0.001410437 | 0 |
| chr4 | 157684248 | T | C | PDGFC | - | cds | synonymous | 0.338504937 | 0.252311757 |
| chr4 | 157684424 | C | T | PDGFC | - | intron | silent | 0.001410437 | 0 |
| chr4 | 157688797 | T | G | PDGFC | - | intron | silent | 0 | 0.001322751 |
| chr4 | 157688829 | C | T | PDGFC | - | intron | silent | 0 | 0.003963012 |
| chr4 | 157688894 | A | G | PDGFC | - | intron | silent | 0 | 0.001321004 |
| chr4 | 157689005 | C | T | PDGFC | - | cds | missense | 0 | 0.001321004 |
| chr4 | 157689195 | G | A | PDGFC | - | intron | silent | 0 | 0.001321004 |
| chr4 | 157689235 | G | A | PDGFC | - | intron | silent | 0 | 0.001321004 |
| chr4 | 157693764 | G | A | PDGFC | - | intron | silent | 0.001410437 | 0 |
| chr4 | 157693787 | A | G | PDGFC | - | intron | silent | 0.001410437 | 0 |
| chr4 | 157693877 | G | A | PDGFC | - | cds | missense | 0 | 0.001321004 |
| chr4 | 157694171 | A | G | PDGFC | - | intron | silent | 0.001449275 | 0 |
| chr4 | 157694175 | G | A | PDGFC | - | intron | silent | 0 | 0.002688172 |
| chr4 | 157694207 | T | C | PDGFC | - | intron | silent | 0.001712329 | 0 |
| chr4 | 157731794 | T | C | PDGFC | - | intron | silent | 0.001672241 | 0 |
| chr4 | 157731889 | T | C | PDGFC | - | intron | silent | 0 | 0.001322751 |
| chr4 | 157731906 | G | T | PDGFC | - | intron | silent | 0.001410437 | 0 |
| chr4 | 157731990 | G | A | PDGFC | - | cds | missense | 0 | 0.001321004 |
| chr4 | 157732293 | C | T | PDGFC | - | intron | silent | 0 | 0.001324503 |
| chr4 | 157771237 | A | G | PDGFC | - | intron | silent | 0 | 0.001324503 |
| chr4 | 157771274 | A | T | PDGFC | - | intron | silent | 0.001412429 | 0 |
| chr4 | 157771340 | T | G | PDGFC | - | intron | silent | 0.004231312 | 0.009247028 |
| chr4 | 157771359 | G | A | PDGFC | - | intron | silent | 0 | 0.001321004 |
| chr4 | 157891963 | C | A | PDGFC | - | cds | missense | 0.007052186 | 0.021136063 |
| chr4 | 157892071 | G | T | PDGFC | - | utr5 | silent | 0 | 0.001321004 |
| chr22 | 39917363 | C | T | ATF4 | + | utr5 | silent | 0.003278689 | 0 |
| chr22 | 39917453 | G | A | ATF4 | + | cds | missense | 0 | 0.001321004 |
| chr22 | 39917457 | G | A | ATF4 | + | cds | missense | 0.02970297 | 0.018494055 |
| chr22 | 39917515 | A | C | ATF4 | + | cds | missense | 0.354019746 | 0.340819022 |
| chr22 | 39917549 | C | T | ATF4 | + | cds | synonymous | 0 | 0.001321004 |
| chr22 | 39917591 | T | G | ATF4 | + | cds | missense | 0 | 0.001321004 |
| chr22 | 39917611 | C | G | ATF4 | + | cds | missense | 0.001410437 | 0 |
| chr22 | 39917666 | C | T | ATF4 | + | cds | synonymous | 0.002820874 | 0.001321004 |
| chr22 | 39917725 | C | T | ATF4 | + | intron | silent | 0.001412429 | 0.00660502 |
| chr22 | 39917753 | G | T | ATF4 | + | intron | silent | 0.029619182 | 0.018494055 |
| chr22 | 39917805 | T | A | ATF4 | + | cds | missense | 0 | 0.001321004 |
| chr22 | 39917919 | T | G | ATF4 | + | cds | missense | 0 | 0.001321004 |
| chr22 | 39917941 | T | G | ATF4 | + | cds | synonymous | 0 | 0.001321004 |
| chr22 | 39918003 | C | T | ATF4 | + | cds | missense | 0.001436782 | 0 |
| chr22 | 39918036 | C | T | ATF4 | + | cds | missense | 0.004279601 | 0.014531044 |
| chr22 | 39918046 | T | C | ATF4 | + | cds | synonymous | 0 | 0.001321004 |
| chr22 | 39918056 | G | C | ATF4 | + | cds | missense | 0 | 0.001321004 |
| chr22 | 39918063 | C | T | ATF4 | + | cds | missense | 0 | 0.001321004 |
| chr22 | 39918099 | G | C | ATF4 | + | cds | missense | 0 | 0.001321004 |
| chr22 | 39918127 | T | C | ATF4 | + | cds | synonymous | 0.001420455 | 0 |
| chr22 | 39918236 | C | T | ATF4 | + | cds | synonymous | 0.001410437 | 0 |
| chr22 | 39918358 | A | G | ATF4 | + | cds | synonymous | 0.001410437 | 0 |
| chr22 | 39918373 | G | A | ATF4 | + | cds | synonymous | 0.001412429 | 0 |
| chr22 | 39918429 | C | T | ATF4 | + | cds | missense | 0 | 0.001321004 |
| chr22 | 39918544 | C | G | ATF4 | + | cds | missense | 0.001436782 | 0.001321004 |
| chr22 | 39918688 | A | G | ATF4 | + | utr3 | silent | 0.002016129 | 0.001416431 |
| chr22 | 31608387 | C | A | LIMK2 | + | utr5 | silent | 0.002824859 | 0.001322751 |
| chr22 | 31608475 | G | A | LIMK2 | + | intron | silent | 0 | 0.001449275 |
| chr22 | 31608496 | G | T | LIMK2 | + | intron | silent | 0.001506024 | 0.001540832 |
| chr22 | 31621698 | G | A | LIMK2 | + | intron | silent | 0.001410437 | 0.001321004 |
| chr22 | 31621792 | G | A | LIMK2 | + | cds | missense | 0.04090268 | 0.043593131 |
| chr22 | 31621828 | C | A | LIMK2 | + | intron | silent | 0 | 0.001321004 |
| chr22 | 31621863 | T | C | LIMK2 | + | intron | silent | 0.001410437 | 0 |
| chr22 | 31621868 | T | C | LIMK2 | + | intron | silent | 0 | 0.001321004 |
| chr22 | 31621983 | A | G | LIMK2 | + | intron | silent | 0.007598784 | 0.011006289 |
| chr22 | 31644614 | C | T | LIMK2 | + | utr5 | silent | 0.040960452 | 0.042328042 |
| chr22 | 31644618 | C | G | LIMK2 | + | utr5 | silent | 0 | 0.001322751 |
| chr22 | 31644648 | G | A | LIMK2 | + | utr5 | silent | 0.001410437 | 0 |
| chr22 | 31644725 | C | T | LIMK2 | + | cds | missense | 0.001410437 | 0 |
| chr22 | 31644736 | A | G | LIMK2 | + | cds | missense | 0 | 0.001321004 |
| chr22 | 31644856 | C | T | LIMK2 | + | intron | silent | 0 | 0.001322751 |
| chr22 | 31644866 | G | A | LIMK2 | + | intron | silent | 0.001410437 | 0.005284016 |
| chr22 | 31654181 | C | T | LIMK2 | + | intron | silent | 0 | 0.001321004 |
| chr22 | 31654184 | T | A | LIMK2 | + | intron | silent | 0 | 0.001321004 |
| chr22 | 31654293 | G | A | LIMK2 | + | cds | missense | 0.004231312 | 0.00660502 |
| chr22 | 31654523 | T | A | LIMK2 | + | intron | silent | 0 | 0.001321004 |
| chr22 | 31654562 | T | C | LIMK2 | + | intron | silent | 0 | 0.001336898 |
| chr22 | 31654998 | A | G | LIMK2 | + | intron | silent | 0.001410437 | 0.001321004 |
| chr22 | 31654999 | C | T | LIMK2 | + | intron | silent | 0.001410437 | 0 |
| chr22 | 31655186 | A | T | LIMK2 | + | cds | missense | 0 | 0.002642008 |
| chr22 | 31655208 | C | T | LIMK2 | + | cds | synonymous | 0.02538787 | 0.017173052 |
| chr22 | 31655363 | A | G | LIMK2 | + | intron | silent | 0 | 0.001329787 |
| chr22 | 31656176 | T | C | LIMK2 | + | intron | silent | 0 | 0.001329787 |
| chr22 | 31657959 | T | C | LIMK2 | + | intron | silent | 0 | 0.003963012 |
| chr22 | 31657968 | C | T | LIMK2 | + | intron | silent | 0.001414427 | 0.001321004 |
| chr22 | 31658186 | G | A | LIMK2 | + | cds | synonymous | 0.001410437 | 0 |
| chr22 | 31658202 | G | A | LIMK2 | + | cds | missense | 0 | 0.001321004 |
| chr22 | 31658205 | C | T | LIMK2 | + | cds | missense | 0.001410437 | 0.011889036 |
| chr22 | 31658281 | A | G | LIMK2 | + | intron | silent | 0.018335684 | 0.025099075 |
| chr22 | 31658313 | C | G | LIMK2 | + | intron | silent | 0 | 0.001321004 |
| chr22 | 31658371 | G | T | LIMK2 | + | intron | silent | 0.001410437 | 0 |
| chr22 | 31658373 | G | A | LIMK2 | + | intron | silent | 0.001410437 | 0 |
| chr22 | 31658484 | T | C | LIMK2 | + | intron | silent | 0 | 0.001321004 |
| chr22 | 31658693 | T | C | LIMK2 | + | cds | missense | 0.001410437 | 0 |
| chr22 | 31658715 | C | T | LIMK2 | + | cds | synonymous | 0 | 0.001321004 |
| chr22 | 31658801 | G | A | LIMK2 | + | intron | silent | 0 | 0.001321004 |
| chr22 | 31658814 | T | C | LIMK2 | + | intron | silent | 0.001410437 | 0.002642008 |
| chr22 | 31658824 | G | A | LIMK2 | + | intron | silent | 0.001410437 | 0 |
| chr22 | 31658883 | G | A | LIMK2 | + | intron | silent | 0.013215859 | 0.008287293 |
| chr22 | 31661735 | T | C | LIMK2 | + | intron | silent | 0.001733102 | 0 |
| chr22 | 31661798 | C | T | LIMK2 | + | intron | silent | 0.002828854 | 0 |
| chr22 | 31661834 | T | G | LIMK2 | + | intron | silent | 0.001410437 | 0 |
| chr22 | 31661964 | C | G | LIMK2 | + | cds | missense | 0.001410437 | 0 |
| chr22 | 31662010 | C | T | LIMK2 | + | cds | synonymous | 0.021156559 | 0.021136063 |
| chr22 | 31662148 | T | C | LIMK2 | + | intron | silent | 0.001410437 | 0.001321004 |
| chr22 | 31662177 | T | C | LIMK2 | + | intron | silent | 0.001410437 | 0.001321004 |
| chr22 | 31662178 | C | T | LIMK2 | + | intron | silent | 0.001410437 | 0.001321004 |
| chr22 | 31662860 | G | A | LIMK2 | + | intron | silent | 0.001410437 | 0.001321004 |
| chr22 | 31662895 | C | A | LIMK2 | + | intron | silent | 0.001410437 | 0 |
| chr22 | 31663089 | G | A | LIMK2 | + | intron | silent | 0 | 0.001321004 |
| chr22 | 31663104 | G | A | LIMK2 | + | intron | silent | 0.009873061 | 0.010568032 |
| chr22 | 31663170 | G | A | LIMK2 | + | intron | silent | 0 | 0.001321004 |
| chr22 | 31663575 | A | G | LIMK2 | + | intron | silent | 0 | 0.003021148 |
| chr22 | 31663651 | A | C | LIMK2 | + | intron | silent | 0.001416431 | 0 |
| chr22 | 31663669 | G | T | LIMK2 | + | intron | silent | 0 | 0.001321004 |
| chr22 | 31663692 | T | G | LIMK2 | + | intron | silent | 0 | 0.001321004 |
| chr22 | 31663758 | C | T | LIMK2 | + | intron | silent | 0.001410437 | 0 |
| chr22 | 31663842 | C | G | LIMK2 | + | cds | synonymous | 0.018335684 | 0.025099075 |
| chr22 | 31663885 | C | T | LIMK2 | + | cds | missense | 0 | 0.003963012 |
| chr22 | 31663938 | C | T | LIMK2 | + | intron | silent | 0 | 0.001321004 |
| chr22 | 31663983 | G | A | LIMK2 | + | intron | silent | 0.001410437 | 0 |
| chr22 | 31663985 | C | T | LIMK2 | + | intron | silent | 0.383638928 | 0.379128137 |
| chr22 | 31664032 | C | T | LIMK2 | + | intron | silent | 0.143864598 | 0.169088507 |
| chr22 | 31664119 | C | T | LIMK2 | + | intron | silent | 0.001410437 | 0.001321004 |
| chr22 | 31664135 | G | A | LIMK2 | + | cds | synonymous | 0.001410437 | 0 |
| chr22 | 31664245 | T | A | LIMK2 | + | intron | silent | 0 | 0.001321004 |
| chr22 | 31664268 | C | T | LIMK2 | + | intron | silent | 0.001410437 | 0 |
| chr22 | 31664276 | T | G | LIMK2 | + | intron | silent | 0.001412429 | 0 |
| chr22 | 31664278 | T | A | LIMK2 | + | intron | silent | 0.001412429 | 0 |
| chr22 | 31664288 | G | A | LIMK2 | + | intron | silent | 0.001414427 | 0.001321004 |
| chr22 | 31664312 | T | G | LIMK2 | + | intron | silent | 0.008486563 | 0.003963012 |
| chr22 | 31666998 | C | G | LIMK2 | + | intron | silent | 0.001410437 | 0 |
| chr22 | 31667007 | G | A | LIMK2 | + | intron | silent | 0.001410437 | 0 |
| chr22 | 31667301 | G | C | LIMK2 | + | intron | silent | 0.001410437 | 0 |
| chr22 | 31667307 | A | G | LIMK2 | + | intron | silent | 0 | 0.001322751 |
| chr22 | 31667358 | C | T | LIMK2 | + | intron | silent | 0.001414427 | 0 |
| chr22 | 31668392 | C | G | LIMK2 | + | intron | silent | 0.00141844 | 0 |
| chr22 | 31668480 | C | T | LIMK2 | + | intron | silent | 0 | 0.001321004 |
| chr22 | 31668632 | C | T | LIMK2 | + | cds | synonymous | 0 | 0.001321004 |
| chr22 | 31668794 | C | T | LIMK2 | + | intron | silent | 0.039716312 | 0.054376658 |
| chr22 | 31668803 | A | G | LIMK2 | + | intron | silent | 0.002881844 | 0.001336898 |
| chr22 | 31669331 | A | T | LIMK2 | + | intron | silent | 0 | 0.001321004 |
| chr22 | 31669368 | T | A | LIMK2 | + | intron | silent | 0.001410437 | 0.001321004 |
| chr22 | 31669384 | G | A | LIMK2 | + | intron | silent | 0 | 0.001321004 |
| chr22 | 31669401 | T | C | LIMK2 | + | intron | silent | 0.001410437 | 0 |
| chr22 | 31669481 | C | T | LIMK2 | + | cds | synonymous | 0.001410437 | 0 |
| chr22 | 31669554 | G | A | LIMK2 | + | intron | silent | 0.001410437 | 0 |
| chr22 | 31669608 | T | C | LIMK2 | + | intron | silent | 0.001410437 | 0 |
| chr22 | 31669652 | G | A | LIMK2 | + | intron | silent | 0.001412429 | 0 |
| chr22 | 31669681 | A | G | LIMK2 | + | intron | silent | 0.001432665 | 0 |
| chr22 | 31671123 | T | G | LIMK2 | + | intron | silent | 0.080394922 | 0.075297226 |
| chr22 | 31671233 | G | A | LIMK2 | + | cds | missense | 0.001410437 | 0.002642008 |
| chr22 | 31672752 | A | C | LIMK2 | + | intron | silent | 0.023300971 | 0.019230769 |
| chr22 | 31672761 | G | C | LIMK2 | + | intron | silent | 0.037478705 | 0.028328612 |
| chr22 | 31672933 | A | G | LIMK2 | + | cds | missense | 0.002820874 | 0.003963012 |
| chr22 | 31673111 | A | G | LIMK2 | + | cds | synonymous | 0.380681818 | 0.37962963 |
| chr22 | 31673116 | A | G | LIMK2 | + | cds | missense | 0.380681818 | 0.37962963 |
| chr22 | 31673119 | A | G | LIMK2 | + | cds | missense | 0 | 0.001322751 |
| chr22 | 31673159 | T | C | LIMK2 | + | utr3 | silent | 0.00152207 | 0 |
| chr22 | 31673185 | G | A | LIMK2 | + | utr3 | silent | 0.001792115 | 0 |
| chr22 | 31673201 | C | T | LIMK2 | + | utr3 | silent | 0.001960784 | 0.003333333 |
| chr22 | 31674172 | A | C | LIMK2 | + | intron | silent | 0.001410437 | 0 |
| chr22 | 31674200 | G | A | LIMK2 | + | intron | silent | 0.385049365 | 0.379128137 |
| chr22 | 31674280 | T | C | LIMK2 | + | intron | silent | 0 | 0.001321004 |
| chr22 | 31674324 | C | G | LIMK2 | + | cds | missense | 0 | 0.002642008 |
| chr22 | 31674435 | C | T | LIMK2 | + | utr3 | silent | 0 | 0.001321004 |
| chr22 | 31674507 | C | T | LIMK2 | + | utr3 | silent | 0.001414427 | 0 |
| chr22 | 31674511 | C | T | LIMK2 | + | utr3 | silent | 0 | 0.001328021 |
| chr22 | 31674512 | G | C | LIMK2 | + | utr3 | silent | 0.001416431 | 0 |
| chr22 | 31674532 | G | A | LIMK2 | + | utr3 | silent | 0 | 0.001381215 |
| chr22 | 31674553 | G | A | LIMK2 | + | utr3 | silent | 0.379008746 | 0.365682138 |
| chr22 | 31674610 | C | T | LIMK2 | + | utr3 | silent | 0.001644737 | 0.001697793 |
| chr1 | 203097932 | T | G | ADORA1 | + | utr5 | silent | 0.019858156 | 0.009320905 |
| chr1 | 203097965 | C | G | ADORA1 | + | utr5 | silent | 0.001410437 | 0 |
| chr1 | 203097976 | C | T | ADORA1 | + | cds | missense | 0.002820874 | 0.00660502 |
| chr1 | 203098119 | G | T | ADORA1 | + | cds | synonymous | 0.001410437 | 0 |
| chr1 | 203098139 | C | T | ADORA1 | + | cds | missense | 0 | 0.001321004 |
| chr1 | 203098185 | G | A | ADORA1 | + | cds | synonymous | 0.001410437 | 0 |
| chr1 | 203098275 | T | G | ADORA1 | + | cds | synonymous | 0.307475317 | 0.260237781 |
| chr1 | 203098336 | C | T | ADORA1 | + | intron | silent | 0.002824859 | 0.005284016 |
| chr1 | 203098399 | C | A | ADORA1 | + | intron | silent | 0.001517451 | 0 |
| chr1 | 203098455 | T | C | ADORA1 | + | intron | silent | 0.329639889 | 0.287206266 |
| chr1 | 203134282 | G | A | ADORA1 | + | intron | silent | 0.001538462 | 0 |
| chr1 | 203134320 | C | T | ADORA1 | + | intron | silent | 0.001445087 | 0 |
| chr1 | 203134321 | G | A | ADORA1 | + | intron | silent | 0.004341534 | 0.004149378 |
| chr1 | 203134366 | C | T | ADORA1 | + | intron | silent | 0.016949153 | 0.015957447 |
| chr1 | 203134499 | C | T | ADORA1 | + | cds | missense | 0 | 0.001321004 |
| chr1 | 203134529 | G | C | ADORA1 | + | cds | missense | 0 | 0.003963012 |
| chr1 | 203134623 | G | A | ADORA1 | + | cds | synonymous | 0.001410437 | 0.001321004 |
| chr1 | 203134637 | T | C | ADORA1 | + | cds | missense | 0 | 0.001321004 |
| chr1 | 203134648 | C | G | ADORA1 | + | cds | missense | 0 | 0.001321004 |
| chr1 | 203134713 | G | A | ADORA1 | + | cds | synonymous | 0.001410437 | 0 |
| chr1 | 203134829 | C | T | ADORA1 | + | cds | missense | 0 | 0.001321004 |
| chr1 | 203134859 | A | C | ADORA1 | + | cds | missense | 0.001410437 | 0 |
| chr1 | 203135001 | T | C | ADORA1 | + | cds | synonymous | 0 | 0.002642008 |
| chr1 | 203135030 | C | A | ADORA1 | + | utr3 | silent | 0.001410437 | 0.00132626 |
| chr1 | 203135145 | A | G | ADORA1 | + | utr3 | silent | 0 | 0.002227171 |
| chr1 | 20915490 | C | T | CDA | + | utr5 | silent | 0 | 0.001324503 |
| chr1 | 20915507 | G | A | CDA | + | utr5 | silent | 0.122708039 | 0.115079365 |
| chr1 | 20915531 | A | G | CDA | + | utr5 | silent | 0.227080395 | 0.224570674 |
| chr1 | 20915576 | C | T | CDA | + | utr5 | silent | 0 | 0.001321004 |
| chr1 | 20915580 | G | C | CDA | + | utr5 | silent | 0.001410437 | 0.001321004 |
| chr1 | 20915590 | C | T | CDA | + | utr5 | silent | 0.091666667 | 0.056478405 |
| chr1 | 20915624 | T | C | CDA | + | cds | missense | 0 | 0.001321004 |
| chr1 | 20915701 | A | C | CDA | + | cds | missense | 0.225669958 | 0.221928666 |
| chr1 | 20915738 | G | C | CDA | + | cds | missense | 0.001410437 | 0.001321004 |
| chr1 | 20915813 | G | A | CDA | + | intron | silent | 0.383638928 | 0.39602649 |
| chr1 | 20915909 | G | A | CDA | + | intron | silent | 0.001610306 | 0 |
| chr1 | 20931237 | T | C | CDA | + | intron | silent | 0.001432665 | 0 |
| chr1 | 20931273 | G | A | CDA | + | intron | silent | 0 | 0.001321004 |
| chr1 | 20931279 | C | T | CDA | + | intron | silent | 0 | 0.001321004 |
| chr1 | 20931474 | G | A | CDA | + | cds | missense | 0.001410437 | 0.002642008 |
| chr1 | 20931476 | T | C | CDA | + | cds | synonymous | 0.001410437 | 0.001321004 |
| chr1 | 20931481 | A | G | CDA | + | cds | missense | 0 | 0.002642008 |
| chr1 | 20931489 | G | A | CDA | + | cds | missense | 0.001410437 | 0 |
| chr1 | 20931578 | G | T | CDA | + | intron | silent | 0.001410437 | 0 |
| chr1 | 20931619 | T | C | CDA | + | intron | silent | 0.001410437 | 0 |
| chr1 | 20940224 | G | A | CDA | + | intron | silent | 0 | 0.001321004 |
| chr1 | 20940330 | C | T | CDA | + | intron | silent | 0 | 0.001321004 |
| chr1 | 20940344 | A | C | CDA | + | cds | missense | 0 | 0.00660502 |
| chr1 | 20940363 | T | C | CDA | + | cds | missense | 0 | 0.001321004 |
| chr1 | 20940385 | T | C | CDA | + | cds | missense | 0.001410437 | 0 |
| chr1 | 20940463 | T | C | CDA | + | intron | silent | 0.152327221 | 0.170409511 |
| chr1 | 20944820 | T | A | CDA | + | intron | silent | 0 | 0.001321004 |
| chr1 | 20944889 | G | A | CDA | + | intron | silent | 0.430183357 | 0.434610304 |
| chr1 | 20944909 | G | A | CDA | + | intron | silent | 0.430183357 | 0.4332893 |
| chr1 | 20944922 | C | T | CDA | + | intron | silent | 0.430183357 | 0.4332893 |
| chr1 | 20945003 | C | T | CDA | + | cds | missense | 0 | 0.002642008 |
| chr1 | 20945046 | G | C | CDA | + | cds | synonymous | 0.001410437 | 0 |
| chr1 | 20945055 | C | T | CDA | + | cds | synonymous | 0.430183357 | 0.4332893 |
| chr22 | 19118805 | C | A | TSSK2 | + | utr5 | silent | 0.00141844 | 0 |
| chr22 | 19118863 | C | T | TSSK2 | + | utr5 | silent | 0 | 0.001321004 |
| chr22 | 19118901 | G | A | TSSK2 | + | utr5 | silent | 0.009873061 | 0.025099075 |
| chr22 | 19118918 | C | T | TSSK2 | + | cds | synonymous | 0.001410437 | 0 |
| chr22 | 19118951 | C | T | TSSK2 | + | cds | synonymous | 0 | 0.001321004 |
| chr22 | 19118981 | C | T | TSSK2 | + | cds | synonymous | 0 | 0.001321004 |
| chr22 | 19118992 | A | G | TSSK2 | + | cds | missense | 0.042313117 | 0.029062087 |
| chr22 | 19119093 | A | G | TSSK2 | + | cds | missense | 0.002820874 | 0.003963012 |
| chr22 | 19119116 | C | T | TSSK2 | + | cds | synonymous | 0.001410437 | 0 |
| chr22 | 19119120 | G | A | TSSK2 | + | cds | missense | 0.001410437 | 0 |
| chr22 | 19119146 | C | A | TSSK2 | + | cds | synonymous | 0.001410437 | 0.002642008 |
| chr22 | 19119161 | C | T | TSSK2 | + | cds | synonymous | 0.019746121 | 0.017173052 |
| chr22 | 19119192 | G | A | TSSK2 | + | cds | missense | 0 | 0.001321004 |
| chr22 | 19119245 | C | T | TSSK2 | + | cds | synonymous | 0.004231312 | 0.003963012 |
| chr22 | 19119247 | T | G | TSSK2 | + | cds | missense | 0 | 0.001321004 |
| chr22 | 19119278 | C | T | TSSK2 | + | cds | synonymous | 0 | 0.001321004 |
| chr22 | 19119391 | G | A | TSSK2 | + | cds | missense | 0 | 0.001321004 |
| chr22 | 19119399 | C | T | TSSK2 | + | cds | missense | 0 | 0.001321004 |
| chr22 | 19119490 | A | C | TSSK2 | + | cds | missense | 0 | 0.001321004 |
| chr22 | 19119502 | A | G | TSSK2 | + | cds | missense | 0.055007052 | 0.055482166 |
| chr22 | 19119522 | G | A | TSSK2 | + | cds | missense | 0 | 0.002642008 |
| chr22 | 19119524 | G | A | TSSK2 | + | cds | synonymous | 0 | 0.001321004 |
| chr22 | 19119545 | C | T | TSSK2 | + | cds | synonymous | 0.251057828 | 0.252311757 |
| chr22 | 19119559 | A | G | TSSK2 | + | cds | missense | 0 | 0.001321004 |
| chr22 | 19119589 | G | A | TSSK2 | + | cds | missense | 0.001410437 | 0 |
| chr22 | 19119597 | A | G | TSSK2 | + | cds | missense | 0.001410437 | 0 |
| chr22 | 19119686 | C | T | TSSK2 | + | cds | synonymous | 0.251057828 | 0.252311757 |
| chr22 | 19119702 | G | A | TSSK2 | + | cds | missense | 0.001412429 | 0 |
| chr22 | 19119732 | C | G | TSSK2 | + | cds | missense | 0 | 0.001321004 |
| chr22 | 19119743 | C | G | TSSK2 | + | cds | synonymous | 0 | 0.001321004 |
| chr22 | 19119751 | C | T | TSSK2 | + | cds | missense | 0.36248237 | 0.351387054 |
| chr22 | 19119833 | C | T | TSSK2 | + | cds | synonymous | 0 | 0.001321004 |
| chr22 | 19119841 | G | A | TSSK2 | + | cds | missense | 0 | 0.003968254 |
| chr22 | 19119861 | G | C | TSSK2 | + | cds | missense | 0 | 0.001324503 |
| chr22 | 19119900 | G | A | TSSK2 | + | cds | missense | 0 | 0.001322751 |
| chr22 | 19119927 | G | A | TSSK2 | + | cds | missense | 0 | 0.00132626 |
| chr22 | 19119938 | G | A | TSSK2 | + | cds | synonymous | 0.289139633 | 0.277410832 |
| chr22 | 19120005 | G | A | TSSK2 | + | utr3 | silent | 0.027737226 | 0.022068966 |
| chr22 | 19120047 | C | T | TSSK2 | + | utr3 | silent | 0.012718601 | 0.021148036 |
| chr22 | 19120110 | C | T | TSSK2 | + | utr3 | silent | 0.005194805 | 0 |
| chr6 | 42531966 | C | T | UBR2 | + | utr5 | silent | 0 | 0.00140056 |
| chr6 | 42531995 | G | C | UBR2 | + | utr5 | silent | 0.00443787 | 0 |
| chr6 | 42532102 | C | T | UBR2 | + | cds | synonymous | 0.325811001 | 0.363756614 |
| chr6 | 42532120 | C | T | UBR2 | + | cds | synonymous | 0.002820874 | 0 |
| chr6 | 42532182 | G | C | UBR2 | + | intron | silent | 0 | 0.001408451 |
| chr6 | 42541459 | T | C | UBR2 | + | intron | silent | 0.002820874 | 0.00660502 |
| chr6 | 42541802 | G | C | UBR2 | + | intron | silent | 0.196605375 | 0.20343461 |
| chr6 | 42559863 | T | C | UBR2 | + | intron | silent | 0 | 0.001321004 |
| chr6 | 42559864 | T | C | UBR2 | + | intron | silent | 0.001410437 | 0 |
| chr6 | 42559926 | G | A | UBR2 | + | cds | missense | 0.001410437 | 0.002642008 |
| chr6 | 42559928 | G | C | UBR2 | + | cds | missense | 0 | 0.001321004 |
| chr6 | 42559964 | T | C | UBR2 | + | cds | synonymous | 0.001410437 | 0 |
| chr6 | 42559995 | C | T | UBR2 | + | intron | silent | 0 | 0.001321004 |
| chr6 | 42560022 | C | A | UBR2 | + | intron | silent | 0.001410437 | 0 |
| chr6 | 42560064 | G | A | UBR2 | + | intron | silent | 0.002820874 | 0 |
| chr6 | 42562021 | C | A | UBR2 | + | cds | synonymous | 0.002820874 | 0.001321004 |
| chr6 | 42562027 | A | T | UBR2 | + | cds | missense | 0.011283498 | 0.021136063 |
| chr6 | 42562098 | T | C | UBR2 | + | intron | silent | 0 | 0.001321004 |
| chr6 | 42562107 | G | A | UBR2 | + | intron | silent | 0.001410437 | 0 |
| chr6 | 42562199 | C | T | UBR2 | + | intron | silent | 0.001428571 | 0.001333333 |
| chr6 | 42562227 | T | C | UBR2 | + | intron | silent | 0.012158055 | 0.022191401 |
| chr6 | 42562238 | A | G | UBR2 | + | intron | silent | 0.012820513 | 0.022824536 |
| chr6 | 42571127 | A | G | UBR2 | + | intron | silent | 0.001628664 | 0 |
| chr6 | 42571279 | T | G | UBR2 | + | intron | silent | 0.138222849 | 0.162913907 |
| chr6 | 42571338 | C | A | UBR2 | + | cds | missense | 0.004231312 | 0.006622517 |
| chr6 | 42571346 | A | T | UBR2 | + | cds | synonymous | 0.197461213 | 0.202649007 |
| chr6 | 42571467 | C | A | UBR2 | + | intron | silent | 0 | 0.002649007 |
| chr6 | 42571478 | T | C | UBR2 | + | intron | silent | 0 | 0.001324503 |
| chr6 | 42571544 | A | G | UBR2 | + | intron | silent | 0.001420455 | 0 |
| chr6 | 42571566 | A | G | UBR2 | + | intron | silent | 0.001426534 | 0.001329787 |
| chr6 | 42573285 | A | G | UBR2 | + | intron | silent | 0.208695652 | 0.232496697 |
| chr6 | 42573312 | A | G | UBR2 | + | intron | silent | 0 | 0.001321004 |
| chr6 | 42573315 | G | A | UBR2 | + | intron | silent | 0 | 0.001321004 |
| chr6 | 42573422 | C | T | UBR2 | + | intron | silent | 0 | 0.001321004 |
| chr6 | 42573513 | T | C | UBR2 | + | cds | synonymous | 0.005641749 | 0.007926024 |
| chr6 | 42573627 | T | C | UBR2 | + | intron | silent | 0 | 0.001322751 |
| chr6 | 42573712 | A | G | UBR2 | + | intron | silent | 0 | 0.001356852 |
| chr6 | 42574203 | G | A | UBR2 | + | intron | silent | 0.002857143 | 0 |
| chr6 | 42574277 | T | C | UBR2 | + | intron | silent | 0.001412429 | 0 |
| chr6 | 42574283 | T | A | UBR2 | + | intron | silent | 0.001412429 | 0 |
| chr6 | 42574318 | T | C | UBR2 | + | intron | silent | 0 | 0.001322751 |
| chr6 | 42574320 | T | G | UBR2 | + | intron | silent | 0 | 0.001322751 |
| chr6 | 42574450 | T | C | UBR2 | + | intron | silent | 0.001414427 | 0 |
| chr6 | 42579786 | C | A | UBR2 | + | intron | silent | 0 | 0.001324503 |
| chr6 | 42579815 | A | G | UBR2 | + | intron | silent | 0.001412429 | 0 |
| chr6 | 42579859 | A | G | UBR2 | + | intron | silent | 0.001410437 | 0.001321004 |
| chr6 | 42579862 | G | T | UBR2 | + | intron | silent | 0 | 0.001321004 |
| chr6 | 42579917 | C | T | UBR2 | + | cds | synonymous | 0.001410437 | 0 |
| chr6 | 42579918 | A | G | UBR2 | + | cds | missense | 0 | 0.001321004 |
| chr6 | 42579925 | A | G | UBR2 | + | cds | missense | 0.001410437 | 0 |
| chr6 | 42579944 | T | C | UBR2 | + | cds | synonymous | 0 | 0.001321004 |
| chr6 | 42580175 | A | G | UBR2 | + | intron | silent | 0.002894356 | 0.002695418 |
| chr6 | 42580214 | G | T | UBR2 | + | intron | silent | 0 | 0.001477105 |
| chr6 | 42580225 | T | C | UBR2 | + | intron | silent | 0 | 0.00304414 |
| chr6 | 42582824 | T | C | UBR2 | + | cds | missense | 0 | 0.001321004 |
| chr6 | 42582882 | C | T | UBR2 | + | cds | synonymous | 0.002820874 | 0 |
| chr6 | 42582986 | T | C | UBR2 | + | intron | silent | 0.011283498 | 0.021136063 |
| chr6 | 42583578 | C | T | UBR2 | + | intron | silent | 0 | 0.002688172 |
| chr6 | 42583625 | G | A | UBR2 | + | intron | silent | 0.271186441 | 0.258916777 |
| chr6 | 42583670 | A | C | UBR2 | + | intron | silent | 0 | 0.001321004 |
| chr6 | 42583701 | G | A | UBR2 | + | intron | silent | 0 | 0.001321004 |
| chr6 | 42583708 | C | T | UBR2 | + | intron | silent | 0 | 0.001321004 |
| chr6 | 42583910 | C | T | UBR2 | + | intron | silent | 0.002824859 | 0.001324503 |
| chr6 | 42584004 | C | A | UBR2 | + | intron | silent | 0 | 0.001449275 |
| chr6 | 42585019 | C | T | UBR2 | + | cds | synonymous | 0 | 0.001321004 |
| chr6 | 42585022 | G | A | UBR2 | + | cds | synonymous | 0.005641749 | 0.007926024 |
| chr6 | 42585113 | T | C | UBR2 | + | intron | silent | 0 | 0.001321004 |
| chr6 | 42585270 | C | T | UBR2 | + | intron | silent | 0.011283498 | 0.021136063 |
| chr6 | 42600169 | T | C | UBR2 | + | intron | silent | 0.001492537 | 0 |
| chr6 | 42600319 | A | G | UBR2 | + | cds | synonymous | 0 | 0 |
| chr6 | 42600392 | C | T | UBR2 | + | cds | nonsense | 0 | 0.001321004 |
| chr6 | 42600471 | T | C | UBR2 | + | intron | silent | 0.035260931 | 0.050198151 |
| chr6 | 42600499 | T | G | UBR2 | + | intron | silent | 0 | 0.001321004 |
| chr6 | 42600522 | T | G | UBR2 | + | intron | silent | 0 | 0.001321004 |
| chr6 | 42600547 | G | T | UBR2 | + | cds | missense | 0.001410437 | 0 |
| chr6 | 42600649 | A | G | UBR2 | + | intron | silent | 0 | 0.001321004 |
| chr6 | 42600747 | T | A | UBR2 | + | intron | silent | 0 | 0.003738318 |
| chr6 | 42600748 | C | A | UBR2 | + | intron | silent | 0 | 0.003780718 |
| chr6 | 42600749 | A | C | UBR2 | + | intron | silent | 0 | 0.003816794 |
| chr6 | 42602960 | A | G | UBR2 | + | intron | silent | 0 | 0.001404494 |
| chr6 | 42602995 | A | G | UBR2 | + | intron | silent | 0 | 0.001338688 |
| chr6 | 42603050 | A | G | UBR2 | + | intron | silent | 0.001416431 | 0.002649007 |
| chr6 | 42603118 | T | A | UBR2 | + | intron | silent | 0.011283498 | 0.021136063 |
| chr6 | 42603150 | G | A | UBR2 | + | intron | silent | 0.001410437 | 0 |
| chr6 | 42603391 | T | C | UBR2 | + | intron | silent | 0 | 0.002645503 |
| chr6 | 42603406 | C | T | UBR2 | + | intron | silent | 0 | 0.00132626 |
| chr6 | 42604565 | G | A | UBR2 | + | intron | silent | 0.001666667 | 0 |
| chr6 | 42604646 | T | C | UBR2 | + | intron | silent | 0.011299435 | 0.021136063 |
| chr6 | 42604647 | A | C | UBR2 | + | intron | silent | 0.011299435 | 0.021136063 |
| chr6 | 42604688 | T | C | UBR2 | + | intron | silent | 0 | 0.001321004 |
| chr6 | 42604698 | T | C | UBR2 | + | intron | silent | 0.001412429 | 0.001321004 |
| chr6 | 42604763 | C | T | UBR2 | + | cds | synonymous | 0.002820874 | 0 |
| chr6 | 42604784 | C | T | UBR2 | + | cds | synonymous | 0 | 0.001321004 |
| chr6 | 42604917 | T | C | UBR2 | + | cds | missense | 0.002820874 | 0.005284016 |
| chr6 | 42607771 | T | A | UBR2 | + | intron | silent | 0 | 0 |
| chr6 | 42607772 | T | A | UBR2 | + | intron | silent | 0.012618297 | 0.022130014 |
| chr6 | 42607841 | C | T | UBR2 | + | intron | silent | 0.198300283 | 0.206349206 |
| chr6 | 42607887 | A | T | UBR2 | + | intron | silent | 0.001412429 | 0 |
| chr6 | 42607939 | T | A | UBR2 | + | intron | silent | 0 | 0.001324503 |
| chr6 | 42608123 | T | G | UBR2 | + | intron | silent | 0.012965964 | 0.011686144 |
| chr6 | 42608125 | T | G | UBR2 | + | intron | silent | 0.008389262 | 0.008849558 |
| chr6 | 42608126 | G | A | UBR2 | + | intron | silent | 0.003350084 | 0 |
| chr6 | 42608129 | T | G | UBR2 | + | intron | silent | 0.008896797 | 0.023166023 |
| chr6 | 42608187 | T | C | UBR2 | + | intron | silent | 0.258289703 | 0.257475083 |
| chr6 | 42609127 | G | A | UBR2 | + | intron | silent | 0 | 0.040816327 |
| chr6 | 42609135 | G | A | UBR2 | + | intron | silent | 0 | 0.038834951 |
| chr6 | 42609198 | T | C | UBR2 | + | intron | silent | 0 | 0.002670227 |
| chr6 | 42609222 | A | G | UBR2 | + | intron | silent | 0.011363636 | 0.021192053 |
| chr6 | 42609419 | C | T | UBR2 | + | cds | synonymous | 0 | 0.001321004 |
| chr6 | 42609438 | T | G | UBR2 | + | intron | silent | 0.001410437 | 0.001321004 |
| chr6 | 42610185 | G | A | UBR2 | + | cds | missense | 0 | 0.001321004 |
| chr6 | 42610280 | C | T | UBR2 | + | intron | silent | 0.011283498 | 0.021136063 |
| chr6 | 42611766 | C | T | UBR2 | + | intron | silent | 0 | 0.00141844 |
| chr6 | 42611872 | A | G | UBR2 | + | intron | silent | 0.19887165 | 0.206076618 |
| chr6 | 42611915 | C | T | UBR2 | + | intron | silent | 0.004231312 | 0.005284016 |
| chr6 | 42612004 | G | A | UBR2 | + | cds | missense | 0.001410437 | 0 |
| chr6 | 42612027 | A | G | UBR2 | + | cds | missense | 0.001410437 | 0 |
| chr6 | 42612083 | G | A | UBR2 | + | intron | silent | 0.001410437 | 0 |
| chr6 | 42612101 | A | G | UBR2 | + | intron | silent | 0 | 0.001321004 |
| chr6 | 42612141 | G | T | UBR2 | + | intron | silent | 0.001410437 | 0 |
| chr6 | 42612148 | C | T | UBR2 | + | intron | silent | 0.001410437 | 0 |
| chr6 | 42612312 | C | T | UBR2 | + | intron | silent | 0 | 0 |
| chr6 | 42612316 | C | T | UBR2 | + | intron | silent | 0.001410437 | 0 |
| chr6 | 42612317 | G | A | UBR2 | + | intron | silent | 0.035260931 | 0.05026455 |
| chr6 | 42612343 | T | G | UBR2 | + | intron | silent | 0.001412429 | 0.001324503 |
| chr6 | 42613005 | C | T | UBR2 | + | intron | silent | 0.002971768 | 0 |
| chr6 | 42613069 | C | T | UBR2 | + | intron | silent | 0.028248588 | 0.025099075 |
| chr6 | 42613130 | A | T | UBR2 | + | intron | silent | 0.028208745 | 0.025099075 |
| chr6 | 42613156 | T | C | UBR2 | + | intron | silent | 0.002820874 | 0.002642008 |
| chr6 | 42613166 | G | T | UBR2 | + | intron | silent | 0 | 0.001321004 |
| chr6 | 42613379 | A | G | UBR2 | + | intron | silent | 0.19887165 | 0.206076618 |
| chr6 | 42613390 | A | G | UBR2 | + | intron | silent | 0.001410437 | 0 |
| chr6 | 42613429 | A | G | UBR2 | + | intron | silent | 0.001412429 | 0 |
| chr6 | 42613441 | C | T | UBR2 | + | intron | silent | 0.011315417 | 0.021164021 |
| chr6 | 42615677 | G | A | UBR2 | + | intron | silent | 0.001410437 | 0 |
| chr6 | 42615729 | A | G | UBR2 | + | intron | silent | 0.011283498 | 0.021136063 |
| chr6 | 42615801 | A | C | UBR2 | + | intron | silent | 0.011283498 | 0.021136063 |
| chr6 | 42615803 | G | A | UBR2 | + | intron | silent | 0.001410437 | 0.005284016 |
| chr6 | 42615888 | C | T | UBR2 | + | cds | synonymous | 0 | 0.001321004 |
| chr6 | 42615950 | C | T | UBR2 | + | intron | silent | 0.449929478 | 0.438573316 |
| chr6 | 42617961 | T | G | UBR2 | + | intron | silent | 0.266572638 | 0.254953765 |
| chr6 | 42617978 | T | C | UBR2 | + | intron | silent | 0.039492243 | 0.036988111 |
| chr6 | 42618152 | G | A | UBR2 | + | intron | silent | 0.004231312 | 0.00660502 |
| chr6 | 42618183 | G | A | UBR2 | + | intron | silent | 0 | 0.001321004 |
| chr6 | 42618266 | C | T | UBR2 | + | intron | silent | 0 | 0.0013947 |
| chr6 | 42619585 | C | T | UBR2 | + | intron | silent | 0 | 0.001329787 |
| chr6 | 42619588 | G | A | UBR2 | + | intron | silent | 0.033381713 | 0.021276596 |
| chr6 | 42619612 | G | T | UBR2 | + | intron | silent | 0.001422475 | 0 |
| chr6 | 42619635 | G | A | UBR2 | + | intron | silent | 0 | 0.001324503 |
| chr6 | 42619678 | C | T | UBR2 | + | intron | silent | 0.011283498 | 0.021136063 |
| chr6 | 42619686 | A | G | UBR2 | + | intron | silent | 0 | 0.001321004 |
| chr6 | 42619726 | T | C | UBR2 | + | intron | silent | 0.001410437 | 0 |
| chr6 | 42619727 | A | G | UBR2 | + | intron | silent | 0.001410437 | 0 |
| chr6 | 42619734 | G | A | UBR2 | + | intron | silent | 0 | 0.001321004 |
| chr6 | 42619851 | G | A | UBR2 | + | intron | silent | 0.011283498 | 0.021136063 |
| chr6 | 42620039 | A | G | UBR2 | + | intron | silent | 0 | 0.001324503 |
| chr6 | 42620070 | G | T | UBR2 | + | intron | silent | 0 | 0.001321004 |
| chr6 | 42620165 | A | T | UBR2 | + | intron | silent | 0.001410437 | 0 |
| chr6 | 42620387 | G | A | UBR2 | + | intron | silent | 0 | 0.001321004 |
| chr6 | 42620462 | A | G | UBR2 | + | intron | silent | 0.002832861 | 0 |
| chr6 | 42620475 | C | T | UBR2 | + | intron | silent | 0.00286944 | 0.005291005 |
| chr6 | 42620479 | A | G | UBR2 | + | intron | silent | 0.001436782 | 0 |
| chr6 | 42623317 | T | C | UBR2 | + | intron | silent | 0.001414427 | 0 |
| chr6 | 42623590 | A | G | UBR2 | + | intron | silent | 0 | 0.001331558 |
| chr6 | 42625699 | C | T | UBR2 | + | intron | silent | 0 | 0.001321004 |
| chr6 | 42625700 | G | A | UBR2 | + | intron | silent | 0 | 0.001321004 |
| chr6 | 42625949 | A | G | UBR2 | + | intron | silent | 0.011283498 | 0.021136063 |
| chr6 | 42626074 | A | T | UBR2 | + | intron | silent | 0.001410437 | 0 |
| chr6 | 42626104 | T | A | UBR2 | + | intron | silent | 0 | 0.001321004 |
| chr6 | 42626144 | A | G | UBR2 | + | intron | silent | 0 | 0.00397351 |
| chr6 | 42626150 | T | A | UBR2 | + | intron | silent | 0.023679417 | 0.029310345 |
| chr6 | 42626170 | G | T | UBR2 | + | intron | silent | 0.001410437 | 0 |
| chr6 | 42626317 | T | C | UBR2 | + | intron | silent | 0.011283498 | 0.021136063 |
| chr6 | 42626341 | G | A | UBR2 | + | intron | silent | 0.448519041 | 0.438573316 |
| chr6 | 42626442 | G | A | UBR2 | + | cds | missense | 0 | 0.001321004 |
| chr6 | 42626598 | G | A | UBR2 | + | intron | silent | 0 | 0.002642008 |
| chr6 | 42626610 | A | G | UBR2 | + | intron | silent | 0.001414427 | 0 |
| chr6 | 42626615 | G | T | UBR2 | + | intron | silent | 0 | 0.001321004 |
| chr6 | 42626736 | G | T | UBR2 | + | intron | silent | 0.003115265 | 0.003968254 |
| chr6 | 42627240 | A | T | UBR2 | + | intron | silent | 0 | 0.001428571 |
| chr6 | 42627258 | T | C | UBR2 | + | intron | silent | 0.059322034 | 0.085135135 |
| chr6 | 42627415 | A | T | UBR2 | + | cds | synonymous | 0 | 0.001321004 |
| chr6 | 42627422 | G | A | UBR2 | + | cds | missense | 0 | 0.001321004 |
| chr6 | 42627430 | C | T | UBR2 | + | cds | synonymous | 0.451339915 | 0.438573316 |
| chr6 | 42627434 | G | A | UBR2 | + | cds | missense | 0.328631876 | 0.365918098 |
| chr6 | 42627550 | A | G | UBR2 | + | cds | synonymous | 0.001410437 | 0 |
| chr6 | 42627603 | A | C | UBR2 | + | intron | silent | 0.001410437 | 0.001321004 |
| chr6 | 42629943 | T | C | UBR2 | + | cds | missense | 0.001410437 | 0 |
| chr6 | 42630107 | A | G | UBR2 | + | intron | silent | 0.266572638 | 0.254953765 |
| chr6 | 42630820 | C | G | UBR2 | + | intron | silent | 0.003262643 | 0.002673797 |
| chr6 | 42630921 | T | C | UBR2 | + | intron | silent | 0 | 0.001321004 |
| chr6 | 42630974 | C | T | UBR2 | + | intron | silent | 0 | 0.001321004 |
| chr6 | 42631074 | C | T | UBR2 | + | cds | synonymous | 0 | 0.001321004 |
| chr6 | 42631114 | A | G | UBR2 | + | cds | missense | 0 | 0.001321004 |
| chr6 | 42631142 | G | A | UBR2 | + | cds | missense | 0 | 0.001321004 |
| chr6 | 42631272 | C | T | UBR2 | + | intron | silent | 0.28067701 | 0.248677249 |
| chr6 | 42633086 | A | C | UBR2 | + | intron | silent | 0.001412429 | 0 |
| chr6 | 42633368 | A | G | UBR2 | + | intron | silent | 0.032531825 | 0.025198939 |
| chr6 | 42633412 | T | C | UBR2 | + | intron | silent | 0.001474926 | 0 |
| chr6 | 42633761 | G | A | UBR2 | + | intron | silent | 0 | 0.001322751 |
| chr6 | 42633770 | C | A | UBR2 | + | intron | silent | 0.001412429 | 0.001321004 |
| chr6 | 42633828 | T | C | UBR2 | + | intron | silent | 0 | 0.001322751 |
| chr6 | 42633855 | A | T | UBR2 | + | intron | silent | 0.001410437 | 0 |
| chr6 | 42633977 | G | A | UBR2 | + | cds | missense | 0.001410437 | 0 |
| chr6 | 42634010 | T | C | UBR2 | + | intron | silent | 0.001410437 | 0.001321004 |
| chr6 | 42637708 | A | G | UBR2 | + | intron | silent | 0.001410437 | 0.001321004 |
| chr6 | 42637724 | T | C | UBR2 | + | intron | silent | 0.055007052 | 0.051519155 |
| chr6 | 42637891 | G | A | UBR2 | + | cds | missense | 0 | 0.001321004 |
| chr6 | 42637927 | C | T | UBR2 | + | cds | missense | 0.001410437 | 0.001321004 |
| chr6 | 42637928 | G | A | UBR2 | + | cds | missense | 0 | 0.001321004 |
| chr6 | 42638016 | C | T | UBR2 | + | intron | silent | 0.001410437 | 0 |
| chr6 | 42638122 | T | C | UBR2 | + | intron | silent | 0.00141844 | 0 |
| chr6 | 42638236 | C | T | UBR2 | + | intron | silent | 0.001436782 | 0 |
| chr6 | 42638237 | T | G | UBR2 | + | intron | silent | 0.327116212 | 0.363515313 |
| chr6 | 42638361 | C | T | UBR2 | + | intron | silent | 0 | 0.001322751 |
| chr6 | 42638411 | T | C | UBR2 | + | cds | synonymous | 0.011283498 | 0.021136063 |
| chr6 | 42638491 | A | G | UBR2 | + | intron | silent | 0 | 0.001321004 |
| chr6 | 42638564 | T | C | UBR2 | + | intron | silent | 0.330042313 | 0.365918098 |
| chr6 | 42641620 | T | C | UBR2 | + | cds | missense | 0 | 0.002642008 |
| chr6 | 42641671 | G | A | UBR2 | + | intron | silent | 0.001410437 | 0 |
| chr6 | 42641704 | G | A | UBR2 | + | intron | silent | 0.012693935 | 0.009247028 |
| chr6 | 42641768 | A | C | UBR2 | + | intron | silent | 0.001410437 | 0 |
| chr6 | 42641779 | A | C | UBR2 | + | intron | silent | 0.014104372 | 0.018494055 |
| chr6 | 42641848 | C | T | UBR2 | + | intron | silent | 0.001410437 | 0.001321004 |
| chr6 | 42641864 | C | T | UBR2 | + | cds | missense | 0 | 0.001321004 |
| chr6 | 42641962 | A | C | UBR2 | + | intron | silent | 0.001410437 | 0.001321004 |
| chr6 | 42644062 | A | G | UBR2 | + | intron | silent | 0 | 0 |
| chr6 | 42644095 | T | C | UBR2 | + | intron | silent | 0 | 0.003994674 |
| chr6 | 42644473 | A | G | UBR2 | + | intron | silent | 0.004231312 | 0.007926024 |
| chr6 | 42644585 | T | C | UBR2 | + | cds | synonymous | 0 | 0.001321004 |
| chr6 | 42644620 | G | A | UBR2 | + | intron | silent | 0.001410437 | 0.002642008 |
| chr6 | 42644623 | A | G | UBR2 | + | intron | silent | 0.328631876 | 0.365918098 |
| chr6 | 42644626 | T | C | UBR2 | + | intron | silent | 0.001410437 | 0 |
| chr6 | 42644633 | A | G | UBR2 | + | intron | silent | 0.001410437 | 0 |
| chr6 | 42644672 | A | G | UBR2 | + | intron | silent | 0.002820874 | 0 |
| chr6 | 42644684 | G | A | UBR2 | + | intron | silent | 0.001410437 | 0 |
| chr6 | 42646274 | T | G | UBR2 | + | intron | silent | 0.001410437 | 0 |
| chr6 | 42646472 | G | C | UBR2 | + | intron | silent | 0 | 0.001321004 |
| chr6 | 42646518 | T | A | UBR2 | + | intron | silent | 0 | 0 |
| chr6 | 42646598 | A | T | UBR2 | + | intron | silent | 0.003257329 | 0.001519757 |
| chr6 | 42647338 | A | G | UBR2 | + | intron | silent | 0.001410437 | 0 |
| chr6 | 42647396 | C | G | UBR2 | + | intron | silent | 0.002820874 | 0.001321004 |
| chr6 | 42647466 | T | C | UBR2 | + | cds | synonymous | 0.001410437 | 0 |
| chr6 | 42647578 | T | G | UBR2 | + | intron | silent | 0 | 0.001321004 |
| chr6 | 42647632 | T | C | UBR2 | + | intron | silent | 0.001412429 | 0 |
| chr6 | 42647662 | A | C | UBR2 | + | intron | silent | 0.001416431 | 0 |
| chr6 | 42650772 | C | T | UBR2 | + | intron | silent | 0.287729196 | 0.263227513 |
| chr6 | 42650801 | G | A | UBR2 | + | cds | missense | 0.001410437 | 0 |
| chr6 | 42650823 | T | C | UBR2 | + | cds | synonymous | 0.001410437 | 0 |
| chr6 | 42650953 | A | G | UBR2 | + | intron | silent | 0 | 0.001324503 |
| chr6 | 42652384 | A | C | UBR2 | + | intron | silent | 0.092067989 | 0.105680317 |
| chr6 | 42652414 | G | A | UBR2 | + | intron | silent | 0.001412429 | 0 |
| chr6 | 42652441 | G | A | UBR2 | + | intron | silent | 0 | 0.001321004 |
| chr6 | 42652447 | G | A | UBR2 | + | intron | silent | 0.001410437 | 0 |
| chr6 | 42652563 | C | A | UBR2 | + | cds | missense | 0.001410437 | 0 |
| chr6 | 42652667 | G | A | UBR2 | + | intron | silent | 0.031029619 | 0.030383091 |
| chr6 | 42652696 | G | A | UBR2 | + | intron | silent | 0 | 0.001321004 |
| chr6 | 42652726 | T | C | UBR2 | + | intron | silent | 0 | 0.001321004 |
| chr6 | 42652790 | G | A | UBR2 | + | intron | silent | 0 | 0.001715266 |
| chr6 | 42652791 | C | T | UBR2 | + | intron | silent | 0.00330033 | 0.003484321 |
| chr6 | 42655991 | A | G | UBR2 | + | cds | missense | 0 | 0.001321004 |
| chr6 | 42656280 | A | T | UBR2 | + | intron | silent | 0.001508296 | 0 |
| chr6 | 42657186 | A | G | UBR2 | + | intron | silent | 0.001410437 | 0.001321004 |
| chr6 | 42657231 | T | C | UBR2 | + | intron | silent | 0.018335684 | 0.015852048 |
| chr6 | 42657348 | A | G | UBR2 | + | cds | missense | 0.001410437 | 0 |
| chr6 | 42657456 | C | T | UBR2 | + | intron | silent | 0.001410437 | 0.001321004 |
| chr6 | 42657457 | C | T | UBR2 | + | intron | silent | 0 | 0.001321004 |
| chr6 | 42658752 | T | C | UBR2 | + | intron | silent | 0.406205924 | 0.401585205 |
| chr6 | 42659089 | C | T | UBR2 | + | utr3 | silent | 0 | 0.001373626 |
| chrX | 132159845 | T | C | USP26 | - | cds | missense | 0 | 0.001321004 |
| chrX | 132160223 | G | A | USP26 | - | cds | missense | 0 | 0.001322751 |
| chrX | 132160312 | C | T | USP26 | - | cds | missense | 0 | 0.001322751 |
| chrX | 132160342 | T | C | USP26 | - | cds | missense | 0 | 0.00132626 |
| chrX | 132160810 | T | A | USP26 | - | cds | missense | 0 | 0.001321004 |
| chrX | 132161172 | C | T | USP26 | - | cds | missense | 0 | 0.001321004 |
| chrX | 132161301 | G | T | USP26 | - | cds | synonymous | 0 | 0.001321004 |
| chrX | 132161344 | T | C | USP26 | - | cds | missense | 0.001410437 | 0 |
| chrX | 132161813 | T | C | USP26 | - | cds | missense | 0.001410437 | 0.001321004 |
| chrX | 132161936 | C | T | USP26 | - | cds | missense | 0 | 0.001321004 |
| chrX | 132161937 | G | A | USP26 | - | cds | synonymous | 0.001410437 | 0 |
| chrX | 132161993 | A | G | USP26 | - | cds | synonymous | 0 | 0.001331558 |
| chrX | 132162124 | A | G | USP26 | - | cds | missense | 0 | 0.001322751 |
| chrX | 132162137 | G | A | USP26 | - | cds | synonymous | 0.001472754 | 0 |
| chr18 | 44559278 | G | A | TCEB3B | - | utr3 | silent | 0 | 0.001745201 |
| chr18 | 44559306 | C | T | TCEB3B | - | utr3 | silent | 0.001459854 | 0.004219409 |
| chr18 | 44559316 | A | G | TCEB3B | - | utr3 | silent | 0.001438849 | 0 |
| chr18 | 44559359 | A | G | TCEB3B | - | utr3 | silent | 0.004243281 | 0.002645503 |
| chr18 | 44559687 | G | T | TCEB3B | - | cds | missense | 0 | 0.001324503 |
| chr18 | 44559730 | C | T | TCEB3B | - | cds | missense | 0.001410437 | 0.001324503 |
| chr18 | 44559732 | T | C | TCEB3B | - | cds | missense | 0.001410437 | 0 |
| chr18 | 44559841 | G | T | TCEB3B | - | cds | missense | 0 | 0.001321004 |
| chr18 | 44559844 | G | A | TCEB3B | - | cds | missense | 0.009873061 | 0.015852048 |
| chr18 | 44559927 | C | A | TCEB3B | - | cds | missense | 0.002820874 | 0.010568032 |
| chr18 | 44559998 | G | T | TCEB3B | - | cds | missense | 0.001410437 | 0 |
| chr18 | 44560038 | C | A | TCEB3B | - | cds | missense | 0.009873061 | 0.015852048 |
| chr18 | 44560067 | C | G | TCEB3B | - | cds | missense | 0 | 0.001321004 |
| chr18 | 44560109 | A | G | TCEB3B | - | cds | synonymous | 0 | 0.001321004 |
| chr18 | 44560123 | C | T | TCEB3B | - | cds | missense | 0.009873061 | 0.015852048 |
| chr18 | 44560143 | C | T | TCEB3B | - | cds | missense | 0 | 0.001321004 |
| chr18 | 44560208 | C | T | TCEB3B | - | cds | synonymous | 0 | 0.003963012 |
| chr18 | 44560277 | C | T | TCEB3B | - | cds | synonymous | 0 | 0.001338688 |
| chr18 | 44560278 | G | A | TCEB3B | - | cds | missense | 0 | 0.001344086 |
| chr18 | 44560300 | C | T | TCEB3B | - | cds | missense | 0.12191582 | 0.111111111 |
| chr18 | 44560337 | G | T | TCEB3B | - | cds | synonymous | 0.011283498 | 0.001321004 |
| chr18 | 44560339 | C | G | TCEB3B | - | cds | missense | 0.001410437 | 0 |
| chr18 | 44560359 | G | A | TCEB3B | - | cds | missense | 0 | 0.001321004 |
| chr18 | 44560429 | C | A | TCEB3B | - | cds | missense | 0.124118477 | 0.103038309 |
| chr18 | 44560456 | T | G | TCEB3B | - | cds | missense | 0 | 0.001321004 |
| chr18 | 44560494 | A | C | TCEB3B | - | cds | missense | 0 | 0.001321004 |
| chr18 | 44560560 | G | T | TCEB3B | - | cds | missense | 0 | 0.001321004 |
| chr18 | 44560606 | G | C | TCEB3B | - | cds | missense | 0 | 0.001321004 |
| chr18 | 44560688 | C | G | TCEB3B | - | cds | missense | 0 | 0.001321004 |
| chr18 | 44560691 | C | T | TCEB3B | - | cds | synonymous | 0.001410437 | 0 |
| chr18 | 44560693 | T | C | TCEB3B | - | cds | missense | 0.012693935 | 0.01321004 |
| chr18 | 44560697 | C | T | TCEB3B | - | cds | synonymous | 0.002820874 | 0 |
| chr18 | 44560770 | C | A | TCEB3B | - | cds | missense | 0 | 0.001321004 |
| chr18 | 44560804 | C | T | TCEB3B | - | cds | missense | 0.001410437 | 0.002642008 |
| chr18 | 44560875 | C | A | TCEB3B | - | cds | missense | 0.128349788 | 0.109643329 |
| chr18 | 44560979 | G | A | TCEB3B | - | cds | synonymous | 0 | 0.00132626 |
| chr18 | 44561097 | A | T | TCEB3B | - | cds | missense | 0.001424501 | 0 |
| chr18 | 44561100 | C | G | TCEB3B | - | cds | missense | 0.123755334 | 0.10326087 |
| chr18 | 44561118 | G | T | TCEB3B | - | cds | missense | 0.001412429 | 0 |
| chr18 | 44561124 | G | C | TCEB3B | - | cds | missense | 0.004249292 | 0.00400534 |
| chr18 | 44561133 | T | C | TCEB3B | - | cds | missense | 0.001414427 | 0.001336898 |
| chr18 | 44561292 | T | G | TCEB3B | - | cds | missense | 0.083569405 | 0.082119205 |
| chr18 | 44561302 | A | C | TCEB3B | - | cds | missense | 0 | 0.001324503 |
| chr18 | 44561319 | T | C | TCEB3B | - | cds | missense | 0.002820874 | 0.005298013 |
| chr18 | 44561321 | G | C | TCEB3B | - | cds | missense | 0.001410437 | 0.00397351 |
| chr18 | 44561396 | G | A | TCEB3B | - | cds | synonymous | 0.004243281 | 0.001324503 |
| chr18 | 44561516 | C | G | TCEB3B | - | cds | synonymous | 0.129814551 | 0.114482759 |
| chr18 | 44561601 | T | C | TCEB3B | - | cds | missense | 0 | 0.00204918 |
| chr18 | 44561619 | G | C | TCEB3B | - | cds | missense | 0.01025641 | 0.016990291 |
| chr18 | 44561630 | C | T | TCEB3B | - | cds | synonymous | 0.010344828 | 0.018648019 |
| chr18 | 44561704 | A | C | TCEB3B | - | utr5 | silent | 0.003745318 | 0 |
| chr9 | 110062236 | A | G | RAD23B | + | intron | silent | 0.001972387 | 0.005649718 |
| chr9 | 110062332 | A | G | RAD23B | + | intron | silent | 0 | 0.00132626 |
| chr9 | 110062365 | T | A | RAD23B | + | intron | silent | 0.001416431 | 0.00397351 |
| chr9 | 110062573 | T | C | RAD23B | + | intron | silent | 0 | 0.001324503 |
| chr9 | 110062591 | A | G | RAD23B | + | intron | silent | 0.002886003 | 0.001328021 |
| chr9 | 110062594 | T | G | RAD23B | + | intron | silent | 0.002894356 | 0.005312085 |
| chr9 | 110062613 | C | A | RAD23B | + | intron | silent | 0.004398827 | 0.015978695 |
| chr9 | 110062663 | A | G | RAD23B | + | intron | silent | 0.001715266 | 0.002853067 |
| chr9 | 110064174 | A | G | RAD23B | + | intron | silent | 0 | 0.001328021 |
| chr9 | 110064221 | C | T | RAD23B | + | intron | silent | 0.001422475 | 0 |
| chr9 | 110064265 | G | A | RAD23B | + | intron | silent | 0.011331445 | 0.025165563 |
| chr9 | 110064282 | C | G | RAD23B | + | intron | silent | 0.004237288 | 0 |
| chr9 | 110064326 | C | T | RAD23B | + | cds | synonymous | 0.001412429 | 0 |
| chr9 | 110064427 | T | C | RAD23B | + | intron | silent | 0 | 0.001324503 |
| chr9 | 110064482 | T | A | RAD23B | + | intron | silent | 0.038135593 | 0.025198939 |
| chr9 | 110064564 | T | C | RAD23B | + | intron | silent | 0.001545595 | 0 |
| chr9 | 110068480 | A | G | RAD23B | + | intron | silent | 0.002518892 | 0 |
| chr9 | 110068625 | T | A | RAD23B | + | intron | silent | 0.001410437 | 0 |
| chr9 | 110068823 | C | T | RAD23B | + | cds | missense | 0.001410437 | 0.00660502 |
| chr9 | 110068921 | A | G | RAD23B | + | cds | missense | 0.001410437 | 0 |
| chr9 | 110069018 | C | T | RAD23B | + | intron | silent | 0.004267425 | 0.003963012 |
| chr9 | 110069029 | A | G | RAD23B | + | intron | silent | 0.005706134 | 0.001321004 |
| chr9 | 110069075 | T | C | RAD23B | + | intron | silent | 0 | 0.001367989 |
| chr9 | 110073776 | C | T | RAD23B | + | intron | silent | 0.003460208 | 0 |
| chr9 | 110073785 | A | G | RAD23B | + | intron | silent | 0.001666667 | 0 |
| chr9 | 110073858 | A | G | RAD23B | + | intron | silent | 0.002828854 | 0.00132626 |
| chr9 | 110073867 | C | G | RAD23B | + | intron | silent | 0.001412429 | 0 |
| chr9 | 110073885 | A | G | RAD23B | + | intron | silent | 0 | 0.001324503 |
| chr9 | 110073925 | A | G | RAD23B | + | intron | silent | 0 | 0.006613757 |
| chr9 | 110073931 | T | C | RAD23B | + | intron | silent | 0 | 0.001322751 |
| chr9 | 110073965 | C | T | RAD23B | + | cds | missense | 0 | 0.001321004 |
| chr9 | 110074004 | C | G | RAD23B | + | cds | missense | 0 | 0.001322751 |
| chr9 | 110080860 | G | A | RAD23B | + | intron | silent | 0.00623053 | 0.00400534 |
| chr9 | 110080967 | A | G | RAD23B | + | intron | silent | 0.043723554 | 0.059445178 |
| chr9 | 110080976 | T | A | RAD23B | + | intron | silent | 0.011283498 | 0.025099075 |
| chr9 | 110080997 | A | G | RAD23B | + | intron | silent | 0.001410437 | 0 |
| chr9 | 110081018 | A | G | RAD23B | + | intron | silent | 0.049365303 | 0.066050198 |
| chr9 | 110081331 | T | A | RAD23B | + | intron | silent | 0.359322034 | 0.379020979 |
| chr9 | 110081358 | C | T | RAD23B | + | intron | silent | 0.025462963 | 0.021035599 |
| chr9 | 110084182 | A | G | RAD23B | + | intron | silent | 0 | 0.001321004 |
| chr9 | 110084208 | T | C | RAD23B | + | intron | silent | 0 | 0.001321004 |
| chr9 | 110084305 | C | G | RAD23B | + | cds | synonymous | 0.001410437 | 0 |
| chr9 | 110084328 | C | T | RAD23B | + | cds | missense | 0.354019746 | 0.375165125 |
| chr9 | 110084373 | C | T | RAD23B | + | cds | missense | 0 | 0.001321004 |
| chr9 | 110084410 | A | G | RAD23B | + | intron | silent | 0 | 0.001321004 |
| chr9 | 110084417 | G | C | RAD23B | + | intron | silent | 0.045133992 | 0.042272127 |
| chr9 | 110084484 | C | T | RAD23B | + | intron | silent | 0.001410437 | 0 |
| chr9 | 110084505 | A | T | RAD23B | + | intron | silent | 0.001414427 | 0.002649007 |
| chr9 | 110085992 | G | C | RAD23B | + | intron | silent | 0.050295858 | 0.066577896 |
| chr9 | 110086064 | T | G | RAD23B | + | intron | silent | 0 | 0.002642008 |
| chr9 | 110086068 | A | G | RAD23B | + | intron | silent | 0.002820874 | 0 |
| chr9 | 110086149 | G | A | RAD23B | + | intron | silent | 0.043723554 | 0.059445178 |
| chr9 | 110086178 | C | T | RAD23B | + | cds | synonymous | 0 | 0.001321004 |
| chr9 | 110086256 | G | C | RAD23B | + | cds | synonymous | 0.008462623 | 0.017173052 |
| chr9 | 110086364 | C | A | RAD23B | + | intron | silent | 0.001410437 | 0 |
| chr9 | 110086374 | C | G | RAD23B | + | intron | silent | 0 | 0.001321004 |
| chr9 | 110086380 | C | G | RAD23B | + | intron | silent | 0 | 0.001321004 |
| chr9 | 110086930 | A | G | RAD23B | + | intron | silent | 0 | 0.002673797 |
| chr9 | 110086951 | A | G | RAD23B | + | intron | silent | 0.011560694 | 0.025265957 |
| chr9 | 110087007 | C | A | RAD23B | + | intron | silent | 0 | 0.001321004 |
| chr9 | 110087248 | T | A | RAD23B | + | cds | missense | 0 | 0.001321004 |
| chr9 | 110087329 | C | T | RAD23B | + | intron | silent | 0.002820874 | 0.00660502 |
| chr9 | 110087464 | A | G | RAD23B | + | intron | silent | 0.009009009 | 0 |
| chr9 | 110091721 | A | G | RAD23B | + | intron | silent | 0 | 0.00132626 |
| chr9 | 110091738 | C | T | RAD23B | + | intron | silent | 0 | 0.00132626 |
| chr9 | 110091971 | G | A | RAD23B | + | utr3 | silent | 0 | 0.001321004 |
| chr17 | 70117556 | G | A | SOX9 | + | cds | missense | 0 | 0.001398601 |
| chr17 | 70117616 | G | A | SOX9 | + | cds | synonymous | 0 | 0.001344086 |
| chr17 | 70117678 | A | T | SOX9 | + | cds | missense | 0 | 0.001324503 |
| chr17 | 70117928 | C | T | SOX9 | + | cds | synonymous | 0 | 0.001344086 |
| chr17 | 70118054 | C | T | SOX9 | + | intron | silent | 0.038135593 | 0.017094017 |
| chr17 | 70118699 | G | A | SOX9 | + | intron | silent | 0.197215777 | 0.191740413 |
| chr17 | 70118905 | G | A | SOX9 | + | cds | synonymous | 0.001414427 | 0.001324503 |
| chr17 | 70118935 | C | T | SOX9 | + | cds | synonymous | 0.024113475 | 0.029139073 |
| chr17 | 70118950 | C | T | SOX9 | + | cds | synonymous | 0.001414427 | 0 |
| chr17 | 70119013 | G | C | SOX9 | + | cds | missense | 0 | 0.001322751 |
| chr17 | 70119020 | A | T | SOX9 | + | cds | missense | 0 | 0.001322751 |
| chr17 | 70119107 | C | T | SOX9 | + | cds | missense | 0 | 0.001355014 |
| chr17 | 70119151 | C | T | SOX9 | + | intron | silent | 0.001497006 | 0.004464286 |
| chr17 | 70119245 | C | G | SOX9 | + | intron | silent | 0.191919192 | 0.174496644 |
| chr17 | 70119610 | G | A | SOX9 | + | intron | silent | 0.004444444 | 0 |
| chr17 | 70119668 | C | T | SOX9 | + | intron | silent | 0 | 0.001517451 |
| chr17 | 70120144 | G | T | SOX9 | + | cds | synonymous | 0.001552795 | 0 |
| chr17 | 70120222 | C | G | SOX9 | + | cds | missense | 0 | 0.001321004 |
| chr17 | 70120279 | C | T | SOX9 | + | cds | synonymous | 0 | 0.001321004 |
| chr17 | 70120403 | A | G | SOX9 | + | cds | missense | 0.002824859 | 0.005291005 |
| chr17 | 70120417 | G | A | SOX9 | + | cds | synonymous | 0.002820874 | 0 |
| chr17 | 70120509 | C | T | SOX9 | + | cds | missense | 0.00143472 | 0 |
| chr17 | 70120551 | A | C | SOX9 | + | utr3 | silent | 0.192023634 | 0.197070573 |
| chr17 | 70120661 | C | T | SOX9 | + | utr3 | silent | 0 | 0.004672897 |
| chr1 | 169435056 | T | C | SLC19A2 | - | utr3 | silent | 0.001410437 | 0.002642008 |
| chr1 | 169435142 | T | C | SLC19A2 | - | cds | missense | 0 | 0.001321004 |
| chr1 | 169435246 | A | G | SLC19A2 | - | intron | silent | 0 | 0.001321004 |
| chr1 | 169435260 | G | A | SLC19A2 | - | intron | silent | 0 | 0.001321004 |
| chr1 | 169435328 | C | T | SLC19A2 | - | intron | silent | 0.001412429 | 0.003963012 |
| chr1 | 169437153 | C | A | SLC19A2 | - | intron | silent | 0.240181269 | 0.242587601 |
| chr1 | 169437185 | G | T | SLC19A2 | - | intron | silent | 0.001422475 | 0.001321004 |
| chr1 | 169437197 | G | A | SLC19A2 | - | intron | silent | 0.001420455 | 0 |
| chr1 | 169437327 | G | A | SLC19A2 | - | intron | silent | 0 | 0.001321004 |
| chr1 | 169437392 | A | G | SLC19A2 | - | cds | missense | 0.145275035 | 0.125495376 |
| chr1 | 169437402 | G | A | SLC19A2 | - | cds | missense | 0 | 0.001321004 |
| chr1 | 169437426 | T | C | SLC19A2 | - | cds | missense | 0 | 0.001321004 |
| chr1 | 169437436 | T | C | SLC19A2 | - | cds | synonymous | 0 | 0.001321004 |
| chr1 | 169437533 | G | C | SLC19A2 | - | intron | silent | 0.001410437 | 0 |
| chr1 | 169437574 | A | G | SLC19A2 | - | intron | silent | 0.01980198 | 0.013227513 |
| chr1 | 169437594 | C | G | SLC19A2 | - | intron | silent | 0.00152207 | 0.002702703 |
| chr1 | 169437854 | T | A | SLC19A2 | - | intron | silent | 0 | 0.001321004 |
| chr1 | 169437887 | T | C | SLC19A2 | - | cds | missense | 0.001410437 | 0 |
| chr1 | 169437892 | T | C | SLC19A2 | - | cds | missense | 0 | 0.003963012 |
| chr1 | 169438025 | A | G | SLC19A2 | - | cds | synonymous | 0.133991537 | 0.130779392 |
| chr1 | 169438176 | T | C | SLC19A2 | - | intron | silent | 0.001414427 | 0 |
| chr1 | 169438180 | A | G | SLC19A2 | - | intron | silent | 0.246808511 | 0.240422721 |
| chr1 | 169438213 | A | G | SLC19A2 | - | intron | silent | 0.001564945 | 0.002754821 |
| chr1 | 169439087 | C | T | SLC19A2 | - | intron | silent | 0 | 0.001321004 |
| chr1 | 169439174 | C | T | SLC19A2 | - | intron | silent | 0.007052186 | 0.007926024 |
| chr1 | 169439217 | C | T | SLC19A2 | - | cds | missense | 0.002820874 | 0.001321004 |
| chr1 | 169439473 | A | G | SLC19A2 | - | intron | silent | 0.001410437 | 0 |
| chr1 | 169446356 | A | G | SLC19A2 | - | intron | silent | 0 | 0.001321004 |
| chr1 | 169446623 | T | C | SLC19A2 | - | cds | missense | 0 | 0.001321004 |
| chr1 | 169446672 | C | T | SLC19A2 | - | cds | synonymous | 0 | 0.001321004 |
| chr1 | 169446687 | C | T | SLC19A2 | - | cds | synonymous | 0.001410437 | 0 |
| chr1 | 169446742 | A | G | SLC19A2 | - | cds | missense | 0 | 0.002642008 |
| chr1 | 169446988 | T | C | SLC19A2 | - | cds | missense | 0 | 0.001321004 |
| chr1 | 169447181 | T | C | SLC19A2 | - | intron | silent | 0 | 0.006849315 |
| chr1 | 169454701 | C | T | SLC19A2 | - | intron | silent | 0.003496503 | 0 |
| chr1 | 169454724 | T | C | SLC19A2 | - | intron | silent | 0.00209205 | 0 |
| chr1 | 169454973 | G | C | SLC19A2 | - | cds | missense | 0.003058104 | 0 |
| chr1 | 169455008 | G | A | SLC19A2 | - | utr5 | silent | 0.250460405 | 0.32748538 |
| chr1 | 169455116 | C | T | SLC19A2 | - | utr5 | silent | 0.002398082 | 0 |
| chr12 | 48238333 | G | A | VDR | - | utr3 | silent | 0 | 0.004219409 |
| chr12 | 48238390 | G | A | VDR | - | utr3 | silent | 0.001464129 | 0 |
| chr12 | 48238444 | G | A | VDR | - | utr3 | silent | 0.001436782 | 0 |
| chr12 | 48238456 | G | T | VDR | - | utr3 | silent | 0 | 0.001362398 |
| chr12 | 48238556 | G | A | VDR | - | cds | synonymous | 0 | 0.001321004 |
| chr12 | 48238607 | G | T | VDR | - | cds | synonymous | 0.01551481 | 0.014531044 |
| chr12 | 48238661 | G | T | VDR | - | cds | synonymous | 0.005641749 | 0.005284016 |
| chr12 | 48238682 | G | A | VDR | - | cds | synonymous | 0.002820874 | 0.009247028 |
| chr12 | 48238705 | G | A | VDR | - | cds | missense | 0 | 0.001321004 |
| chr12 | 48238710 | C | T | VDR | - | cds | missense | 0 | 0.001321004 |
| chr12 | 48238757 | A | G | VDR | - | cds | synonymous | 0.105932203 | 0.091149273 |
| chr12 | 48238764 | G | A | VDR | - | cds | missense | 0.002820874 | 0 |
| chr12 | 48238812 | A | G | VDR | - | intron | silent | 0 | 0.001358696 |
| chr12 | 48238820 | G | A | VDR | - | intron | silent | 0 | 0.001390821 |
| chr12 | 48238837 | C | A | VDR | - | intron | silent | 0.488059701 | 0.496923077 |
| chr12 | 48238883 | C | T | VDR | - | intron | silent | 0.005008347 | 0.012195122 |
| chr12 | 48238900 | C | G | VDR | - | intron | silent | 0.421588595 | 0.474903475 |
| chr12 | 48239962 | C | A | VDR | - | intron | silent | 0.005319149 | 0.004511278 |
| chr12 | 48239985 | G | T | VDR | - | intron | silent | 0.001536098 | 0.001369863 |
| chr12 | 48240077 | G | A | VDR | - | intron | silent | 0 | 0.001321004 |
| chr12 | 48240095 | C | T | VDR | - | intron | silent | 0.001410437 | 0.001321004 |
| chr12 | 48240170 | G | A | VDR | - | cds | synonymous | 0.001410437 | 0 |
| chr12 | 48240258 | C | T | VDR | - | intron | silent | 0 | 0.005284016 |
| chr12 | 48240262 | G | A | VDR | - | intron | silent | 0.001410437 | 0 |
| chr12 | 48240314 | C | G | VDR | - | intron | silent | 0.001410437 | 0 |
| chr12 | 48240344 | C | T | VDR | - | intron | silent | 0.001410437 | 0 |
| chr12 | 48240379 | C | T | VDR | - | intron | silent | 0.002820874 | 0 |
| chr12 | 48240416 | C | T | VDR | - | intron | silent | 0.008462623 | 0.002642008 |
| chr12 | 48240530 | A | G | VDR | - | cds | synonymous | 0.001410437 | 0 |
| chr12 | 48240533 | T | C | VDR | - | cds | missense | 0 | 0.001321004 |
| chr12 | 48240595 | C | G | VDR | - | intron | silent | 0.001410437 | 0.002642008 |
| chr12 | 48249259 | C | T | VDR | - | intron | silent | 0.123430962 | 0.089700997 |
| chr12 | 48249260 | G | A | VDR | - | intron | silent | 0.003968254 | 0.003194888 |
| chr12 | 48249279 | C | T | VDR | - | intron | silent | 0.001545595 | 0 |
| chr12 | 48249280 | G | A | VDR | - | intron | silent | 0.045454545 | 0.047814208 |
| chr12 | 48249340 | T | C | VDR | - | intron | silent | 0.001410437 | 0.001321004 |
| chr12 | 48249513 | G | C | VDR | - | cds | missense | 0 | 0.001321004 |
| chr12 | 48249638 | C | T | VDR | - | intron | silent | 0 | 0.001321004 |
| chr12 | 48249768 | G | A | VDR | - | intron | silent | 0.001724138 | 0 |
| chr12 | 48250716 | C | A | VDR | - | intron | silent | 0 | 0.001736111 |
| chr12 | 48250775 | G | A | VDR | - | intron | silent | 0.001420455 | 0 |
| chr12 | 48250776 | C | T | VDR | - | intron | silent | 0.069602273 | 0.048 |
| chr12 | 48250786 | C | T | VDR | - | intron | silent | 0.001414427 | 0 |
| chr12 | 48250794 | T | A | VDR | - | intron | silent | 0.001412429 | 0 |
| chr12 | 48250901 | G | A | VDR | - | intron | silent | 0 | 0.001321004 |
| chr12 | 48250936 | A | C | VDR | - | cds | missense | 0.001410437 | 0 |
| chr12 | 48250969 | G | T | VDR | - | cds | missense | 0 | 0.001321004 |
| chr12 | 48251076 | C | T | VDR | - | intron | silent | 0.001410437 | 0.001321004 |
| chr12 | 48251114 | T | C | VDR | - | intron | silent | 0.021156559 | 0.015852048 |
| chr12 | 48251259 | G | A | VDR | - | intron | silent | 0.018335684 | 0.021136063 |
| chr12 | 48251406 | C | A | VDR | - | cds | missense | 0 | 0.001321004 |
| chr12 | 48251439 | G | A | VDR | - | cds | missense | 0 | 0.001321004 |
| chr12 | 48251533 | C | T | VDR | - | intron | silent | 0.497175141 | 0.48348745 |
| chr12 | 48251542 | G | A | VDR | - | intron | silent | 0.349858357 | 0.400530504 |
| chr12 | 48258737 | G | A | VDR | - | intron | silent | 0.001426534 | 0 |
| chr12 | 48258743 | C | G | VDR | - | intron | silent | 0.001416431 | 0.001351351 |
| chr12 | 48258759 | C | G | VDR | - | intron | silent | 0 | 0.002649007 |
| chr12 | 48258891 | G | A | VDR | - | cds | synonymous | 0.001410437 | 0.001321004 |
| chr12 | 48258907 | C | T | VDR | - | cds | missense | 0 | 0.001322751 |
| chr12 | 48258951 | C | A | VDR | - | cds | missense | 0 | 0.001410437 |
| chr12 | 48258960 | C | T | VDR | - | cds | synonymous | 0 | 0.001336898 |
| chr12 | 48259014 | C | G | VDR | - | intron | silent | 0.001412429 | 0 |
| chr12 | 48259024 | C | T | VDR | - | intron | silent | 0 | 0.001333333 |
| chr12 | 48259025 | G | A | VDR | - | intron | silent | 0 | 0.001336898 |
| chr12 | 48259126 | C | T | VDR | - | intron | silent | 0.10371517 | 0.092682927 |
| chr12 | 48272552 | A | G | VDR | - | intron | silent | 0 | 0.001438849 |
| chr12 | 48272631 | C | T | VDR | - | intron | silent | 0.001416431 | 0.002656042 |
| chr12 | 48272633 | C | T | VDR | - | intron | silent | 0.00141844 | 0 |
| chr12 | 48272680 | C | T | VDR | - | intron | silent | 0.001410437 | 0.002642008 |
| chr12 | 48272693 | A | G | VDR | - | intron | silent | 0 | 0.001321004 |
| chr12 | 48272743 | G | A | VDR | - | intron | silent | 0.086036671 | 0.087186262 |
| chr12 | 48272775 | C | T | VDR | - | cds | missense | 0 | 0.001321004 |
| chr12 | 48272845 | G | A | VDR | - | cds | missense | 0 | 0.001321004 |
| chr12 | 48272883 | G | A | VDR | - | cds | missense | 0.001410437 | 0 |
| chr12 | 48272895 | A | G | VDR | - | cds | missense | 0.210155148 | 0.237780713 |
| chr12 | 48272949 | C | T | VDR | - | intron | silent | 0 | 0.001443001 |
| chr12 | 48272968 | C | T | VDR | - | intron | silent | 0.001869159 | 0.002347418 |
| chr21 | 46935400 | G | A | SLC19A1 | - | utr3 | silent | 0.003546099 | 0 |
| chr21 | 46935430 | G | A | SLC19A1 | - | utr3 | silent | 0.00486618 | 0 |
| chr21 | 46935446 | A | G | SLC19A1 | - | utr3 | silent | 0.038636364 | 0.056 |
| chr21 | 46935481 | C | A | SLC19A1 | - | utr3 | silent | 0.016085791 | 0.015151515 |
| chr21 | 46935578 | G | A | SLC19A1 | - | cds | synonymous | 0.001410437 | 0 |
| chr21 | 46935762 | G | A | SLC19A1 | - | cds | missense | 0 | 0.001321004 |
| chr21 | 46935773 | C | T | SLC19A1 | - | cds | synonymous | 0.001410437 | 0 |
| chr21 | 46935784 | C | T | SLC19A1 | - | cds | missense | 0 | 0.001321004 |
| chr21 | 46935802 | G | A | SLC19A1 | - | cds | synonymous | 0.004237288 | 0.003963012 |
| chr21 | 46935945 | G | A | SLC19A1 | - | cds | missense | 0.001612903 | 0 |
| chr21 | 46935981 | C | T | SLC19A1 | - | cds | missense | 0.003278689 | 0 |
| chr21 | 46945539 | C | A | SLC19A1 | - | intron | silent | 0.001692047 | 0 |
| chr21 | 46945548 | C | T | SLC19A1 | - | intron | silent | 0.036624204 | 0.024347826 |
| chr21 | 46945615 | G | A | SLC19A1 | - | intron | silent | 0.12446959 | 0.106809079 |
| chr21 | 46945701 | C | T | SLC19A1 | - | intron | silent | 0 | 0.001321004 |
| chr21 | 46945709 | C | T | SLC19A1 | - | intron | silent | 0.001410437 | 0 |
| chr21 | 46945782 | G | T | SLC19A1 | - | cds | synonymous | 0.008462623 | 0.002642008 |
| chr21 | 46945812 | C | T | SLC19A1 | - | cds | synonymous | 0 | 0.001321004 |
| chr21 | 46945879 | G | A | SLC19A1 | - | intron | silent | 0.002820874 | 0 |
| chr21 | 46945896 | C | G | SLC19A1 | - | intron | silent | 0 | 0.001329787 |
| chr21 | 46950587 | C | T | SLC19A1 | - | intron | silent | 0.008912656 | 0.003134796 |
| chr21 | 46950622 | G | A | SLC19A1 | - | intron | silent | 0.00308642 | 0 |
| chr21 | 46950645 | G | A | SLC19A1 | - | intron | silent | 0.001428571 | 0 |
| chr21 | 46950697 | C | T | SLC19A1 | - | cds | missense | 0.001416431 | 0 |
| chr21 | 46950745 | G | A | SLC19A1 | - | cds | synonymous | 0 | 0.001336898 |
| chr21 | 46950816 | G | A | SLC19A1 | - | cds | missense | 0 | 0.001342282 |
| chr21 | 46950837 | C | T | SLC19A1 | - | cds | missense | 0.001412429 | 0 |
| chr21 | 46950840 | G | A | SLC19A1 | - | cds | missense | 0 | 0.001340483 |
| chr21 | 46950863 | C | T | SLC19A1 | - | cds | synonymous | 0.216713881 | 0.190026954 |
| chr21 | 46950894 | G | C | SLC19A1 | - | intron | silent | 0.001432665 | 0 |
| chr21 | 46950908 | T | C | SLC19A1 | - | intron | silent | 0 | 0.001545595 |
| chr21 | 46951319 | A | C | SLC19A1 | - | cds | synonymous | 0 | 0.001347709 |
| chr21 | 46951391 | G | A | SLC19A1 | - | cds | synonymous | 0.001410437 | 0 |
| chr21 | 46951415 | G | A | SLC19A1 | - | cds | synonymous | 0.007052186 | 0.010666667 |
| chr21 | 46951510 | C | T | SLC19A1 | - | cds | missense | 0 | 0.001329787 |
| chr21 | 46951545 | C | T | SLC19A1 | - | cds | missense | 0.001410437 | 0 |
| chr21 | 46951556 | A | G | SLC19A1 | - | cds | synonymous | 0.217207334 | 0.258916777 |
| chr21 | 46951573 | G | C | SLC19A1 | - | cds | missense | 0.001410437 | 0 |
| chr21 | 46951606 | C | A | SLC19A1 | - | cds | missense | 0 | 0.001322751 |
| chr21 | 46951638 | C | A | SLC19A1 | - | cds | missense | 0 | 0.001321004 |
| chr21 | 46951716 | C | T | SLC19A1 | - | cds | missense | 0 | 0.001321004 |
| chr21 | 46951784 | C | A | SLC19A1 | - | cds | synonymous | 0 | 0.002656042 |
| chr21 | 46951831 | A | T | SLC19A1 | - | cds | missense | 0 | 0.00265252 |
| chr21 | 46951961 | C | T | SLC19A1 | - | cds | synonymous | 0.008583691 | 0.005427408 |
| chr21 | 46952005 | C | T | SLC19A1 | - | cds | missense | 0.001412429 | 0 |
| chr21 | 46952006 | G | C | SLC19A1 | - | cds | synonymous | 0 | 0.001329787 |
| chr21 | 46952015 | C | T | SLC19A1 | - | cds | synonymous | 0.001416431 | 0 |
| chr21 | 46952094 | T | C | SLC19A1 | - | intron | silent | 0.225469729 | 0.303797468 |
| chr21 | 46952133 | G | A | SLC19A1 | - | intron | silent | 0.002590674 | 0 |
| chr21 | 46952141 | G | A | SLC19A1 | - | intron | silent | 0.010928962 | 0 |
| chr21 | 46957655 | C | T | SLC19A1 | - | intron | silent | 0.003401361 | 0 |
| chr21 | 46957750 | G | A | SLC19A1 | - | cds | missense | 0.001410437 | 0 |
| chr21 | 46957794 | T | C | SLC19A1 | - | cds | missense | 0.225669958 | 0.262879789 |
| chr21 | 46957806 | C | T | SLC19A1 | - | cds | missense | 0 | 0.001321004 |
| chr21 | 46957834 | C | G | SLC19A1 | - | cds | missense | 0 | 0.003963012 |
| chr21 | 46957873 | T | A | SLC19A1 | - | cds | missense | 0.001410437 | 0 |
| chr21 | 46957916 | G | A | SLC19A1 | - | utr5 | silent | 0.226308345 | 0.262316911 |
| chr21 | 46957930 | G | A | SLC19A1 | - | intron | silent | 0.001416431 | 0 |
| chr21 | 46957981 | C | A | SLC19A1 | - | intron | silent | 0.001492537 | 0 |
| chr21 | 46957982 | G | A | SLC19A1 | - | intron | silent | 0.217522659 | 0.257763975 |
| chr12 | 12870634 | G | C | CDKN1B | + | utr5 | silent | 0.00390625 | 0 |
| chr12 | 12870637 | G | C | CDKN1B | + | utr5 | silent | 0.003584229 | 0.014925373 |
| chr12 | 12870695 | T | C | CDKN1B | + | utr5 | silent | 0.236445783 | 0.251819505 |
| chr12 | 12870716 | G | T | CDKN1B | + | utr5 | silent | 0 | 0.001349528 |
| chr12 | 12870920 | C | T | CDKN1B | + | cds | synonymous | 0 | 0.002642008 |
| chr12 | 12870938 | G | A | CDKN1B | + | cds | synonymous | 0.060648801 | 0.052840159 |
| chr12 | 12870941 | C | T | CDKN1B | + | cds | synonymous | 0.001410437 | 0 |
| chr12 | 12870998 | G | C | CDKN1B | + | cds | missense | 0 | 0.007926024 |
| chr12 | 12871044 | C | A | CDKN1B | + | cds | missense | 0 | 0.003963012 |
| chr12 | 12871056 | C | G | CDKN1B | + | cds | missense | 0.002820874 | 0 |
| chr12 | 12871099 | T | G | CDKN1B | + | cds | missense | 0.08180536 | 0.068692206 |
| chr12 | 12871117 | C | T | CDKN1B | + | cds | missense | 0 | 0.001322751 |
| chr12 | 12871137 | C | T | CDKN1B | + | cds | missense | 0.001410437 | 0 |
| chr12 | 12871216 | G | T | CDKN1B | + | cds | missense | 0.001412429 | 0 |
| chr12 | 12871411 | T | C | CDKN1B | + | intron | silent | 0.005649718 | 0.00617284 |
| chr12 | 12871612 | C | A | CDKN1B | + | intron | silent | 0 | 0.001321004 |
| chr12 | 12871622 | A | C | CDKN1B | + | intron | silent | 0 | 0.001321004 |
| chr12 | 12871640 | A | G | CDKN1B | + | intron | silent | 0 | 0.001321004 |
| chr12 | 12871661 | G | A | CDKN1B | + | intron | silent | 0 | 0.001321004 |
| chr12 | 12871682 | T | C | CDKN1B | + | intron | silent | 0.016925247 | 0.015852048 |
| chr12 | 12871723 | T | G | CDKN1B | + | intron | silent | 0 | 0.001321004 |
| chr12 | 12871746 | C | T | CDKN1B | + | intron | silent | 0.004231312 | 0 |
| chr12 | 12872047 | C | T | CDKN1B | + | intron | silent | 0 | 0.001355014 |
| chr3 | 185766381 | A | G | ETV5 | - | utr3 | silent | 0.001410437 | 0 |
| chr3 | 185766414 | C | T | ETV5 | - | utr3 | silent | 0 | 0.001321004 |
| chr3 | 185766684 | G | A | ETV5 | - | intron | silent | 0.001410437 | 0 |
| chr3 | 185766689 | A | G | ETV5 | - | intron | silent | 0.005641749 | 0.003963012 |
| chr3 | 185766695 | T | A | ETV5 | - | intron | silent | 0.001412429 | 0 |
| chr3 | 185766698 | G | A | ETV5 | - | intron | silent | 0.001412429 | 0 |
| chr3 | 185766709 | G | C | ETV5 | - | intron | silent | 0.001410437 | 0 |
| chr3 | 185766711 | G | A | ETV5 | - | intron | silent | 0.135401975 | 0.117724868 |
| chr3 | 185766714 | C | T | ETV5 | - | intron | silent | 0.001412429 | 0 |
| chr3 | 185766744 | C | T | ETV5 | - | intron | silent | 0 | 0.001347709 |
| chr3 | 185766839 | T | C | ETV5 | - | intron | silent | 0.096579477 | 0.1 |
| chr3 | 185769685 | T | A | ETV5 | - | intron | silent | 0.01551481 | 0.009247028 |
| chr3 | 185769723 | T | G | ETV5 | - | intron | silent | 0 | 0.001321004 |
| chr3 | 185769727 | C | T | ETV5 | - | intron | silent | 0 | 0.002642008 |
| chr3 | 185769736 | G | A | ETV5 | - | intron | silent | 0 | 0.001321004 |
| chr3 | 185769812 | G | C | ETV5 | - | intron | silent | 0.001410437 | 0 |
| chr3 | 185770002 | C | T | ETV5 | - | intron | silent | 0 | 0.001321004 |
| chr3 | 185774813 | C | A | ETV5 | - | intron | silent | 0.2651622 | 0.250990753 |
| chr3 | 185775016 | G | C | ETV5 | - | cds | missense | 0 | 0.00660502 |
| chr3 | 185775180 | C | T | ETV5 | - | intron | silent | 0 | 0.001321004 |
| chr3 | 185775471 | G | A | ETV5 | - | intron | silent | 0.003960396 | 0 |
| chr3 | 185782176 | G | A | ETV5 | - | intron | silent | 0.001410437 | 0.001321004 |
| chr3 | 185782317 | A | C | ETV5 | - | intron | silent | 0 | 0.001321004 |
| chr3 | 185783449 | T | C | ETV5 | - | intron | silent | 0 | 0.001335113 |
| chr3 | 185783501 | A | G | ETV5 | - | intron | silent | 0 | 0.001321004 |
| chr3 | 185783645 | T | G | ETV5 | - | cds | synonymous | 0.001410437 | 0 |
| chr3 | 185783793 | C | T | ETV5 | - | cds | missense | 0.001410437 | 0 |
| chr3 | 185783884 | A | G | ETV5 | - | intron | silent | 0 | 0.001322751 |
| chr3 | 185783885 | T | C | ETV5 | - | intron | silent | 0.001410437 | 0 |
| chr3 | 185797905 | A | C | ETV5 | - | intron | silent | 0.001709402 | 0 |
| chr3 | 185797928 | G | C | ETV5 | - | intron | silent | 0.007648184 | 0 |
| chr3 | 185798025 | C | T | ETV5 | - | intron | silent | 0.15 | 0.06122449 |
| chr3 | 185798683 | A | T | ETV5 | - | intron | silent | 0 | 0.001321004 |
| chr3 | 185798696 | G | A | ETV5 | - | intron | silent | 0 | 0.001321004 |
| chr3 | 185798704 | T | C | ETV5 | - | intron | silent | 0 | 0.001321004 |
| chr3 | 185798719 | A | G | ETV5 | - | intron | silent | 0 | 0.001321004 |
| chr3 | 185798728 | A | G | ETV5 | - | intron | silent | 0.052186178 | 0.052840159 |
| chr3 | 185798806 | C | G | ETV5 | - | intron | silent | 0.001410437 | 0.001321004 |
| chr3 | 185799105 | C | T | ETV5 | - | intron | silent | 0.004237288 | 0.001329787 |
| chr3 | 185822963 | A | C | ETV5 | - | intron | silent | 0 | 0.001321004 |
| chr3 | 185822982 | T | C | ETV5 | - | intron | silent | 0 | 0.001321004 |
| chr3 | 185822999 | A | G | ETV5 | - | intron | silent | 0.002820874 | 0 |
| chr3 | 185823289 | A | T | ETV5 | - | intron | silent | 0.001410437 | 0 |
| chr3 | 185823392 | T | C | ETV5 | - | intron | silent | 0 | 0.001321004 |
| chr3 | 185823602 | C | A | ETV5 | - | intron | silent | 0 | 0.001321004 |
| chr3 | 185823652 | G | A | ETV5 | - | cds | synonymous | 0.001410437 | 0 |
| chr3 | 185823681 | C | T | ETV5 | - | utr5 | silent | 0.002820874 | 0.005284016 |
| chr3 | 185823795 | G | C | ETV5 | - | intron | silent | 0 | 0.00132626 |
| chr13 | 25338368 | G | A | RNF17 | + | cds | synonymous | 0.002820874 | 0 |
| chr13 | 25338378 | T | G | RNF17 | + | cds | missense | 0.008462623 | 0.001321004 |
| chr13 | 25338382 | C | T | RNF17 | + | cds | missense | 0 | 0.001321004 |
| chr13 | 25338517 | A | C | RNF17 | + | intron | silent | 0.001420455 | 0 |
| chr13 | 25338530 | A | G | RNF17 | + | intron | silent | 0 | 0.001369863 |
| chr13 | 25338601 | G | A | RNF17 | + | intron | silent | 0.001589825 | 0 |
| chr13 | 25338604 | C | T | RNF17 | + | intron | silent | 0.001620746 | 0 |
| chr13 | 25338606 | C | T | RNF17 | + | intron | silent | 0.001631321 | 0 |
| chr13 | 25341241 | C | A | RNF17 | + | intron | silent | 0 | 0.001322751 |
| chr13 | 25341266 | C | A | RNF17 | + | intron | silent | 0.001410437 | 0 |
| chr13 | 25341312 | A | C | RNF17 | + | intron | silent | 0.001412429 | 0 |
| chr13 | 25341368 | C | A | RNF17 | + | intron | silent | 0 | 0.001321004 |
| chr13 | 25341491 | G | A | RNF17 | + | cds | missense | 0 | 0.001321004 |
| chr13 | 25341515 | T | C | RNF17 | + | intron | silent | 0.001410437 | 0 |
| chr13 | 25341530 | A | G | RNF17 | + | intron | silent | 0.004231312 | 0.00660502 |
| chr13 | 25341625 | C | T | RNF17 | + | intron | silent | 0.002853067 | 0.001356852 |
| chr13 | 25348911 | T | C | RNF17 | + | intron | silent | 0 | 0.001321004 |
| chr13 | 25348973 | G | C | RNF17 | + | cds | missense | 0 | 0.001321004 |
| chr13 | 25349180 | C | T | RNF17 | + | intron | silent | 0.007102273 | 0.002656042 |
| chr13 | 25352298 | A | G | RNF17 | + | intron | silent | 0.008695652 | 0 |
| chr13 | 25352334 | A | G | RNF17 | + | intron | silent | 0.002836879 | 0 |
| chr13 | 25352475 | G | A | RNF17 | + | cds | synonymous | 0.001410437 | 0.001321004 |
| chr13 | 25352494 | C | T | RNF17 | + | cds | missense | 0.001410437 | 0 |
| chr13 | 25352579 | G | A | RNF17 | + | intron | silent | 0 | 0.002642008 |
| chr13 | 25352584 | C | T | RNF17 | + | intron | silent | 0 | 0.001321004 |
| chr13 | 25352652 | C | T | RNF17 | + | intron | silent | 0.039492243 | 0.042272127 |
| chr13 | 25352684 | C | G | RNF17 | + | intron | silent | 0 | 0.001333333 |
| chr13 | 25353619 | G | A | RNF17 | + | intron | silent | 0 | 0.001331558 |
| chr13 | 25353645 | T | G | RNF17 | + | intron | silent | 0 | 0.001324503 |
| chr13 | 25353716 | A | G | RNF17 | + | intron | silent | 0.001410437 | 0 |
| chr13 | 25353808 | A | C | RNF17 | + | cds | missense | 0 | 0.001321004 |
| chr13 | 25353899 | A | G | RNF17 | + | intron | silent | 0.001410437 | 0.001321004 |
| chr13 | 25353910 | C | G | RNF17 | + | intron | silent | 0 | 0.001321004 |
| chr13 | 25355795 | T | A | RNF17 | + | intron | silent | 0.152459016 | 0.142857143 |
| chr13 | 25355874 | G | A | RNF17 | + | intron | silent | 0.002820874 | 0 |
| chr13 | 25356053 | T | C | RNF17 | + | cds | synonymous | 0.301833568 | 0.268163804 |
| chr13 | 25356199 | A | G | RNF17 | + | intron | silent | 0 | 0.001322751 |
| chr13 | 25356210 | A | G | RNF17 | + | intron | silent | 0 | 0.001322751 |
| chr13 | 25362036 | A | G | RNF17 | + | intron | silent | 0.001430615 | 0.001324503 |
| chr13 | 25362316 | A | G | RNF17 | + | intron | silent | 0.004255319 | 0.00794702 |
| chr13 | 25362443 | G | A | RNF17 | + | intron | silent | 0.475 | 0.498652291 |
| chr13 | 25363319 | C | A | RNF17 | + | intron | silent | 0.164285714 | 0.16644474 |
| chr13 | 25363408 | T | C | RNF17 | + | intron | silent | 0 | 0.001322751 |
| chr13 | 25363583 | G | T | RNF17 | + | intron | silent | 0.001410437 | 0 |
| chr13 | 25363615 | C | T | RNF17 | + | intron | silent | 0 | 0.001322751 |
| chr13 | 25363648 | T | C | RNF17 | + | intron | silent | 0.001410437 | 0 |
| chr13 | 25363674 | C | T | RNF17 | + | intron | silent | 0 | 0.001324503 |
| chr13 | 25363747 | A | G | RNF17 | + | intron | silent | 0.001414427 | 0 |
| chr13 | 25363776 | C | T | RNF17 | + | intron | silent | 0.008462623 | 0 |
| chr13 | 25364037 | A | G | RNF17 | + | intron | silent | 0 | 0.001328021 |
| chr13 | 25366994 | A | G | RNF17 | + | intron | silent | 0.001766784 | 0 |
| chr13 | 25367113 | G | A | RNF17 | + | intron | silent | 0 | 0.002642008 |
| chr13 | 25367124 | A | T | RNF17 | + | intron | silent | 0 | 0.001321004 |
| chr13 | 25367269 | C | T | RNF17 | + | cds | missense | 0 | 0.001321004 |
| chr13 | 25367282 | A | C | RNF17 | + | cds | missense | 0 | 0.002642008 |
| chr13 | 25367301 | C | T | RNF17 | + | cds | synonymous | 0.396332863 | 0.348745046 |
| chr13 | 25367324 | T | A | RNF17 | + | cds | synonymous | 0.162200282 | 0.142668428 |
| chr13 | 25367325 | C | T | RNF17 | + | cds | missense | 0.162200282 | 0.142668428 |
| chr13 | 25367388 | G | A | RNF17 | + | cds | missense | 0 | 0.001321004 |
| chr13 | 25367414 | T | G | RNF17 | + | cds | missense | 0 | 0.002642008 |
| chr13 | 25367555 | G | A | RNF17 | + | intron | silent | 0 | 0.001321004 |
| chr13 | 25367629 | A | G | RNF17 | + | intron | silent | 0 | 0.001388889 |
| chr13 | 25370147 | G | A | RNF17 | + | intron | silent | 0.001412429 | 0 |
| chr13 | 25370202 | T | G | RNF17 | + | intron | silent | 0.001410437 | 0 |
| chr13 | 25370209 | G | A | RNF17 | + | intron | silent | 0 | 0.001321004 |
| chr13 | 25370233 | T | C | RNF17 | + | intron | silent | 0 | 0.001321004 |
| chr13 | 25370255 | A | G | RNF17 | + | intron | silent | 0.022566996 | 0.017173052 |
| chr13 | 25370355 | G | A | RNF17 | + | cds | missense | 0.001410437 | 0 |
| chr13 | 25370521 | T | C | RNF17 | + | intron | silent | 0 | 0.001324503 |
| chr13 | 25370624 | C | T | RNF17 | + | intron | silent | 0.096153846 | 0.099688474 |
| chr13 | 25373384 | A | G | RNF17 | + | intron | silent | 0.001428571 | 0 |
| chr13 | 25373411 | G | A | RNF17 | + | intron | silent | 0 | 0.001324503 |
| chr13 | 25373432 | C | T | RNF17 | + | intron | silent | 0.213578501 | 0.19205298 |
| chr13 | 25373462 | C | T | RNF17 | + | intron | silent | 0.001414427 | 0 |
| chr13 | 25373478 | T | C | RNF17 | + | intron | silent | 0.001412429 | 0 |
| chr13 | 25373515 | G | C | RNF17 | + | intron | silent | 0.427362482 | 0.454425363 |
| chr13 | 25373604 | A | G | RNF17 | + | cds | missense | 0 | 0.001321004 |
| chr13 | 25373632 | G | C | RNF17 | + | cds | missense | 0 | 0.001322751 |
| chr13 | 25373719 | C | T | RNF17 | + | cds | missense | 0.001412429 | 0 |
| chr13 | 25373729 | T | C | RNF17 | + | intron | silent | 0 | 0.001324503 |
| chr13 | 25373758 | T | G | RNF17 | + | intron | silent | 0 | 0.001324503 |
| chr13 | 25374398 | T | G | RNF17 | + | intron | silent | 0 | 0.001322751 |
| chr13 | 25374419 | C | T | RNF17 | + | intron | silent | 0.007052186 | 0.014531044 |
| chr13 | 25374555 | A | G | RNF17 | + | cds | synonymous | 0.001410437 | 0 |
| chr13 | 25374707 | G | A | RNF17 | + | intron | silent | 0.179125529 | 0.175693527 |
| chr13 | 25374838 | C | T | RNF17 | + | intron | silent | 0.170015456 | 0.142857143 |
| chr13 | 25376420 | C | T | RNF17 | + | intron | silent | 0.296453901 | 0.266578249 |
| chr13 | 25376657 | A | G | RNF17 | + | cds | missense | 0.002820874 | 0.001321004 |
| chr13 | 25376834 | T | C | RNF17 | + | intron | silent | 0 | 0.001331558 |
| chr13 | 25378246 | A | C | RNF17 | + | intron | silent | 0.029126214 | 0.045655376 |
| chr13 | 25378388 | G | A | RNF17 | + | intron | silent | 0.001410437 | 0 |
| chr13 | 25378401 | C | T | RNF17 | + | intron | silent | 0.001410437 | 0 |
| chr13 | 25378476 | A | G | RNF17 | + | cds | missense | 0.483779972 | 0.498018494 |
| chr13 | 25378584 | T | C | RNF17 | + | intron | silent | 0.001410437 | 0 |
| chr13 | 25378669 | C | A | RNF17 | + | intron | silent | 0 | 0.001324503 |
| chr13 | 25378759 | A | G | RNF17 | + | intron | silent | 0.178502879 | 0.16744186 |
| chr13 | 25399558 | T | C | RNF17 | + | intron | silent | 0 | 0.002739726 |
| chr13 | 25399677 | A | G | RNF17 | + | intron | silent | 0.394067797 | 0.345695364 |
| chr13 | 25399702 | C | A | RNF17 | + | intron | silent | 0.166431594 | 0.165125495 |
| chr13 | 25399752 | A | G | RNF17 | + | intron | silent | 0.022566996 | 0.017173052 |
| chr13 | 25399782 | T | C | RNF17 | + | cds | missense | 0.001410437 | 0 |
| chr13 | 25399838 | C | T | RNF17 | + | cds | missense | 0.001410437 | 0 |
| chr13 | 25399961 | A | G | RNF17 | + | intron | silent | 0 | 0.001321004 |
| chr13 | 25400038 | T | A | RNF17 | + | intron | silent | 0 | 0.00132626 |
| chr13 | 25404438 | A | G | RNF17 | + | intron | silent | 0.001545595 | 0 |
| chr13 | 25404454 | G | A | RNF17 | + | intron | silent | 0 | 0.002659574 |
| chr13 | 25404743 | T | C | RNF17 | + | intron | silent | 0.005641749 | 0 |
| chr13 | 25404886 | A | G | RNF17 | + | intron | silent | 0.004431315 | 0 |
| chr13 | 25404909 | A | G | RNF17 | + | intron | silent | 0.157977883 | 0.144645341 |
| chr13 | 25404922 | G | A | RNF17 | + | intron | silent | 0.001766784 | 0.001477105 |
| chr13 | 25405853 | A | G | RNF17 | + | intron | silent | 0.164244186 | 0.166002656 |
| chr13 | 25406073 | T | G | RNF17 | + | cds | missense | 0 | 0.001324503 |
| chr13 | 25406230 | G | C | RNF17 | + | intron | silent | 0 | 0.001328021 |
| chr13 | 25406232 | T | G | RNF17 | + | intron | silent | 0.001457726 | 0 |
| chr13 | 25406257 | A | G | RNF17 | + | intron | silent | 0.159874608 | 0.146540027 |
| chr13 | 25416152 | C | A | RNF17 | + | intron | silent | 0 | 0.001321004 |
| chr13 | 25416194 | A | G | RNF17 | + | cds | missense | 0.002820874 | 0.00660502 |
| chr13 | 25416331 | T | C | RNF17 | + | intron | silent | 0.001410437 | 0 |
| chr13 | 25416343 | G | T | RNF17 | + | intron | silent | 0.001410437 | 0 |
| chr13 | 25416445 | A | G | RNF17 | + | intron | silent | 0.002828854 | 0.001340483 |
| chr13 | 25417797 | T | G | RNF17 | + | intron | silent | 0.043478261 | 0.08 |
| chr13 | 25417869 | T | C | RNF17 | + | intron | silent | 0 | 0.001324503 |
| chr13 | 25418033 | C | T | RNF17 | + | cds | missense | 0.001414427 | 0 |
| chr13 | 25418050 | T | C | RNF17 | + | cds | synonymous | 0 | 0.002649007 |
| chr13 | 25418128 | A | G | RNF17 | + | intron | silent | 0.002886003 | 0.00265252 |
| chr13 | 25418289 | G | T | RNF17 | + | intron | silent | 0.014814815 | 0.025575448 |
| chr13 | 25418652 | T | C | RNF17 | + | intron | silent | 0.164444444 | 0.142857143 |
| chr13 | 25418699 | C | T | RNF17 | + | intron | silent | 0.001414427 | 0 |
| chr13 | 25418848 | G | A | RNF17 | + | cds | missense | 0 | 0.001321004 |
| chr13 | 25419176 | A | G | RNF17 | + | cds | synonymous | 0.023977433 | 0.017195767 |
| chr13 | 25419273 | C | T | RNF17 | + | intron | silent | 0.001426534 | 0 |
| chr13 | 25419274 | G | A | RNF17 | + | intron | silent | 0.001426534 | 0 |
| chr13 | 25424369 | A | G | RNF17 | + | intron | silent | 0.483779972 | 0.498677249 |
| chr13 | 25424389 | G | A | RNF17 | + | intron | silent | 0 | 0.001321004 |
| chr13 | 25424571 | T | A | RNF17 | + | cds | missense | 0.001410437 | 0 |
| chr13 | 25424583 | T | C | RNF17 | + | intron | silent | 0.162200282 | 0.142668428 |
| chr13 | 25424651 | C | G | RNF17 | + | intron | silent | 0.001410437 | 0 |
| chr13 | 25425411 | C | T | RNF17 | + | intron | silent | 0.003012048 | 0 |
| chr13 | 25425564 | C | T | RNF17 | + | intron | silent | 0.018335684 | 0.018494055 |
| chr13 | 25425684 | A | G | RNF17 | + | cds | missense | 0 | 0.001321004 |
| chr13 | 25425723 | T | A | RNF17 | + | intron | silent | 0.180535966 | 0.178335535 |
| chr13 | 25425729 | A | T | RNF17 | + | intron | silent | 0 | 0.001321004 |
| chr13 | 25427796 | A | G | RNF17 | + | intron | silent | 0.002262443 | 0 |
| chr13 | 25427855 | A | T | RNF17 | + | intron | silent | 0 | 0.001331558 |
| chr13 | 25427912 | C | T | RNF17 | + | intron | silent | 0.001416431 | 0 |
| chr13 | 25427928 | A | G | RNF17 | + | intron | silent | 0.001412429 | 0 |
| chr13 | 25427983 | G | C | RNF17 | + | intron | silent | 0.001410437 | 0 |
| chr13 | 25428002 | C | A | RNF17 | + | cds | missense | 0.33145275 | 0.310435931 |
| chr13 | 25428173 | A | T | RNF17 | + | cds | missense | 0.019746121 | 0.023778071 |
| chr13 | 25428215 | C | T | RNF17 | + | cds | synonymous | 0 | 0.001321004 |
| chr13 | 25428354 | C | T | RNF17 | + | intron | silent | 0.001410437 | 0 |
| chr13 | 25428364 | C | T | RNF17 | + | intron | silent | 0.001410437 | 0.001321004 |
| chr13 | 25428365 | G | A | RNF17 | + | intron | silent | 0.179378531 | 0.175693527 |
| chr13 | 25428379 | G | T | RNF17 | + | intron | silent | 0.00141844 | 0 |
| chr13 | 25428413 | A | G | RNF17 | + | intron | silent | 0.002932551 | 0.001329787 |
| chr13 | 25433118 | A | G | RNF17 | + | intron | silent | 0.162200282 | 0.142668428 |
| chr13 | 25433204 | C | A | RNF17 | + | cds | missense | 0.078984485 | 0.068692206 |
| chr13 | 25433495 | A | T | RNF17 | + | intron | silent | 0 | 0.001831502 |
| chr13 | 25435378 | A | T | RNF17 | + | intron | silent | 0.023977433 | 0.025099075 |
| chr13 | 25435569 | G | A | RNF17 | + | intron | silent | 0 | 0.001322751 |
| chr13 | 25436663 | G | A | RNF17 | + | intron | silent | 0.001472754 | 0 |
| chr13 | 25436687 | G | A | RNF17 | + | intron | silent | 0.001422475 | 0 |
| chr13 | 25436707 | T | C | RNF17 | + | intron | silent | 0 | 0.001324503 |
| chr13 | 25436793 | C | T | RNF17 | + | intron | silent | 0 | 0.001324503 |
| chr13 | 25436879 | T | C | RNF17 | + | cds | missense | 0 | 0.001321004 |
| chr13 | 25437068 | T | A | RNF17 | + | intron | silent | 0.001438849 | 0 |
| chr13 | 25438851 | G | A | RNF17 | + | intron | silent | 0.001438849 | 0 |
| chr13 | 25438877 | T | A | RNF17 | + | intron | silent | 0.178062678 | 0.175925926 |
| chr13 | 25439126 | T | C | RNF17 | + | cds | missense | 0 | 0.001324503 |
| chr13 | 25439207 | A | T | RNF17 | + | intron | silent | 0 | 0.001321004 |
| chr13 | 25439208 | C | T | RNF17 | + | intron | silent | 0 | 0.001322751 |
| chr13 | 25439256 | T | C | RNF17 | + | intron | silent | 0 | 0.001329787 |
| chr13 | 25440237 | A | G | RNF17 | + | intron | silent | 0.029619182 | 0.03042328 |
| chr13 | 25440318 | G | A | RNF17 | + | cds | missense | 0.497884344 | 0.486092715 |
| chr13 | 25440356 | A | G | RNF17 | + | intron | silent | 0 | 0.002649007 |
| chr13 | 25440423 | T | G | RNF17 | + | intron | silent | 0 | 0.001324503 |
| chr13 | 25440496 | T | C | RNF17 | + | intron | silent | 0.495974235 | 0.479432624 |
| chr13 | 25442790 | C | T | RNF17 | + | cds | missense | 0 | 0.001321004 |
| chr13 | 25442891 | G | A | RNF17 | + | intron | silent | 0 | 0.001321004 |
| chr13 | 25442921 | A | C | RNF17 | + | intron | silent | 0.203102962 | 0.182298547 |
| chr13 | 25442980 | G | A | RNF17 | + | intron | silent | 0.001412429 | 0 |
| chr13 | 25443029 | G | A | RNF17 | + | intron | silent | 0.001464129 | 0 |
| chr13 | 25444543 | A | C | RNF17 | + | intron | silent | 0.179487179 | 0.176392573 |
| chr13 | 25444616 | G | A | RNF17 | + | intron | silent | 0.001410437 | 0.003963012 |
| chr13 | 25444818 | C | T | RNF17 | + | cds | missense | 0 | 0.001321004 |
| chr13 | 25444863 | C | T | RNF17 | + | cds | missense | 0.001410437 | 0 |
| chr13 | 25444934 | C | T | RNF17 | + | intron | silent | 0.497884344 | 0.485449735 |
| chr13 | 25445026 | A | G | RNF17 | + | intron | silent | 0.002923977 | 0 |
| chr13 | 25445076 | C | T | RNF17 | + | intron | silent | 0 | 0.003030303 |
| chr13 | 25448099 | A | G | RNF17 | + | intron | silent | 0.004297994 | 0.00795756 |
| chr13 | 25448120 | G | A | RNF17 | + | intron | silent | 0.009915014 | 0.00397351 |
| chr13 | 25448136 | G | A | RNF17 | + | intron | silent | 0.002820874 | 0.01321004 |
| chr13 | 25448142 | T | C | RNF17 | + | intron | silent | 0.004237288 | 0 |
| chr13 | 25448347 | T | C | RNF17 | + | cds | missense | 0 | 0.002642008 |
| chr13 | 25448434 | A | G | RNF17 | + | intron | silent | 0 | 0.001322751 |
| chr13 | 25448465 | C | A | RNF17 | + | intron | silent | 0.001422475 | 0 |
| chr13 | 25451053 | G | C | RNF17 | + | intron | silent | 0.002820874 | 0 |
| chr13 | 25451097 | G | A | RNF17 | + | intron | silent | 0.49506347 | 0.48348745 |
| chr13 | 25451375 | A | G | RNF17 | + | intron | silent | 0.162200282 | 0.142668428 |
| chr13 | 25451414 | G | T | RNF17 | + | intron | silent | 0.008474576 | 0.001321004 |
| chr13 | 25451482 | A | G | RNF17 | + | intron | silent | 0.001821494 | 0 |
| chr13 | 25453187 | C | G | RNF17 | + | intron | silent | 0 | 0.001321004 |
| chr13 | 25453221 | C | T | RNF17 | + | intron | silent | 0.011283498 | 0.021136063 |
| chr13 | 25453235 | T | A | RNF17 | + | intron | silent | 0.004231312 | 0.003963012 |
| chr13 | 25453387 | G | A | RNF17 | + | cds | synonymous | 0.001410437 | 0 |
| chr13 | 25453420 | A | G | RNF17 | + | cds | synonymous | 0.162200282 | 0.142668428 |
| chr13 | 25453424 | G | A | RNF17 | + | utr3 | silent | 0 | 0.001321004 |
| chr13 | 25453509 | C | T | RNF17 | + | intron | silent | 0.162200282 | 0.142668428 |
| chr13 | 25453528 | C | A | RNF17 | + | intron | silent | 0 | 0.001322751 |
| chr13 | 25453547 | C | T | RNF17 | + | intron | silent | 0.002820874 | 0.00397878 |
| chr20 | 31367823 | G | A | DNMT3B | + | utr5 | silent | 0.001416431 | 0 |
| chr20 | 31367858 | C | G | DNMT3B | + | utr5 | silent | 0.005641749 | 0.005284016 |
| chr20 | 31367893 | C | T | DNMT3B | + | utr5 | silent | 0 | 0.003963012 |
| chr20 | 31367948 | C | T | DNMT3B | + | cds | synonymous | 0.111424542 | 0.136063408 |
| chr20 | 31367957 | G | T | DNMT3B | + | intron | silent | 0 | 0.001321004 |
| chr20 | 31368013 | G | T | DNMT3B | + | intron | silent | 0 | 0.001322751 |
| chr20 | 31368057 | G | A | DNMT3B | + | intron | silent | 0 | 0.001322751 |
| chr20 | 31368065 | A | G | DNMT3B | + | intron | silent | 0 | 0.001321004 |
| chr20 | 31368126 | A | G | DNMT3B | + | cds | synonymous | 0.001410437 | 0 |
| chr20 | 31368139 | G | C | DNMT3B | + | cds | missense | 0.001410437 | 0 |
| chr20 | 31368168 | C | T | DNMT3B | + | cds | synonymous | 0 | 0.001321004 |
| chr20 | 31368171 | C | T | DNMT3B | + | cds | synonymous | 0.014104372 | 0.00660502 |
| chr20 | 31368196 | G | A | DNMT3B | + | cds | missense | 0 | 0.001321004 |
| chr20 | 31368321 | C | T | DNMT3B | + | intron | silent | 0 | 0.003968254 |
| chr20 | 31368351 | G | C | DNMT3B | + | intron | silent | 0.009929078 | 0.005326232 |
| chr20 | 31368380 | C | A | DNMT3B | + | intron | silent | 0 | 0.00136612 |
| chr20 | 31368384 | G | A | DNMT3B | + | intron | silent | 0.001440922 | 0 |
| chr20 | 31368960 | C | A | DNMT3B | + | intron | silent | 0.002928258 | 0.002890173 |
| chr20 | 31369084 | G | C | DNMT3B | + | intron | silent | 0.001410437 | 0 |
| chr20 | 31369088 | C | T | DNMT3B | + | intron | silent | 0 | 0.001321004 |
| chr20 | 31369120 | T | G | DNMT3B | + | intron | silent | 0.001410437 | 0 |
| chr20 | 31369198 | C | T | DNMT3B | + | cds | missense | 0 | 0.001321004 |
| chr20 | 31369280 | G | A | DNMT3B | + | intron | silent | 0.001410437 | 0 |
| chr20 | 31372437 | T | C | DNMT3B | + | intron | silent | 0 | 0.001321004 |
| chr20 | 31372484 | T | C | DNMT3B | + | intron | silent | 0.001410437 | 0 |
| chr20 | 31372494 | A | G | DNMT3B | + | intron | silent | 0 | 0.001321004 |
| chr20 | 31372520 | A | C | DNMT3B | + | intron | silent | 0.001410437 | 0 |
| chr20 | 31372582 | G | A | DNMT3B | + | cds | missense | 0 | 0.001321004 |
| chr20 | 31372592 | A | C | DNMT3B | + | cds | missense | 0 | 0.001321004 |
| chr20 | 31372649 | G | A | DNMT3B | + | cds | missense | 0.001410437 | 0 |
| chr20 | 31372697 | G | T | DNMT3B | + | intron | silent | 0.002820874 | 0 |
| chr20 | 31372746 | G | C | DNMT3B | + | intron | silent | 0.002820874 | 0.001321004 |
| chr20 | 31374143 | G | T | DNMT3B | + | intron | silent | 0.004273504 | 0 |
| chr20 | 31374166 | C | T | DNMT3B | + | intron | silent | 0.110481586 | 0.119047619 |
| chr20 | 31374222 | C | T | DNMT3B | + | intron | silent | 0.001410437 | 0.001321004 |
| chr20 | 31374250 | G | A | DNMT3B | + | intron | silent | 0 | 0.001321004 |
| chr20 | 31374259 | C | T | DNMT3B | + | intron | silent | 0 | 0 |
| chr20 | 31374293 | C | G | DNMT3B | + | intron | silent | 0 | 0.002642008 |
| chr20 | 31374318 | G | A | DNMT3B | + | cds | missense | 0 | 0.001321004 |
| chr20 | 31374342 | G | A | DNMT3B | + | cds | missense | 0 | 0.001321004 |
| chr20 | 31374365 | C | T | DNMT3B | + | cds | missense | 0.001410437 | 0 |
| chr20 | 31374380 | C | T | DNMT3B | + | cds | missense | 0 | 0.001321004 |
| chr20 | 31374390 | G | A | DNMT3B | + | cds | missense | 0.001410437 | 0 |
| chr20 | 31374418 | G | C | DNMT3B | + | cds | missense | 0 | 0.001321004 |
| chr20 | 31374520 | A | G | DNMT3B | + | intron | silent | 0 | 0 |
| chr20 | 31374594 | G | C | DNMT3B | + | intron | silent | 0 | 0.00140056 |
| chr20 | 31374908 | G | A | DNMT3B | + | intron | silent | 0.001410437 | 0 |
| chr20 | 31374991 | G | A | DNMT3B | + | intron | silent | 0.002820874 | 0.003963012 |
| chr20 | 31375007 | G | A | DNMT3B | + | intron | silent | 0.001410437 | 0.003963012 |
| chr20 | 31375019 | T | C | DNMT3B | + | intron | silent | 0 | 0.001321004 |
| chr20 | 31375064 | C | T | DNMT3B | + | cds | missense | 0.001410437 | 0 |
| chr20 | 31375087 | C | G | DNMT3B | + | cds | missense | 0.001410437 | 0 |
| chr20 | 31375195 | G | T | DNMT3B | + | cds | missense | 0.008462623 | 0.002642008 |
| chr20 | 31375286 | G | T | DNMT3B | + | intron | silent | 0.001410437 | 0 |
| chr20 | 31375311 | A | G | DNMT3B | + | intron | silent | 0.002820874 | 0.003963012 |
| chr20 | 31375345 | C | T | DNMT3B | + | intron | silent | 0.001410437 | 0 |
| chr20 | 31375386 | A | G | DNMT3B | + | intron | silent | 0.003012048 | 0 |
| chr20 | 31375416 | A | C | DNMT3B | + | intron | silent | 0.004132231 | 0 |
| chr20 | 31375417 | C | T | DNMT3B | + | intron | silent | 0.003992016 | 0 |
| chr20 | 31376490 | T | C | DNMT3B | + | intron | silent | 0 | 0 |
| chr20 | 31376496 | G | A | DNMT3B | + | intron | silent | 0 | 0.00140647 |
| chr20 | 31376643 | T | C | DNMT3B | + | intron | silent | 0.002820874 | 0.001321004 |
| chr20 | 31376707 | G | A | DNMT3B | + | cds | synonymous | 0 | 0.001321004 |
| chr20 | 31376800 | C | T | DNMT3B | + | cds | synonymous | 0.004231312 | 0 |
| chr20 | 31376995 | C | T | DNMT3B | + | intron | silent | 0.003189793 | 0.001663894 |
| chr20 | 31376999 | G | A | DNMT3B | + | intron | silent | 0 | 0.001769912 |
| chr20 | 31379425 | G | A | DNMT3B | + | cds | missense | 0 | 0.001321004 |
| chr20 | 31379479 | G | A | DNMT3B | + | cds | missense | 0.002820874 | 0 |
| chr20 | 31379529 | G | A | DNMT3B | + | intron | silent | 0.001410437 | 0 |
| chr20 | 31379665 | C | T | DNMT3B | + | intron | silent | 0.002898551 | 0.004213483 |
| chr20 | 31380309 | G | A | DNMT3B | + | intron | silent | 0.002853067 | 0.004 |
| chr20 | 31380436 | C | T | DNMT3B | + | cds | missense | 0.001410437 | 0 |
| chr20 | 31380483 | T | C | DNMT3B | + | cds | synonymous | 0.001410437 | 0 |
| chr20 | 31380521 | C | T | DNMT3B | + | cds | synonymous | 0 | 0.001321004 |
| chr20 | 31380569 | G | A | DNMT3B | + | cds | synonymous | 0.001410437 | 0 |
| chr20 | 31380716 | A | G | DNMT3B | + | intron | silent | 0 | 0.001531394 |
| chr20 | 31380727 | G | T | DNMT3B | + | intron | silent | 0.001769912 | 0 |
| chr20 | 31380763 | C | T | DNMT3B | + | intron | silent | 0.004694836 | 0 |
| chr20 | 31381229 | G | A | DNMT3B | + | intron | silent | 0 | 0 |
| chr20 | 31381239 | A | G | DNMT3B | + | intron | silent | 0 | 0.00132626 |
| chr20 | 31381240 | T | A | DNMT3B | + | intron | silent | 0.001414427 | 0 |
| chr20 | 31381354 | C | T | DNMT3B | + | cds | missense | 0 | 0.001321004 |
| chr20 | 31381365 | C | T | DNMT3B | + | cds | missense | 0.001410437 | 0 |
| chr20 | 31381413 | C | A | DNMT3B | + | intron | silent | 0.001410437 | 0 |
| chr20 | 31381416 | T | C | DNMT3B | + | intron | silent | 0 | 0.005284016 |
| chr20 | 31381429 | C | T | DNMT3B | + | intron | silent | 0.001410437 | 0.001321004 |
| chr20 | 31383299 | A | G | DNMT3B | + | cds | missense | 0 | 0.001321004 |
| chr20 | 31383300 | T | C | DNMT3B | + | cds | synonymous | 0.001410437 | 0 |
| chr20 | 31383303 | C | T | DNMT3B | + | cds | synonymous | 0 | 0.003963012 |
| chr20 | 31383317 | G | A | DNMT3B | + | cds | missense | 0 | 0.001321004 |
| chr20 | 31383353 | T | G | DNMT3B | + | intron | silent | 0.002820874 | 0.003963012 |
| chr20 | 31383413 | C | G | DNMT3B | + | intron | silent | 0 | 0.001321004 |
| chr20 | 31383414 | G | A | DNMT3B | + | intron | silent | 0 | 0.001321004 |
| chr20 | 31383506 | G | A | DNMT3B | + | intron | silent | 0.002820874 | 0.001321004 |
| chr20 | 31383530 | C | T | DNMT3B | + | intron | silent | 0.002820874 | 0.003963012 |
| chr20 | 31384481 | G | A | DNMT3B | + | intron | silent | 0.001410437 | 0 |
| chr20 | 31384694 | G | A | DNMT3B | + | intron | silent | 0 | 0.002642008 |
| chr20 | 31384753 | T | C | DNMT3B | + | intron | silent | 0.001410437 | 0 |
| chr20 | 31384801 | C | T | DNMT3B | + | intron | silent | 0.002820874 | 0.001321004 |
| chr20 | 31384880 | C | A | DNMT3B | + | intron | silent | 0.002824859 | 0.001324503 |
| chr20 | 31384887 | C | T | DNMT3B | + | intron | silent | 0 | 0.001321004 |
| chr20 | 31384898 | T | C | DNMT3B | + | intron | silent | 0.001410437 | 0 |
| chr20 | 31384947 | C | G | DNMT3B | + | intron | silent | 0 | 0.001321004 |
| chr20 | 31385122 | G | A | DNMT3B | + | intron | silent | 0 | 0.001321004 |
| chr20 | 31385142 | C | G | DNMT3B | + | intron | silent | 0.001410437 | 0.002642008 |
| chr20 | 31385149 | G | A | DNMT3B | + | intron | silent | 0 | 0.001321004 |
| chr20 | 31385154 | C | G | DNMT3B | + | intron | silent | 0 | 0.001321004 |
| chr20 | 31385174 | G | A | DNMT3B | + | intron | silent | 0.001410437 | 0 |
| chr20 | 31385269 | G | T | DNMT3B | + | intron | silent | 0.002877698 | 0.00273224 |
| chr20 | 31386128 | C | T | DNMT3B | + | intron | silent | 0.001485884 | 0 |
| chr20 | 31386291 | G | T | DNMT3B | + | cds | missense | 0 | 0.001321004 |
| chr20 | 31386347 | T | C | DNMT3B | + | cds | synonymous | 0 | 0 |
| chr20 | 31386366 | C | T | DNMT3B | + | cds | missense | 0 | 0.001321004 |
| chr20 | 31386367 | G | A | DNMT3B | + | cds | missense | 0 | 0.001321004 |
| chr20 | 31386377 | C | T | DNMT3B | + | cds | synonymous | 0.001410437 | 0.001321004 |
| chr20 | 31386378 | G | A | DNMT3B | + | cds | missense | 0 | 0.003963012 |
| chr20 | 31386385 | G | A | DNMT3B | + | cds | missense | 0.001410437 | 0.002642008 |
| chr20 | 31386449 | T | C | DNMT3B | + | cds | synonymous | 0 | 0 |
| chr20 | 31386466 | C | T | DNMT3B | + | intron | silent | 0.002820874 | 0 |
| chr20 | 31386501 | G | A | DNMT3B | + | intron | silent | 0.001410437 | 0 |
| chr20 | 31386558 | C | A | DNMT3B | + | intron | silent | 0.001436782 | 0.001358696 |
| chr20 | 31386608 | C | T | DNMT3B | + | intron | silent | 0.111111111 | 0.135464231 |
| chr20 | 31386620 | G | A | DNMT3B | + | intron | silent | 0 | 0.006677796 |
| chr20 | 31386858 | C | T | DNMT3B | + | intron | silent | 0.001440922 | 0 |
| chr20 | 31386875 | C | T | DNMT3B | + | intron | silent | 0 | 0.002684564 |
| chr20 | 31387130 | G | A | DNMT3B | + | cds | synonymous | 0.001410437 | 0 |
| chr20 | 31387142 | C | T | DNMT3B | + | intron | silent | 0 | 0.001321004 |
| chr20 | 31387185 | G | A | DNMT3B | + | intron | silent | 0.002820874 | 0.00660502 |
| chr20 | 31387230 | A | T | DNMT3B | + | intron | silent | 0.023977433 | 0.021136063 |
| chr20 | 31387323 | G | A | DNMT3B | + | intron | silent | 0.00297619 | 0.002754821 |
| chr20 | 31387810 | A | T | DNMT3B | + | intron | silent | 0 | 0.002642008 |
| chr20 | 31387820 | C | T | DNMT3B | + | intron | silent | 0 | 0.002645503 |
| chr20 | 31387834 | G | T | DNMT3B | + | intron | silent | 0.002820874 | 0.00660502 |
| chr20 | 31387954 | C | G | DNMT3B | + | intron | silent | 0 | 0 |
| chr20 | 31388003 | G | A | DNMT3B | + | cds | missense | 0.004231312 | 0.007926024 |
| chr20 | 31388050 | G | A | DNMT3B | + | cds | synonymous | 0 | 0.001321004 |
| chr20 | 31388168 | C | A | DNMT3B | + | intron | silent | 0.009873061 | 0.022457067 |
| chr20 | 31388229 | G | T | DNMT3B | + | intron | silent | 0.001410437 | 0 |
| chr20 | 31388265 | T | C | DNMT3B | + | intron | silent | 0.001451379 | 0 |
| chr20 | 31388461 | C | T | DNMT3B | + | intron | silent | 0 | 0 |
| chr20 | 31388517 | G | A | DNMT3B | + | intron | silent | 0.001410437 | 0 |
| chr20 | 31388552 | C | G | DNMT3B | + | intron | silent | 0 | 0.001321004 |
| chr20 | 31388636 | T | C | DNMT3B | + | intron | silent | 0 | 0 |
| chr20 | 31388691 | C | T | DNMT3B | + | cds | synonymous | 0.001410437 | 0 |
| chr20 | 31388718 | G | A | DNMT3B | + | cds | synonymous | 0 | 0.001321004 |
| chr20 | 31388829 | A | T | DNMT3B | + | intron | silent | 0 | 0.001321004 |
| chr20 | 31388830 | A | G | DNMT3B | + | intron | silent | 0 | 0.001321004 |
| chr20 | 31388834 | A | G | DNMT3B | + | intron | silent | 0 | 0.001321004 |
| chr20 | 31388939 | C | G | DNMT3B | + | intron | silent | 0.002820874 | 0.002642008 |
| chr20 | 31389009 | C | T | DNMT3B | + | intron | silent | 0 | 0 |
| chr20 | 31389283 | C | T | DNMT3B | + | intron | silent | 0.002820874 | 0.002642008 |
| chr20 | 31389321 | T | G | DNMT3B | + | intron | silent | 0.001410437 | 0 |
| chr20 | 31389336 | C | T | DNMT3B | + | intron | silent | 0 | 0.001322751 |
| chr20 | 31389337 | G | A | DNMT3B | + | intron | silent | 0.001410437 | 0 |
| chr20 | 31389380 | T | G | DNMT3B | + | intron | silent | 0.001416431 | 0 |
| chr20 | 31390085 | C | T | DNMT3B | + | intron | silent | 0.001410437 | 0 |
| chr20 | 31390097 | A | T | DNMT3B | + | intron | silent | 0.005641749 | 0.001321004 |
| chr20 | 31390138 | A | G | DNMT3B | + | intron | silent | 0.001410437 | 0 |
| chr20 | 31390207 | T | C | DNMT3B | + | cds | missense | 0 | 0.001321004 |
| chr20 | 31390284 | A | T | DNMT3B | + | intron | silent | 0 | 0.001321004 |
| chr20 | 31393267 | G | A | DNMT3B | + | intron | silent | 0.002820874 | 0.002642008 |
| chr20 | 31393304 | C | T | DNMT3B | + | intron | silent | 0.001410437 | 0 |
| chr20 | 31393314 | C | A | DNMT3B | + | intron | silent | 0 | 0.001322751 |
| chr20 | 31393317 | G | A | DNMT3B | + | intron | silent | 0.001412429 | 0.001322751 |
| chr20 | 31393352 | G | A | DNMT3B | + | intron | silent | 0.002836879 | 0.00397878 |
| chr20 | 31393834 | C | T | DNMT3B | + | intron | silent | 0 | 0.001410437 |
| chr20 | 31393913 | G | T | DNMT3B | + | intron | silent | 0.001410437 | 0 |
| chr20 | 31393955 | C | A | DNMT3B | + | intron | silent | 0.001410437 | 0 |
| chr20 | 31394192 | C | T | DNMT3B | + | intron | silent | 0 | 0.001321004 |
| chr20 | 31394232 | C | T | DNMT3B | + | intron | silent | 0 | 0.001321004 |
| chr20 | 31394251 | C | T | DNMT3B | + | intron | silent | 0 | 0.001321004 |
| chr20 | 31395376 | C | T | DNMT3B | + | intron | silent | 0.00295858 | 0 |
| chr20 | 31395477 | G | A | DNMT3B | + | intron | silent | 0.002820874 | 0.001321004 |
| chr20 | 31395540 | T | C | DNMT3B | + | intron | silent | 0 | 0.001321004 |
| chr20 | 31395624 | G | A | DNMT3B | + | cds | missense | 0 | 0.001321004 |
| chr20 | 31395729 | C | T | DNMT3B | + | utr3 | silent | 0 | 0.001321004 |
| chr20 | 31395822 | G | A | DNMT3B | + | utr3 | silent | 0 | 0.001351351 |
| chr20 | 31395846 | A | G | DNMT3B | + | utr3 | silent | 0.001445087 | 0 |
| chr4 | 70707626 | T | C | SULT1E1 | - | utr3 | silent | 0 | 0.001324503 |
| chr4 | 70707721 | A | G | SULT1E1 | - | cds | synonymous | 0 | 0.001324503 |
| chr4 | 70707873 | T | A | SULT1E1 | - | intron | silent | 0.001410437 | 0 |
| chr4 | 70707906 | A | C | SULT1E1 | - | intron | silent | 0.001412429 | 0 |
| chr4 | 70707945 | A | G | SULT1E1 | - | intron | silent | 0.220170455 | 0.179282869 |
| chr4 | 70709782 | G | A | SULT1E1 | - | intron | silent | 0.004231312 | 0.003963012 |
| chr4 | 70709826 | C | A | SULT1E1 | - | intron | silent | 0.019746121 | 0.009247028 |
| chr4 | 70709828 | A | G | SULT1E1 | - | intron | silent | 0.002820874 | 0 |
| chr4 | 70709895 | C | T | SULT1E1 | - | cds | synonymous | 0 | 0.001321004 |
| chr4 | 70709970 | C | T | SULT1E1 | - | cds | synonymous | 0 | 0.001321004 |
| chr4 | 70709999 | G | T | SULT1E1 | - | cds | missense | 0 | 0.001321004 |
| chr4 | 70710032 | T | C | SULT1E1 | - | cds | missense | 0.001410437 | 0.001321004 |
| chr4 | 70710085 | C | A | SULT1E1 | - | intron | silent | 0.001410437 | 0 |
| chr4 | 70713280 | T | A | SULT1E1 | - | intron | silent | 0 | 0.001322751 |
| chr4 | 70713284 | G | T | SULT1E1 | - | intron | silent | 0.002820874 | 0 |
| chr4 | 70713364 | A | G | SULT1E1 | - | intron | silent | 0 | 0.001321004 |
| chr4 | 70713422 | C | A | SULT1E1 | - | cds | synonymous | 0 | 0.001321004 |
| chr4 | 70713520 | G | C | SULT1E1 | - | intron | silent | 0 | 0.001321004 |
| chr4 | 70715066 | T | A | SULT1E1 | - | intron | silent | 0.001410437 | 0.001321004 |
| chr4 | 70715068 | C | T | SULT1E1 | - | intron | silent | 0.001410437 | 0.002642008 |
| chr4 | 70715116 | A | G | SULT1E1 | - | intron | silent | 0.002820874 | 0.011889036 |
| chr4 | 70715138 | A | G | SULT1E1 | - | intron | silent | 0.001410437 | 0 |
| chr4 | 70715206 | G | C | SULT1E1 | - | cds | missense | 0.004231312 | 0.001321004 |
| chr4 | 70715245 | C | T | SULT1E1 | - | cds | missense | 0 | 0.002642008 |
| chr4 | 70715333 | C | G | SULT1E1 | - | intron | silent | 0.001410437 | 0 |
| chr4 | 70715335 | C | T | SULT1E1 | - | intron | silent | 0.001410437 | 0 |
| chr4 | 70715380 | T | C | SULT1E1 | - | intron | silent | 0.001416431 | 0 |
| chr4 | 70715385 | C | T | SULT1E1 | - | intron | silent | 0 | 0.00132626 |
| chr4 | 70715403 | T | C | SULT1E1 | - | intron | silent | 0.005738881 | 0.00397878 |
| chr4 | 70719752 | G | T | SULT1E1 | - | intron | silent | 0.005730659 | 0 |
| chr4 | 70719753 | C | A | SULT1E1 | - | intron | silent | 0.001432665 | 0 |
| chr4 | 70720022 | T | G | SULT1E1 | - | cds | missense | 0 | 0.001321004 |
| chr4 | 70720097 | T | C | SULT1E1 | - | intron | silent | 0.002820874 | 0.002645503 |
| chr4 | 70720099 | G | A | SULT1E1 | - | intron | silent | 0 | 0.001322751 |
| chr4 | 70720112 | T | C | SULT1E1 | - | intron | silent | 0.057909605 | 0.062251656 |
| chr4 | 70720169 | A | C | SULT1E1 | - | intron | silent | 0.1056 | 0.102880658 |
| chr4 | 70720200 | T | C | SULT1E1 | - | intron | silent | 0.001941748 | 0 |
| chr4 | 70720845 | A | G | SULT1E1 | - | intron | silent | 0 | 0.001333333 |
| chr4 | 70720928 | C | A | SULT1E1 | - | intron | silent | 0.001410437 | 0 |
| chr4 | 70720930 | A | T | SULT1E1 | - | intron | silent | 0.005641749 | 0.001324503 |
| chr4 | 70720961 | T | C | SULT1E1 | - | intron | silent | 0 | 0.002649007 |
| chr4 | 70721061 | G | A | SULT1E1 | - | cds | nonsense | 0 | 0.001321004 |
| chr4 | 70721232 | A | G | SULT1E1 | - | intron | silent | 0 | 0.001321004 |
| chr4 | 70721314 | A | G | SULT1E1 | - | intron | silent | 0.001488095 | 0 |
| chr4 | 70723053 | A | C | SULT1E1 | - | intron | silent | 0.035816619 | 0.055702918 |
| chr4 | 70723068 | A | G | SULT1E1 | - | intron | silent | 0.001416431 | 0 |
| chr4 | 70723079 | C | A | SULT1E1 | - | intron | silent | 0.001412429 | 0.002642008 |
| chr4 | 70723106 | G | A | SULT1E1 | - | intron | silent | 0.001412429 | 0 |
| chr4 | 70723234 | G | A | SULT1E1 | - | cds | synonymous | 0.001410437 | 0 |
| chr4 | 70723291 | G | A | SULT1E1 | - | cds | synonymous | 0.001410437 | 0 |
| chr4 | 70723299 | C | A | SULT1E1 | - | cds | missense | 0 | 0.001321004 |
| chr4 | 70723331 | A | G | SULT1E1 | - | cds | missense | 0 | 0.001321004 |
| chr4 | 70723444 | C | G | SULT1E1 | - | intron | silent | 0.463276836 | 0.48015873 |
| chr4 | 70723511 | G | T | SULT1E1 | - | intron | silent | 0.002902758 | 0.009433962 |
| chr4 | 70723522 | A | T | SULT1E1 | - | intron | silent | 0.00295858 | 0 |
| chr19 | 10244217 | T | C | DNMT1 | - | utr3 | silent | 0.001410437 | 0 |
| chr19 | 10244232 | C | G | DNMT1 | - | utr3 | silent | 0.001410437 | 0.001321004 |
| chr19 | 10244297 | G | C | DNMT1 | - | utr3 | silent | 0.001410437 | 0 |
| chr19 | 10244309 | T | G | DNMT1 | - | utr3 | silent | 0.001410437 | 0 |
| chr19 | 10244325 | G | A | DNMT1 | - | utr3 | silent | 0 | 0.001321004 |
| chr19 | 10244357 | C | T | DNMT1 | - | cds | missense | 0 | 0.001321004 |
| chr19 | 10244421 | G | A | DNMT1 | - | intron | silent | 0.001410437 | 0 |
| chr19 | 10244442 | C | T | DNMT1 | - | intron | silent | 0 | 0.001321004 |
| chr19 | 10244554 | G | A | DNMT1 | - | intron | silent | 0.004559271 | 0.003169572 |
| chr19 | 10244700 | C | T | DNMT1 | - | intron | silent | 0 | 0.002141328 |
| chr19 | 10244772 | C | T | DNMT1 | - | intron | silent | 0 | 0.001422475 |
| chr19 | 10244823 | G | T | DNMT1 | - | intron | silent | 0.007092199 | 0.001338688 |
| chr19 | 10244834 | C | A | DNMT1 | - | intron | silent | 0.00141844 | 0 |
| chr19 | 10244887 | C | T | DNMT1 | - | intron | silent | 0 | 0.001321004 |
| chr19 | 10245000 | G | A | DNMT1 | - | intron | silent | 0.001420455 | 0.002673797 |
| chr19 | 10246221 | T | C | DNMT1 | - | intron | silent | 0 | 0.002118644 |
| chr19 | 10246240 | G | A | DNMT1 | - | intron | silent | 0.001607717 | 0 |
| chr19 | 10246401 | G | A | DNMT1 | - | intron | silent | 0 | 0.001322751 |
| chr19 | 10246584 | C | T | DNMT1 | - | intron | silent | 0 | 0.001328021 |
| chr19 | 10246620 | C | T | DNMT1 | - | intron | silent | 0 | 0.001336898 |
| chr19 | 10246651 | G | A | DNMT1 | - | intron | silent | 0 | 0.001356852 |
| chr19 | 10246657 | G | A | DNMT1 | - | intron | silent | 0.00141844 | 0 |
| chr19 | 10246747 | G | A | DNMT1 | - | intron | silent | 0 | 0.001331558 |
| chr19 | 10246784 | C | A | DNMT1 | - | intron | silent | 0.004237288 | 0.003968254 |
| chr19 | 10246815 | G | A | DNMT1 | - | cds | synonymous | 0.001412429 | 0 |
| chr19 | 10246944 | G | A | DNMT1 | - | cds | synonymous | 0.001410437 | 0 |
| chr19 | 10246959 | G | C | DNMT1 | - | cds | synonymous | 0 | 0.001322751 |
| chr19 | 10246983 | T | C | DNMT1 | - | intron | silent | 0 | 0.001324503 |
| chr19 | 10247041 | G | C | DNMT1 | - | intron | silent | 0.001477105 | 0 |
| chr19 | 10247767 | C | T | DNMT1 | - | cds | missense | 0 | 0.001321004 |
| chr19 | 10247797 | A | G | DNMT1 | - | cds | missense | 0 | 0.001321004 |
| chr19 | 10247948 | A | G | DNMT1 | - | cds | synonymous | 0 | 0.001322751 |
| chr19 | 10247973 | G | T | DNMT1 | - | intron | silent | 0.041134752 | 0.050331126 |
| chr19 | 10247975 | C | G | DNMT1 | - | intron | silent | 0.041134752 | 0.050331126 |
| chr19 | 10248347 | C | A | DNMT1 | - | intron | silent | 0.004405286 | 0.0013947 |
| chr19 | 10248379 | C | T | DNMT1 | - | intron | silent | 0.012729844 | 0.01193634 |
| chr19 | 10248412 | T | C | DNMT1 | - | intron | silent | 0.001412429 | 0 |
| chr19 | 10248482 | G | A | DNMT1 | - | intron | silent | 0.001410437 | 0 |
| chr19 | 10248547 | G | A | DNMT1 | - | cds | synonymous | 0 | 0.001321004 |
| chr19 | 10248784 | G | A | DNMT1 | - | intron | silent | 0 | 0.002713704 |
| chr19 | 10248797 | T | C | DNMT1 | - | intron | silent | 0.004451039 | 0.002766252 |
| chr19 | 10248917 | A | G | DNMT1 | - | intron | silent | 0.001567398 | 0 |
| chr19 | 10248992 | G | A | DNMT1 | - | intron | silent | 0.004379562 | 0 |
| chr19 | 10249067 | A | C | DNMT1 | - | intron | silent | 0.001412429 | 0 |
| chr19 | 10249153 | C | G | DNMT1 | - | cds | synonymous | 0.008462623 | 0.009247028 |
| chr19 | 10249228 | C | T | DNMT1 | - | cds | synonymous | 0 | 0.001321004 |
| chr19 | 10249270 | G | A | DNMT1 | - | cds | synonymous | 0.001410437 | 0 |
| chr19 | 10250211 | G | A | DNMT1 | - | intron | silent | 0 | 0.001663894 |
| chr19 | 10250502 | G | A | DNMT1 | - | intron | silent | 0 | 0.001321004 |
| chr19 | 10250567 | C | T | DNMT1 | - | intron | silent | 0.001410437 | 0 |
| chr19 | 10250631 | G | A | DNMT1 | - | intron | silent | 0 | 0.001321004 |
| chr19 | 10250669 | T | C | DNMT1 | - | intron | silent | 0.001410437 | 0 |
| chr19 | 10250672 | G | A | DNMT1 | - | intron | silent | 0.001410437 | 0 |
| chr19 | 10250677 | A | G | DNMT1 | - | intron | silent | 0 | 0.001322751 |
| chr19 | 10250687 | C | T | DNMT1 | - | intron | silent | 0.001410437 | 0 |
| chr19 | 10250697 | C | A | DNMT1 | - | intron | silent | 0.004231312 | 0.003963012 |
| chr19 | 10250814 | G | A | DNMT1 | - | cds | synonymous | 0.001414427 | 0 |
| chr19 | 10250817 | G | A | DNMT1 | - | cds | synonymous | 0.002828854 | 0 |
| chr19 | 10251076 | C | T | DNMT1 | - | intron | silent | 0 | 0.00245098 |
| chr19 | 10251324 | G | A | DNMT1 | - | intron | silent | 0.001540832 | 0 |
| chr19 | 10251537 | A | C | DNMT1 | - | cds | missense | 0.001410437 | 0.001321004 |
| chr19 | 10251572 | G | C | DNMT1 | - | cds | synonymous | 0.019746121 | 0.017173052 |
| chr19 | 10251649 | C | T | DNMT1 | - | intron | silent | 0.001410437 | 0.001321004 |
| chr19 | 10251700 | G | A | DNMT1 | - | intron | silent | 0 | 0.001321004 |
| chr19 | 10251719 | T | C | DNMT1 | - | intron | silent | 0.001410437 | 0 |
| chr19 | 10251747 | A | G | DNMT1 | - | intron | silent | 0.475317348 | 0.48348745 |
| chr19 | 10251824 | G | A | DNMT1 | - | cds | synonymous | 0.002820874 | 0.010568032 |
| chr19 | 10251903 | C | T | DNMT1 | - | intron | silent | 0.002820874 | 0.001321004 |
| chr19 | 10252047 | T | C | DNMT1 | - | intron | silent | 0.034375 | 0.033285094 |
| chr19 | 10252546 | C | T | DNMT1 | - | intron | silent | 0.001443001 | 0 |
| chr19 | 10252623 | G | A | DNMT1 | - | intron | silent | 0.001410437 | 0 |
| chr19 | 10252752 | G | A | DNMT1 | - | cds | synonymous | 0.001410437 | 0.002642008 |
| chr19 | 10252797 | C | A | DNMT1 | - | cds | synonymous | 0.001410437 | 0 |
| chr19 | 10252827 | G | A | DNMT1 | - | cds | synonymous | 0.002820874 | 0.005284016 |
| chr19 | 10252866 | C | T | DNMT1 | - | cds | synonymous | 0 | 0.003963012 |
| chr19 | 10252913 | C | G | DNMT1 | - | intron | silent | 0.001410437 | 0 |
| chr19 | 10253019 | C | T | DNMT1 | - | intron | silent | 0 | 0.001340483 |
| chr19 | 10254338 | C | T | DNMT1 | - | intron | silent | 0.004231312 | 0.003963012 |
| chr19 | 10254379 | G | T | DNMT1 | - | intron | silent | 0 | 0.001321004 |
| chr19 | 10254416 | G | A | DNMT1 | - | intron | silent | 0.001410437 | 0 |
| chr19 | 10254460 | C | T | DNMT1 | - | cds | missense | 0.005641749 | 0.001321004 |
| chr19 | 10254761 | A | C | DNMT1 | - | intron | silent | 0.04090268 | 0.05026455 |
| chr19 | 10256828 | C | T | DNMT1 | - | intron | silent | 0.043841336 | 0.063136456 |
| chr19 | 10256908 | G | A | DNMT1 | - | intron | silent | 0.001420455 | 0.001345895 |
| chr19 | 10256927 | G | T | DNMT1 | - | intron | silent | 0.001438849 | 0 |
| chr19 | 10256934 | G | A | DNMT1 | - | intron | silent | 0.004267425 | 0.001369863 |
| chr19 | 10256965 | A | G | DNMT1 | - | intron | silent | 0 | 0.001331558 |
| chr19 | 10256983 | A | G | DNMT1 | - | intron | silent | 0.001410437 | 0 |
| chr19 | 10256997 | T | G | DNMT1 | - | intron | silent | 0.001410437 | 0 |
| chr19 | 10257066 | C | G | DNMT1 | - | cds | missense | 0 | 0.001321004 |
| chr19 | 10257245 | G | A | DNMT1 | - | intron | silent | 0.473833098 | 0.450199203 |
| chr19 | 10257298 | G | A | DNMT1 | - | intron | silent | 0 | 0.001321004 |
| chr19 | 10257359 | A | G | DNMT1 | - | intron | silent | 0.00147929 | 0 |
| chr19 | 10259376 | A | G | DNMT1 | - | intron | silent | 0 | 0.001373626 |
| chr19 | 10259463 | G | A | DNMT1 | - | intron | silent | 0.001412429 | 0 |
| chr19 | 10259466 | G | A | DNMT1 | - | intron | silent | 0 | 0.001321004 |
| chr19 | 10259467 | T | C | DNMT1 | - | intron | silent | 0.001410437 | 0 |
| chr19 | 10259535 | C | A | DNMT1 | - | intron | silent | 0.001410437 | 0 |
| chr19 | 10259654 | C | T | DNMT1 | - | cds | missense | 0.001410437 | 0.001321004 |
| chr19 | 10259674 | G | A | DNMT1 | - | cds | missense | 0 | 0.001321004 |
| chr19 | 10259711 | C | A | DNMT1 | - | intron | silent | 0 | 0.001321004 |
| chr19 | 10259756 | A | G | DNMT1 | - | intron | silent | 0.001416431 | 0.001328021 |
| chr19 | 10259773 | G | A | DNMT1 | - | intron | silent | 0.001438849 | 0 |
| chr19 | 10259988 | C | T | DNMT1 | - | intron | silent | 0.002304147 | 0.001453488 |
| chr19 | 10260030 | C | A | DNMT1 | - | intron | silent | 0.307254623 | 0.338177015 |
| chr19 | 10260067 | C | T | DNMT1 | - | intron | silent | 0.001412429 | 0 |
| chr19 | 10260228 | C | T | DNMT1 | - | cds | synonymous | 0 | 0.001321004 |
| chr19 | 10260240 | C | T | DNMT1 | - | cds | synonymous | 0.001410437 | 0 |
| chr19 | 10260252 | G | A | DNMT1 | - | cds | synonymous | 0.007052186 | 0.007926024 |
| chr19 | 10260337 | G | A | DNMT1 | - | intron | silent | 0.039492243 | 0.050198151 |
| chr19 | 10260455 | A | G | DNMT1 | - | intron | silent | 0.039492243 | 0.050198151 |
| chr19 | 10260480 | C | T | DNMT1 | - | intron | silent | 0.001410437 | 0 |
| chr19 | 10260669 | G | A | DNMT1 | - | intron | silent | 0 | 0.001321004 |
| chr19 | 10260742 | G | A | DNMT1 | - | intron | silent | 0 | 0.001321004 |
| chr19 | 10260786 | T | C | DNMT1 | - | intron | silent | 0 | 0.001322751 |
| chr19 | 10261914 | C | T | DNMT1 | - | intron | silent | 0 | 0.001349528 |
| chr19 | 10261968 | G | A | DNMT1 | - | intron | silent | 0.039492243 | 0.050198151 |
| chr19 | 10262188 | G | A | DNMT1 | - | cds | synonymous | 0.001410437 | 0 |
| chr19 | 10262240 | T | C | DNMT1 | - | intron | silent | 0 | 0.001321004 |
| chr19 | 10262272 | C | T | DNMT1 | - | intron | silent | 0.016925247 | 0.018494055 |
| chr19 | 10262651 | C | T | DNMT1 | - | intron | silent | 0.346036585 | 0.343532684 |
| chr19 | 10264825 | C | T | DNMT1 | - | intron | silent | 0.001577287 | 0 |
| chr19 | 10264945 | G | A | DNMT1 | - | intron | silent | 0 | 0.001321004 |
| chr19 | 10265077 | G | C | DNMT1 | - | cds | synonymous | 0.001410437 | 0 |
| chr19 | 10265092 | C | T | DNMT1 | - | cds | synonymous | 0.001410437 | 0 |
| chr19 | 10265141 | C | G | DNMT1 | - | cds | missense | 0 | 0.001321004 |
| chr19 | 10265248 | T | C | DNMT1 | - | intron | silent | 0.069111425 | 0.084544254 |
| chr19 | 10265293 | T | C | DNMT1 | - | cds | missense | 0 | 0.001321004 |
| chr19 | 10265372 | C | T | DNMT1 | - | cds | synonymous | 0.001410437 | 0.003963012 |
| chr19 | 10265444 | C | T | DNMT1 | - | cds | synonymous | 0 | 0.001321004 |
| chr19 | 10265543 | G | A | DNMT1 | - | intron | silent | 0 | 0.002642008 |
| chr19 | 10265593 | G | T | DNMT1 | - | cds | synonymous | 0.004231312 | 0.002642008 |
| chr19 | 10265689 | C | T | DNMT1 | - | cds | synonymous | 0 | 0.001321004 |
| chr19 | 10265725 | G | A | DNMT1 | - | cds | synonymous | 0.039492243 | 0.050198151 |
| chr19 | 10265814 | C | T | DNMT1 | - | intron | silent | 0.001410437 | 0 |
| chr19 | 10265839 | C | T | DNMT1 | - | intron | silent | 0 | 0.00132626 |
| chr19 | 10266456 | G | A | DNMT1 | - | intron | silent | 0 | 0.00660502 |
| chr19 | 10266542 | G | A | DNMT1 | - | cds | synonymous | 0.001410437 | 0 |
| chr19 | 10266689 | C | T | DNMT1 | - | intron | silent | 0 | 0.001321004 |
| chr19 | 10266772 | A | G | DNMT1 | - | intron | silent | 0.001414427 | 0 |
| chr19 | 10266935 | C | T | DNMT1 | - | intron | silent | 0 | 0.001321004 |
| chr19 | 10266938 | G | A | DNMT1 | - | intron | silent | 0.007052186 | 0.005291005 |
| chr19 | 10267011 | C | A | DNMT1 | - | intron | silent | 0.308885755 | 0.339498018 |
| chr19 | 10267077 | T | C | DNMT1 | - | cds | synonymous | 0.070521862 | 0.084544254 |
| chr19 | 10267137 | G | A | DNMT1 | - | cds | synonymous | 0.001410437 | 0 |
| chr19 | 10267210 | T | A | DNMT1 | - | intron | silent | 0.001410437 | 0 |
| chr19 | 10267252 | G | T | DNMT1 | - | intron | silent | 0.307475317 | 0.339498018 |
| chr19 | 10267266 | C | A | DNMT1 | - | intron | silent | 0.307475317 | 0.339498018 |
| chr19 | 10267327 | T | G | DNMT1 | - | intron | silent | 0.002994012 | 0 |
| chr19 | 10270256 | C | G | DNMT1 | - | intron | silent | 0.001410437 | 0 |
| chr19 | 10270319 | C | G | DNMT1 | - | intron | silent | 0.001410437 | 0 |
| chr19 | 10270431 | G | C | DNMT1 | - | cds | missense | 0.001410437 | 0 |
| chr19 | 10270572 | G | A | DNMT1 | - | cds | synonymous | 0.001410437 | 0.001321004 |
| chr19 | 10270622 | A | G | DNMT1 | - | intron | silent | 0 | 0.001321004 |
| chr19 | 10270623 | T | C | DNMT1 | - | intron | silent | 0 | 0.001321004 |
| chr19 | 10270648 | C | T | DNMT1 | - | intron | silent | 0.007052186 | 0.005284016 |
| chr19 | 10270681 | G | C | DNMT1 | - | intron | silent | 0 | 0.001321004 |
| chr19 | 10270801 | G | T | DNMT1 | - | intron | silent | 0.001412429 | 0 |
| chr19 | 10271001 | T | C | DNMT1 | - | intron | silent | 0 | 0.001321004 |
| chr19 | 10271027 | C | T | DNMT1 | - | intron | silent | 0 | 0.001321004 |
| chr19 | 10271034 | C | G | DNMT1 | - | intron | silent | 0.275035261 | 0.295904888 |
| chr19 | 10271141 | A | C | DNMT1 | - | intron | silent | 0.300423131 | 0.324966975 |
| chr19 | 10271286 | T | C | DNMT1 | - | intron | silent | 0.002178649 | 0 |
| chr19 | 10273226 | G | A | DNMT1 | - | intron | silent | 0 | 0.001381215 |
| chr19 | 10273270 | G | C | DNMT1 | - | intron | silent | 0 | 0.001329787 |
| chr19 | 10273280 | G | A | DNMT1 | - | intron | silent | 0.012729844 | 0.006648936 |
| chr19 | 10273355 | G | A | DNMT1 | - | cds | synonymous | 0.1325811 | 0.138705416 |
| chr19 | 10273372 | T | C | DNMT1 | - | cds | missense | 0.307475317 | 0.338177015 |
| chr19 | 10273450 | A | C | DNMT1 | - | intron | silent | 0.001410437 | 0 |
| chr19 | 10273620 | C | T | DNMT1 | - | intron | silent | 0.041218638 | 0.052845528 |
| chr19 | 10273862 | C | T | DNMT1 | - | intron | silent | 0 | 0.001322751 |
| chr19 | 10273863 | G | A | DNMT1 | - | intron | silent | 0.001412429 | 0 |
| chr19 | 10274054 | G | A | DNMT1 | - | intron | silent | 0.001410437 | 0 |
| chr19 | 10274188 | G | A | DNMT1 | - | intron | silent | 0.001416431 | 0 |
| chr19 | 10274200 | G | C | DNMT1 | - | intron | silent | 0.00286123 | 0 |
| chr19 | 10277178 | G | A | DNMT1 | - | intron | silent | 0.004237288 | 0.002670227 |
| chr19 | 10277213 | G | A | DNMT1 | - | intron | silent | 0 | 0.001321004 |
| chr19 | 10277382 | C | A | DNMT1 | - | intron | silent | 0 | 0.001321004 |
| chr19 | 10277415 | A | C | DNMT1 | - | intron | silent | 0 | 0.001342282 |
| chr19 | 10277504 | C | T | DNMT1 | - | intron | silent | 0.002886003 | 0 |
| chr19 | 10277509 | G | A | DNMT1 | - | intron | silent | 0.00143472 | 0.00136612 |
| chr19 | 10277542 | G | A | DNMT1 | - | intron | silent | 0.00297619 | 0.001459854 |
| chr19 | 10278909 | T | A | DNMT1 | - | intron | silent | 0.001410437 | 0 |
| chr19 | 10278951 | T | C | DNMT1 | - | intron | silent | 0.005641749 | 0.005284016 |
| chr19 | 10279141 | G | A | DNMT1 | - | intron | silent | 0.008462623 | 0.00660502 |
| chr19 | 10279189 | T | C | DNMT1 | - | intron | silent | 0.070721358 | 0.084880637 |
| chr19 | 10283604 | A | G | DNMT1 | - | intron | silent | 0.001414427 | 0 |
| chr19 | 10283625 | A | G | DNMT1 | - | intron | silent | 0.001412429 | 0 |
| chr19 | 10283634 | G | A | DNMT1 | - | intron | silent | 0.001410437 | 0 |
| chr19 | 10283709 | T | A | DNMT1 | - | intron | silent | 0 | 0.001321004 |
| chr19 | 10283723 | G | A | DNMT1 | - | intron | silent | 0.001410437 | 0 |
| chr19 | 10283745 | T | A | DNMT1 | - | intron | silent | 0.001410437 | 0 |
| chr19 | 10283774 | C | G | DNMT1 | - | cds | missense | 0 | 0.001321004 |
| chr19 | 10283932 | C | T | DNMT1 | - | intron | silent | 0.307475317 | 0.338177015 |
| chr19 | 10284023 | G | A | DNMT1 | - | intron | silent | 0.005747126 | 0.010914052 |
| chr19 | 10284415 | A | G | DNMT1 | - | intron | silent | 0.284908322 | 0.306472919 |
| chr19 | 10284461 | C | T | DNMT1 | - | intron | silent | 0.004231312 | 0.002642008 |
| chr19 | 10284722 | G | C | DNMT1 | - | intron | silent | 0.002824859 | 0 |
| chr19 | 10284778 | A | G | DNMT1 | - | intron | silent | 0.074738416 | 0.088455772 |
| chr19 | 10286067 | T | G | DNMT1 | - | intron | silent | 0 | 0.001321004 |
| chr19 | 10286087 | T | C | DNMT1 | - | intron | silent | 0 | 0.002642008 |
| chr19 | 10286107 | T | A | DNMT1 | - | intron | silent | 0.001410437 | 0 |
| chr19 | 10286117 | G | A | DNMT1 | - | intron | silent | 0 | 0.001321004 |
| chr19 | 10286328 | A | T | DNMT1 | - | intron | silent | 0.002820874 | 0 |
| chr19 | 10286360 | G | A | DNMT1 | - | intron | silent | 0.001410437 | 0 |
| chr19 | 10286373 | C | A | DNMT1 | - | intron | silent | 0.001410437 | 0 |
| chr19 | 10286415 | T | C | DNMT1 | - | intron | silent | 0 | 0.001321004 |
| chr19 | 10287789 | A | G | DNMT1 | - | intron | silent | 0 | 0.001338688 |
| chr19 | 10287810 | G | A | DNMT1 | - | intron | silent | 0.001410437 | 0.001324503 |
| chr19 | 10287844 | A | G | DNMT1 | - | intron | silent | 0.001410437 | 0 |
| chr19 | 10287894 | G | A | DNMT1 | - | intron | silent | 0.005641749 | 0.010568032 |
| chr19 | 10287905 | C | A | DNMT1 | - | intron | silent | 0.001410437 | 0.003963012 |
| chr19 | 10291113 | C | G | DNMT1 | - | cds | missense | 0.1325811 | 0.137384412 |
| chr19 | 10291118 | C | G | DNMT1 | - | cds | missense | 0 | 0.001321004 |
| chr19 | 10291170 | G | A | DNMT1 | - | cds | missense | 0 | 0.001321004 |
| chr19 | 10291181 | T | C | DNMT1 | - | cds | missense | 0.36248237 | 0.35667107 |
| chr19 | 10291353 | G | A | DNMT1 | - | intron | silent | 0.001410437 | 0 |
| chr19 | 10291361 | G | A | DNMT1 | - | intron | silent | 0.001410437 | 0 |
| chr19 | 10291612 | T | C | DNMT1 | - | intron | silent | 0 | 0.002642008 |
| chr19 | 10291627 | C | G | DNMT1 | - | intron | silent | 0.001410437 | 0 |
| chr19 | 10291657 | A | G | DNMT1 | - | intron | silent | 0 | 0.001322751 |
| chr19 | 10291755 | A | C | DNMT1 | - | intron | silent | 0 | 0.001494768 |
| chr19 | 10292566 | G | A | DNMT1 | - | intron | silent | 0 | 0.001324503 |
| chr19 | 10292698 | A | G | DNMT1 | - | intron | silent | 0.039492243 | 0.050198151 |
| chr19 | 10292759 | A | G | DNMT1 | - | intron | silent | 0 | 0.005284016 |
| chr19 | 10292793 | T | C | DNMT1 | - | intron | silent | 0.001410437 | 0 |
| chr19 | 10292850 | A | G | DNMT1 | - | intron | silent | 0.012693935 | 0.009259259 |
| chr19 | 10292883 | A | T | DNMT1 | - | intron | silent | 0.004237288 | 0.001333333 |
| chr19 | 10292901 | C | T | DNMT1 | - | intron | silent | 0.125894134 | 0.105548038 |
| chr19 | 10292902 | G | A | DNMT1 | - | intron | silent | 0.00286533 | 0 |
| chr19 | 10305366 | C | G | DNMT1 | - | intron | silent | 0.002298851 | 0 |
| chr19 | 10305409 | G | A | DNMT1 | - | intron | silent | 0.030947776 | 0.056962025 |
| chr19 | 10305623 | T | G | DNMT1 | - | utr5 | silent | 0.001636661 | 0 |
| chr19 | 10305626 | T | A | DNMT1 | - | utr5 | silent | 0.00166113 | 0 |
| chr19 | 10305634 | A | G | DNMT1 | - | utr5 | silent | 0 | 0.005076142 |

**Reference**

1. Matzuk, M.M. & Lamb, D.J. The biology of infertility: research advances and clinical challenges. *Nat Med* **14**, 1197-213 (2008).

2. Drozdzik, M. *et al.* Association of the MDR1 (ABCB1) gene 3435C>T polymorphism with male infertility. *Pharmacol Rep* **61**, 690-6 (2009).

3. Belangero, S.I. *et al.* Male infertility related to an aberrant karyotype, 46,XY,9ph,9qh+. *Fertil Steril* **91**, 2732 e1-3 (2009).

4. Gong, E.Y., Park, E., Lee, H.J. & Lee, K. Expression of Atp8b3 in murine testis and its characterization as a testis specific P-type ATPase. *Reproduction* **137**, 345-51 (2009).

5. Xiao, P., Tang, A., Yu, Z., Gui, Y. & Cai, Z. Gene expression profile of 2058 spermatogenesis-related genes in mice. *Biol Pharm Bull* **31**, 201-6 (2008).

6. Tang, A. *et al.* Identification and characteristics of a novel testis-specific gene, Tsc24, in human and mice. *Biol Pharm Bull* **29**, 2187-91 (2006).

7. Geyer, C.B. *et al.* A missense mutation in the Capza3 gene and disruption of F-actin organization in spermatids of repro32 infertile male mice. *Dev Biol* **330**, 142-52 (2009).

8. Zhang, Y. *et al.* Sensorineural deafness and male infertility: a contiguous gene deletion syndrome. *BMJ Case Rep* **2009**(2009).

9. Jamsai, D. *et al.* A novel protein, sperm head and tail associated protein (SHTAP), interacts with cysteine-rich secretory protein 2 (CRISP2) during spermatogenesis in the mouse. *Biol Cell* **102**, 93-106 (2010).

10. Aydos, S.E., Taspinar, M., Sunguroglu, A. & Aydos, K. Association of CYP1A1 and glutathione S-transferase polymorphisms with male factor infertility. *Fertil Steril* **92**, 541-7 (2009).

11. Skaletsky, H. *et al.* The male-specific region of the human Y chromosome is a mosaic of discrete sequence classes. *Nature* **423**, 825-37 (2003).

12. Zhou, J. *et al.* RIM-BP3 is a manchette-associated protein essential for spermiogenesis. *Development* **136**, 373-82 (2009).

13. von Kopylow, K. *et al.* Screening for biomarkers of spermatogonia within the human testis: a whole genome approach. *Hum Reprod* **25**, 1104-12 (2010).

14. Rainey, M.A. *et al.* The endocytic recycling regulator EHD1 is essential for spermatogenesis and male fertility in mice. *BMC Dev Biol* **10**, 37 (2010).

15. Kleiman, S.E. *et al.* Expression profile of AZF genes in testicular biopsies of azoospermic men. *Hum Reprod* **22**, 151-8 (2007).

16. Sun, F., Palmer, K. & Handel, M.A. Mutation of Eif4g3, encoding a eukaryotic translation initiation factor, causes male infertility and meiotic arrest of mouse spermatocytes. *Development* **137**, 1699-707 (2010).

17. Bonaparte, E. *et al.* ESX1 gene expression as a robust marker of residual spermatogenesis in azoospermic men. *Hum Reprod* **25**, 1398-403 (2010).

18. Wang, W. *et al.* FAS and FASLG polymorphisms and susceptibility to idiopathic azoospermia or severe oligozoospermia. *Reprod Biomed Online* **18**, 141-7 (2009).

19. Juul, A. *et al.* Preserved fertility in a non-mosaic Klinefelter patient with a mutation in the fibroblast growth factor receptor 3 gene: case report. *Hum Reprod* **22**, 1907-11 (2007).

20. Sunnotel, O. *et al.* Alterations in the steroid hormone receptor co-chaperone FKBPL are associated with male infertility: a case-control study. *Reprod Biol Endocrinol* **8**, 22 (2010).

21. Vidarsson, H. *et al.* The forkhead transcription factor Foxi1 is a master regulator of vacuolar H-ATPase proton pump subunits in the inner ear, kidney and epididymis. *PLoS One* **4**, e4471 (2009).

22. Coffee, R.L., Jr., Tessier, C.R., Woodruff, E.A., 3rd & Broadie, K. Fragile X mental retardation protein has a unique, evolutionarily conserved neuronal function not shared with FXR1P or FXR2P. *Dis Model Mech* **3**, 471-85 (2010).

23. Hu, Y. *et al.* RGS22, a novel testis-specific regulator of G-protein signaling involved in human and mouse spermiogenesis along with GNA12/13 subunits. *Biol Reprod* **79**, 1021-9 (2008).

24. Ng, M.C. *et al.* Implication of genetic variants near NEGR1, SEC16B, TMEM18, ETV5/DGKG, GNPDA2, LIN7C/BDNF, MTCH2, BCDIN3D/FAIM2, SH2B1, FTO, MC4R, and KCTD15 with obesity and type 2 diabetes in 7705 Chinese. *J Clin Endocrinol Metab* **95**, 2418-25 (2010).

25. Boitani, C. & Puglisi, R. Selenium, a key element in spermatogenesis and male fertility. *Adv Exp Med Biol* **636**, 65-73 (2008).

26. Lee, J. *et al.* Functional polymorphism in H2BFWT-5'UTR is associated with susceptibility to male infertility. *J Cell Mol Med* **13**, 1942-51 (2009).

27. Buretic-Tomljanovic, A. *et al.* The impact of hemochromatosis mutations and transferrin genotype on gonadotropin serum levels in infertile men. *Fertil Steril* **91**, 1793-800 (2009).

28. Strauss, L. *et al.* Increased exposure to estrogens disturbs maturation, steroidogenesis, and cholesterol homeostasis via estrogen receptor alpha in adult mouse Leydig cells. *Endocrinology* **150**, 2865-72 (2009).

29. Xiao, N. *et al.* PICK1 deficiency causes male infertility in mice by disrupting acrosome formation. *J Clin Invest* **119**, 802-12 (2009).

30. Santi, C.M. *et al.* The SLO3 sperm-specific potassium channel plays a vital role in male fertility. *FEBS Lett* **584**, 1041-6 (2010).

31. Okada, Y., Tateishi, K. & Zhang, Y. Histone demethylase JHDM2A is involved in male infertility and obesity. *J Androl* **31**, 75-8 (2010).

32. Sette, C., Messina, V. & Paronetto, M.P. Sam68: a new STAR in the male fertility firmament. *J Androl* **31**, 66-74 (2010).

33. Greenbaum, M.P., Iwamori, N., Agno, J.E. & Matzuk, M.M. Mouse TEX14 is required for embryonic germ cell intercellular bridges but not female fertility. *Biol Reprod* **80**, 449-57 (2009).

34. Valbuena, G., Madrid, J.F., Hernandez, F. & Saez, F.J. Identification of fucosylated glycoconjugates in Xenopus laevis testis by lectin histochemistry. *Histochem Cell Biol* **134**, 215-25 (2010).

35. Lou, Z. *et al.* MDC1 maintains genomic stability by participating in the amplification of ATM-dependent DNA damage signals. *Mol Cell* **21**, 187-200 (2006).

36. Frost, R.J. *et al.* MOV10L1 is necessary for protection of spermatocytes against retrotransposons by Piwi-interacting RNAs. *Proc Natl Acad Sci U S A* **107**, 11847-52 (2010).

37. Huen, M.S. *et al.* Regulation of chromatin architecture by the PWWP domain-containing DNA damage-responsive factor EXPAND1/MUM1. *Mol Cell* **37**, 854-64 (2010).

38. Nakamura, B.N. *et al.* Knockout of the transcription factor NRF2 disrupts spermatogenesis in an age-dependent manner. *Free Radic Biol Med* **49**, 1368-79 (2010).

39. Volle, D.H. *et al.* The orphan nuclear receptor small heterodimer partner mediates male infertility induced by diethylstilbestrol in mice. *J Clin Invest* **119**, 3752-64 (2009).

40. Wagner, S.A. *et al.* A proteome-wide, quantitative survey of in vivo ubiquitylation sites reveals widespread regulatory roles. *Mol Cell Proteomics* **10**, M111 013284 (2011).

41. Million Passe, C.M. *et al.* Loss of the protein NUPR1 (p8) leads to delayed LHB expression, delayed ovarian maturation, and testicular development of a sertoli-cell-only syndrome-like phenotype in mice. *Biol Reprod* **79**, 598-607 (2008).

42. Aston, K.I. & Carrell, D.T. Genome-wide study of single-nucleotide polymorphisms associated with azoospermia and severe oligozoospermia. *J Androl* **30**, 711-25 (2009).

43. Wang, H. *et al.* A spermatogenesis-related gene expression profile in human spermatozoa and its potential clinical applications. *J Mol Med (Berl)* **82**, 317-24 (2004).

44. Plaseski, T., Noveski, P., Popeska, Z., Efremov, G.D. & Plaseska-Karanfilska, D. Association study of single-nucleotide polymorphisms in FASLG, JMJDIA, LOC203413, TEX15, BRDT, OR2W3, INSR, and TAS2R38 genes with male infertility. *J Androl* **33**, 675-83 (2012).

45. Yanagiya, A., Delbes, G., Svitkin, Y.V., Robaire, B. & Sonenberg, N. The poly(A)-binding protein partner Paip2a controls translation during late spermiogenesis in mice. *J Clin Invest* **120**, 3389-400 (2010).

46. Le, Y.J., Kim, H., Chung, J.H. & Lee, Y. Testis-specific expression of an intronless gene encoding a human poly(A) polymerase. *Mol Cells* **11**, 379-85 (2001).

47. Pomara, G. *et al.* Alterations in sperm motility after acute oral administration of sildenafil or tadalafil in young, infertile men. *Fertil Steril* **88**, 860-5 (2007).

48. Pinkas, H. *et al.* Platelet-derived growth factors (PDGF-A and -B) and their receptors in human fetal and adult ovaries. *Mol Hum Reprod* **14**, 199-206 (2008).

49. Song, H. *et al.* Cytosolic phospholipase A2alpha is crucial [correction of A2alpha deficiency is crucial] for 'on-time' embryo implantation that directs subsequent development. *Development* **129**, 2879-89 (2002).

50. Rajender, S., Avery, K. & Agarwal, A. Epigenetics, spermatogenesis and male infertility. *Mutat Res* **727**, 62-71 (2011).

51. Kashir, J. *et al.* Oocyte activation, phospholipase C zeta and human infertility. *Hum Reprod Update* **16**, 690-703 (2010).

52. Pearse, R.V., 2nd *et al.* Reduced fertility in mice deficient for the POU protein sperm-1. *Proc Natl Acad Sci U S A* **94**, 7555-60 (1997).

53. Zhang, Y. *et al.* Sensorineural deafness and male infertility: a contiguous gene deletion syndrome. *J Med Genet* **44**, 233-40 (2007).

54. Miyamoto, T. *et al.* Two single nucleotide polymorphisms in PRDM9 (MEISETZ) gene may be a genetic risk factor for Japanese patients with azoospermia by meiotic arrest. *J Assist Reprod Genet* **25**, 553-7 (2008).

55. Imken, L. *et al.* Mutations in the protamine locus: association with spermatogenic failure? *Mol Hum Reprod* **15**, 733-8 (2009).

56. Sinisi, A.A. *et al.* Homozygous mutation in the prokineticin-receptor2 gene (Val274Asp) presenting as reversible Kallmann syndrome and persistent oligozoospermia: case report. *Hum Reprod* **23**, 2380-4 (2008).

57. Kawano, N. *et al.* Mice lacking two sperm serine proteases, ACR and PRSS21, are subfertile, but the mutant sperm are infertile in vitro. *Biol Reprod* **83**, 359-69 (2010).

58. Ivanova, M. *et al.* Scaffold attachment factor B1 functions in development, growth, and reproduction. *Mol Cell Biol* **25**, 2995-3006 (2005).

59. Townson, S.M. *et al.* SAFB2, a new scaffold attachment factor homolog and estrogen receptor corepressor. *J Biol Chem* **278**, 20059-68 (2003).

60. Lin, Y.H. *et al.* The expression level of septin12 is critical for spermiogenesis. *Am J Pathol* **174**, 1857-68 (2009).

61. Altmae, S. *et al.* Variations in folate pathway genes are associated with unexplained female infertility. *Fertil Steril* **94**, 130-7 (2010).

62. Wagenfeld, A., Yeung, C.H., Lehnert, W., Nieschlag, E. & Cooper, T.G. Lack of glutamate transporter EAAC1 in the epididymis of infertile c-ros receptor tyrosine-kinase deficient mice. *J Androl* **23**, 772-82 (2002).

63. Dasoula, A., Georgiou, I., Kontogianni, E., Sofikitis, N. & Syrrou, M. Methylation status of the SNRPN and HUMARA genes in testicular biopsy samples. *Fertil Steril* **87**, 805-9 (2007).

64. Drabovich, A.P., Jarvi, K. & Diamandis, E.P. Verification of male infertility biomarkers in seminal plasma by multiplex selected reaction monitoring assay. *Mol Cell Proteomics* **10**, M110 004127 (2011).

65. Miyamoto, T. *et al.* A single nucleotide polymorphism in SPATA17 may be a genetic risk factor for Japanese patients with meiotic arrest. *Asian J Androl* **11**, 623-8 (2009).

66. La Salle, S. *et al.* Spata22, a novel vertebrate-specific gene, is required for meiotic progress in mouse germ cells. *Biol Reprod* **86**, 45 (2012).

67. Dube, E., Hermo, L., Chan, P.T. & Cyr, D.G. Alterations in gene expression in the caput epididymides of nonobstructive azoospermic men. *Biol Reprod* **78**, 342-51 (2008).

68. Aso, T. *et al.* Identification and characterization of Elongin A2, a new member of the Elongin family of transcription elongation factors, specifically expressed in the testis. *J Biol Chem* **275**, 6546-52 (2000).

69. Liu, B. *et al.* Common variants of transcription factor 7-like 2 (TCF7L2) are associated with reduced insulin secretion in women with polycystic ovary syndrome. *Gynecol Endocrinol* **28**, 594-7 (2012).

70. Rashid, S. *et al.* Disruption of the murine dynein light chain gene Tcte3-3 results in asthenozoospermia. *Reproduction* **139**, 99-111 (2010).

71. Grima, J., Zhu, L. & Cheng, C.Y. Testin is tightly associated with testicular cell membrane upon its secretion by sertoli cells whose steady-state mRNA level in the testis correlates with the turnover and integrity of inter-testicular cell junctions. *J Biol Chem* **272**, 6499-509 (1997).

72. Aarabi, M., Ousati-Ashtiani, Z., Nazarian, A., Modarressi, M.H. & Heidari, M. Association of TGIFLX/Y mRNA expression with azoospermia in infertile men. *Mol Reprod Dev* **75**, 1761-6 (2008).

73. Wang, X. *et al.* Thoc1 deficiency compromises gene expression necessary for normal testis development in the mouse. *Mol Cell Biol* **29**, 2794-803 (2009).

74. Gruber, M., Mathew, L.K., Runge, A.C., Garcia, J.A. & Simon, M.C. EPAS1 Is Required for Spermatogenesis in the Postnatal Mouse Testis. *Biol Reprod* **82**, 1227-36 (2010).

75. Mashayekhi, F. & Hadiyan, S.P. A single-nucleotide polymorphism in TP53 may be a genetic risk factor for Iranian patients with idiopathic male infertility. *Andrologia* **44 Suppl 1**, 560-4 (2012).

76. Fraga, L.R. *et al.* Interaction between TP63 and MDM2 genes and the risk of recurrent pregnancy loss. *Eur J Obstet Gynecol Reprod Biol* **182C**, 7-10 (2014).

77. Borghei, A. *et al.* Targeted disruption of tyrosylprotein sulfotransferase-2, an enzyme that catalyzes post-translational protein tyrosine O-sulfation, causes male infertility. *J Biol Chem* **281**, 9423-31 (2006).

78. Vinci, G. *et al.* Mutations in the TSPYL1 gene associated with 46,XY disorder of sex development and male infertility. *Fertil Steril* **92**, 1347-50 (2009).

79. Zhang, H. *et al.* Some single-nucleotide polymorphisms of the TSSK2 gene may be associated with human spermatogenesis impairment. *J Androl* **31**, 388-92 (2010).

80. Vogel, P., Hansen, G., Fontenot, G. & Read, R. Tubulin tyrosine ligase-like 1 deficiency results in chronic rhinosinusitis and abnormal development of spermatid flagella in mice. *Vet Pathol* **47**, 703-12 (2010).

81. Duriez, B. *et al.* A common variant in combination with a nonsense mutation in a member of the thioredoxin family causes primary ciliary dyskinesia. *Proc Natl Acad Sci U S A* **104**, 3336-41 (2007).

82. Kim, J.M. *et al.* Inactivation of murine Usp1 results in genomic instability and a Fanconi anemia phenotype. *Dev Cell* **16**, 314-20 (2009).

83. Guo, X. *et al.* Proteomic analysis of proteins involved in spermiogenesis in mouse. *J Proteome Res* **9**, 1246-56 (2010).

84. Wiebe, M.S., Nichols, R.J., Molitor, T.P., Lindgren, J.K. & Traktman, P. Mice deficient in the serine/threonine protein kinase VRK1 are infertile due to a progressive loss of spermatogonia. *Biol Reprod* **82**, 182-93 (2010).

85. Noormets, K. *et al.* Male mice with deleted Wolframin (Wfs1) gene have reduced fertility. *Reprod Biol Endocrinol* **7**, 82 (2009).

86. Li, S., Lu, M.M., Zhou, D., Hammes, S.R. & Morrisey, E.E. GLP-1: a novel zinc finger protein required in somatic cells of the gonad for germ cell development. *Dev Biol* **301**, 106-16 (2007).

87. Yan, W. *et al.* Zmynd15 encodes a histone deacetylase-dependent transcriptional repressor essential for spermiogenesis and male fertility. *J Biol Chem* **285**, 31418-26 (2010).
